# On the Reactivity of (*S*)‐Indoline‐2‐Carboxylic Acid

**DOI:** 10.1002/chir.70008

**Published:** 2024-12-16

**Authors:** Fabiana Cordella, Sophie Faure, Claude Taillefumier, Gennaro Pescitelli, Elisa Martinelli, Giuseppe Alonci, Zhengming Liu, Gaetano Angelici

**Affiliations:** ^1^ Dipartimento di Chimica e Chimica Industriale Università di Pisa Pisa Italy; ^2^ Université Clermont Auvergne, Clermont Auvergne INP, CNRS, ICCF Clermont‐Ferrand France; ^3^ UB‐CARE S.r.l., Spin‐Off University of Pavia Pavia Italy

**Keywords:** (*S*)‐indoline‐2‐carboxylic acid, peptide coupling reaction, sterically hindered amino acid

## Abstract

(*S*)‐Indoline‐2‐carboxylic acid (H‐(2*S*)‐Ind‐OH) possesses the ability to influence the conformation of peptide bonds towards the *cis* amide isomer in polar solvents. However, its potential utilization as a conformational switch within long peptide sequences poses challenges due to its low reactivity and strong inclination to form diketopiperazines. The present study explores its reactivity under various conditions and proposes synthetic strategies to overcome these limitations. A series of H‐(2*S*)‐Ind‐OH containing di‐ and tri‐peptides have been efficiently synthesized and characterized, ready to be inserted in more complex and longer sequences.

## Graphical abstract

A throughout study about the challenging reactivity of (*S*)‐indoline‐2‐carboxylic acid, a nonproteinogenic amino acid with the remarkable ability to induce the conformation of peptide bonds towards the *cis* amide isomer.

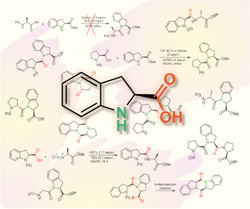

## 1 Introduction

The insertion of novel non‐natural amino acids into peptide sequences is a useful strategy to expand the chemical diversity of peptides, offering several opportunities for designing novel bioactive compounds, probing protein structure–function relationships, and ultimately advancing the frontiers of therapeutic and scientific innovation [1, 2].

It is widely acknowledged that developing diverse peptide coupling conditions is necessary due to the distinct reactivity of amino acids, depending on their structures. Therefore, improving the reaction conditions by testing several coupling reagents, bases, solvents, and additives and preventing side reactions or racemization is essential to develop more efficient activation methods [3, 4, 5].

We have recently studied the distinctive conformational properties of (*S*)‐indoline‐2‐carboxylic acid (H‐(2*S*)‐Ind‐OH), a mimetic of both proline and phenylalanine, showing that Ac‐(2*S*)‐Ind‐OMe possesses a remarkable tendency towards the *cis* amide isomer when dissolved in polar solvents [6]. This behavior contrasts with that typical of proline which mainly forms *trans* prolyl amide bonds, making H‐(2*S*)‐Ind‐OH a good candidate for designing unusual secondary structures. We synthesized H‐(2*S*)‐Ind‐OH homo‐oligomers to obtain the rare polyproline I structure, characterized by a tight right‐handed helix with all amide bonds in the *cis* conformation [7]. Therefore, we speculated that the conformational properties of this particular amino acid could be used, as well, for the formation of *β*‐turns, *β*‐hairpins, or as a conformational switch in more complex sequences.

**SCHEME 1:**
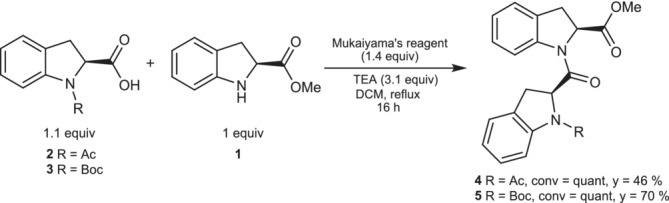
Peptide coupling conditions for synthesizing H‐(2*S*)‐Ind‐OH containing homo‐dipeptides.

The efficient synthesis of (2*S*)‐Ind homo‐oligomers was previously obtained through the use of 2‐chloro‐1‐methylpyridinium iodide (Mukaiyama coupling reagent) activating agent as shown in Scheme 1 [6]. Alternative synthetic attempts performed by using other coupling reagents (HATU, HBTU, DCC, EDC·HCl, PFPA, and PFPP) in different solvent and temperature conditions did not show any reactivity or resulted in just some traces of products. We speculated that the weak nucleophilicity of the aromatic secondary amine group might be the reason for the specific reaction conditions required to couple acetyl‐ and Boc‐*N*‐protected (*S*)‐indoline‐2‐carboxylic acid (**2** and **3**, respectively) with H‐(2*S*)‐Ind‐OMe (**1**).

Developing an efficient synthesis of hetero‐oligomers is necessary to access more complex sequences containing this amino acid. However, our preliminary attempts to couple Boc‐*l*‐Ala‐OH with **1** were more challenging than expected, as it did not work in the experimental conditions developed for the homo‐oligomer synthesis. An elegant and useful strategy to obtain various dipeptides of general formula Boc‐AA‐(2*S*)‐Ind‐OMe has been reported by He et al. and Zheng et al. [8, 9]. The approach is based on a peptide coupling reaction between different *N*‐protected amino acids and phenylalanine methyl ester, followed by an intramolecular C (sp^2^)‐H amination to form the pyrrolidine ring of (2*S*)‐Ind derivatives. The authors justify such a postmodification method because of the inaccessibility of the starting materials and the use of expensive chiral catalysts for its synthesis. However, based on our experience with H‐(2*S*)‐Ind‐OH over the past years, we realized that the limitation of its use in the literature is not related to its accessibility but rather to its low reactivity. H‐(2*S*)‐Ind‐OH is commercially available and affordable depending on the supplier and market demand because of its easy synthesis through common methods like lipase‐catalyzed kinetic resolution [10, 11, 12, 13], catalytic asymmetric hydrogenation [14, 15, 16, 17], and metal‐catalyzed Buchwald–Hartwig cyclization [18, 19, 20]. However, the formation of the indoline ring in dipeptides proposed by He et al. and Zheng et al. requires harsh conditions (T = 110°C–150°C, 8–20 h), which might not be suitable in many peptide synthetic strategies.

Considering the potential of H‐(2*S*)‐Ind‐OH as a proline mimetic for conformational switches in more complex sequences and its application in medicinal chemistry, catalysis, or new materials, we decided to thoroughly study the peptide bond formation on both *N*‐ and *C*‐terminus to determine the synthetic limits of its use and to optimize some strategies to access more complex sequences.

## 2 Results and Discussion

### 2.1 Reactivity of (S)‐Indoline‐2‐Carboxylic Acid Towards Peptide Coupling Reactions

**SCHEME 2:**
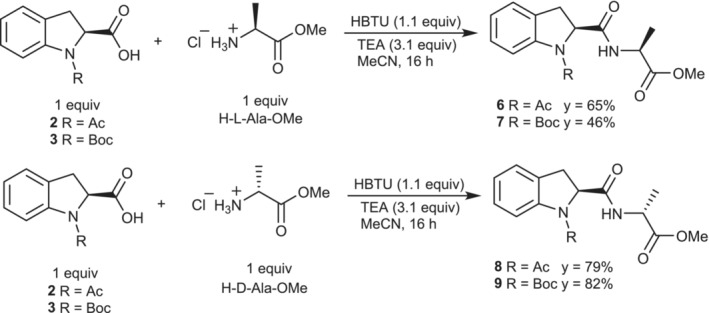
Reaction of **2** and **3** with H‐l‐Ala‐OMe and H‐d‐Ala‐OMe, respectively.

Initially, the reactivity of the carboxylic acid moiety of **2** and **3** with H‐l‐Ala‐OMe and H‐d‐Ala‐OMe hydrochloride was tested under standard conditions using HBTU as peptide coupling reagent (Scheme 2). As expected, while capable of further optimization, the reaction did not present any notable challenges. However, it is interesting to notice the influence on the reactivity of the sterically hindered Boc protecting group in the case of the (l‐l) homochiral dipeptide (**7**), which is obtained in poor yield (y = 46%). In contrast, excellent yields are obtained from the corresponding synthesis of the *(l‐d*) heterochiral dipeptide (**9**). This behavior might suggest the presence of a match‐mismatch enantiomer discrimination effect of the two reaction partners in the transition state.

Furthermore, the ^1^H NMR spectra of products **6** and **8**, in DMSO‐d_6_ (see Supporting Information), showed that the equilibrium between *cis* and *trans* isomers of the acetyl amide bond is predominantly shifted towards the *cis* geometry, as expected from our previous study on the conformational equilibrium of H‐(2*S*)‐Ind‐OH containing homodimer [6].

On the contrary, the coupling reaction between the free amine of **1** and *N*‐protected Alanine proved quite challenging. Consistently with the results already obtained with the synthesis of H‐(2*S*)‐Ind‐OH containing homo‐oligomers [6, 7], classical coupling reagents (HATU, HBTU, DCC, EDC·HCl, PFPA, and PFPP) proved to be ineffective. Therefore, we concentrated on the most promising conditions with Mukaiyama coupling reagent (see Table S1). However, we were surprised to observe that the Mukaiyama coupling reagent was ineffective, regardless of the base used, solvent, and temperature. Moreover, by testing both PG‐l‐Ala‐OH and PG‐d‐Ala‐OH, no significative difference in the reactivity with **1** was observed, excluding any match‐mismatch chiral effect. The desired products Boc‐l‐Ala‐(2*S*)‐Ind‐OMe (**10**) and Boc‐d‐Ala‐(2*S*)‐Ind‐OMe (**11**) were obtained and isolated in poor yields, only using bis(2‐oxo‐3‐oxazolidinyl)phosphinic chloride (BOP‐Cl) or propylphosphonic anhydride (T3P) as peptide coupling reagents (Table 1).

**TABLE 1:** Peptide coupling reaction between PG‐Ala‐OH and **1**.

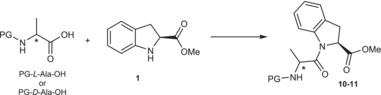						
Product	PG‐Ala‐OH (equiv.)	Base (equiv.)	Coupling reag. (equiv.)	Solvent	*T* (°C)	Yield (%)
**10**	Boc‐l‐Ala‐OH (1)	DIPEA (4)	T3P (2)	EtOAc	rt	/
	Boc‐l‐Ala‐OH (1.2)	DIPEA (4)	T3P (2)	EtOAc	Reflux	5
	Boc‐l‐Ala‐OH (1)	DIPEA (3)	BOP‐Cl (1.2)	DCM	rt	16
	Boc‐l‐Ala‐OH (1.2)	DIPEA (3)	BOP‐Cl (1.4)	DCM	Reflux	17
**11**	Boc‐d‐Ala‐OH (1.2)	DIPEA (4)	T3P (2)	EtOAc	rt	/
	Boc‐d‐Ala‐OH (1.2)	DIPEA (4)	T3P (2)	EtOAc	Reflux	7
	Boc‐d‐Ala‐OH (1.2)	DIPEA (3)	BOP‐Cl (1.4)	DCM	Reflux	11

**FIGURE 1:**
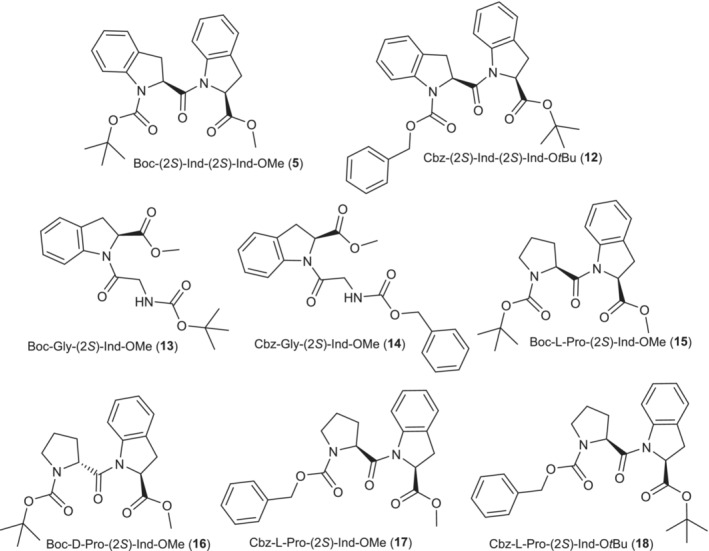
Molecular structures of the products reported in Table 2.

We speculated that the lack of reactivity with Mukaiyama coupling reagent, and the low yields obtained with BOP‐Cl and T3P were due to the steric hindrance caused by the methyl side chain of alanine and the indoline aromatic ring. This hindrance could impede an effective nucleophilic attack of the indoline nitrogen on the activated ester. Further experiments were performed to validate our hypothesis. Primarily, we attempted to couple **1** with *N‐*protected glycines, aiming to mitigate any potential steric hindrance posed by the methyl group of alanine. Additionally, we explored coupling reactions with *N*‐protected l‐proline and d‐proline, which exhibit a distorted χ angle of the pyrrolidine ring akin to the H‐(2*S*)‐Ind‐OH one. As expected, we observed that the coupling reaction worked effectively with *N*‐protected‐Gly‐OH, *N*‐protected‐l‐Pro‐OH, and d‐Pro‐OH using both Mukaiyama reagent and T3P coupling reagent [21, 22], leading to satisfactory yields as shown in Table 2. The products obtained are depicted in Figure 1. Regarding the use of T3P, besides the improved sustainability associated with this reagent, another notable advantage is its simplified purification process. The Mukaiyama reagent, mostly used in solid‐phase synthesis, also forms 1‐methyl‐2‐pyridone as by‐product, which was challenging to separate from the synthesized dipeptides through column chromatography, often leading to reduced final yields in obtaining pure compounds.

**TABLE 2:** Synthesis of H‐(2*S*)‐Ind‐OH containing dipeptides.

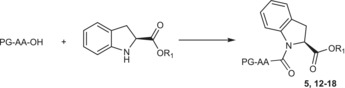							
Product	PG‐AA‐OH	−OR_1_	Base	Coupling reag.	Solvent	*T* (°C)	Yield^c^
**5**	Boc‐(2*S*)‐Ind‐OH	−OMe	DIPEA	T3P^a^	EtOAc	Reflux	40
**12**	Cbz‐(2*S*)‐Ind‐OH	−O*t*Bu	TEA	Mukaiy^b^	DCM	Reflux	65
**13**	Boc‐Gly‐OH	−OMe	DIPEA	T3P^a^	EtOAc	rt	82
			TEA	Mukaiy^b^	DCM	Reflux	56
			TEA	Mukaiy^b^	DMF	80	54
**14**	Cbz‐Gly‐OH	−OMe	DIPEA	T3P^a^	EtOAc	rt	70
			TEA	Mukaiy^b^	DCM	Reflux	47
**15**	Boc‐l‐Pro‐OH	−OMe	DIPEA	T3P^a^	EtOAc	Reflux	55
			TEA	Mukaiy^b^	DCM	Reflux	50
			TEA	Mukaiy^b^	DMF	80	40
**16**	Boc‐d‐Pro‐OH	−OMe	TEA	Mukaiy^b^	DCM	Reflux	26
			TEA	Mukaiy^b^	DMF	80	20
**17**	Cbz‐l‐Pro‐OH	−OMe	DIPEA	T3P^a^	EtOAc	Reflux	47
			TEA	Mukaiy^b^	DCM	Reflux	65
			TEA	Mukaiy^b^	DMF	80	62
**18**	Cbz‐l‐Pro‐OH	−O*t*Bu	TEA	Mukaiy^b^	DCM	Reflux	50

^a^ Reaction conditions with T3P: PG‐AA‐OH (1 equiv.), **1** (1 equiv.), T3P in 50% wt/wt EtOAc (2 equiv.), DIPEA (4 equiv.).

^b^ Reaction conditions with Mukaiyama reagent: PG‐AA‐OH (1.1 equiv.), **1** (1 equiv.), Mukaiyama reagent (1.4 equiv.), TEA (2.8 equiv.).

^c^ Isolated yield.

**FIGURE 2:**
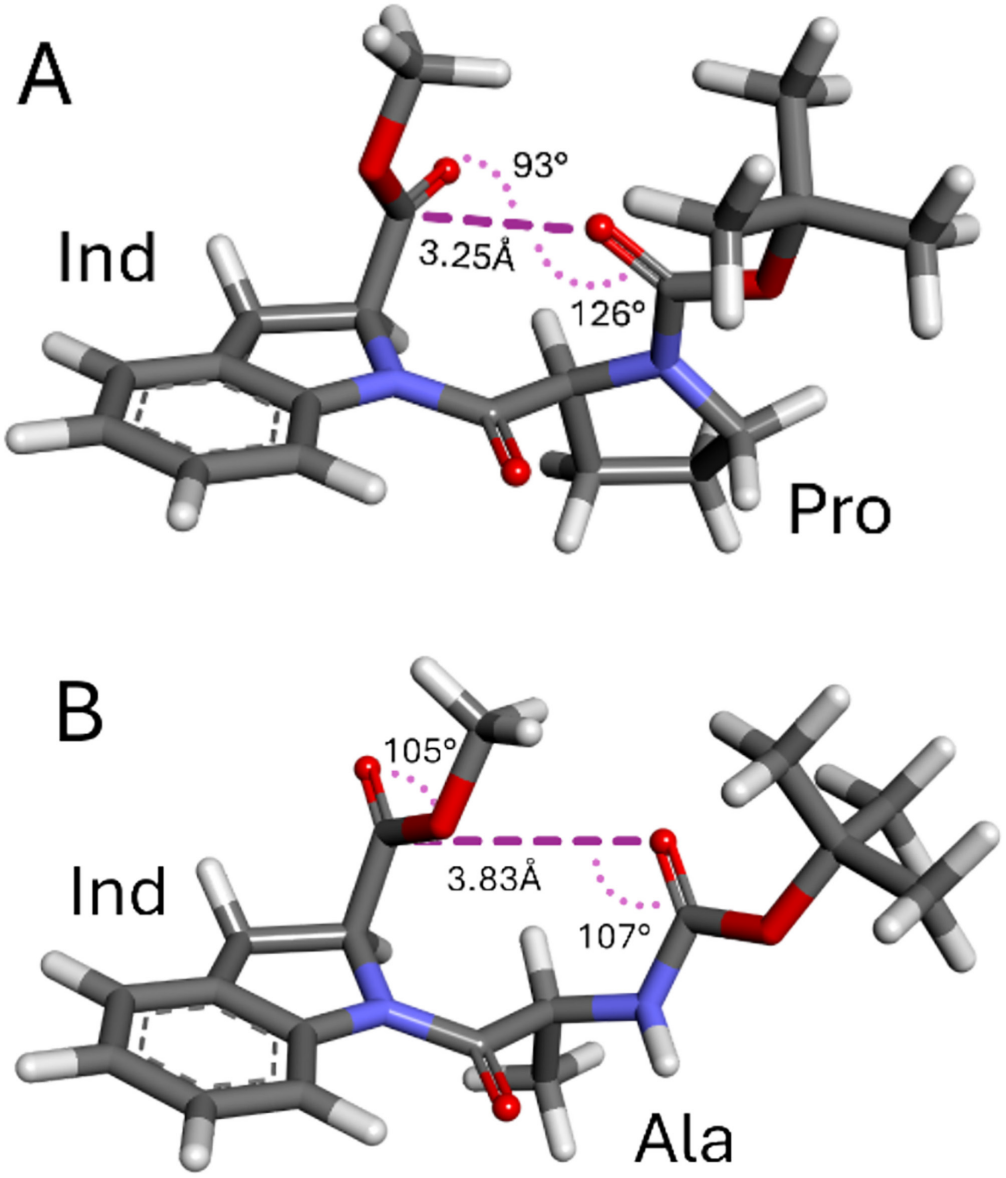
Lowest energy structures of **15** (A) and **10** (B) with relevant measurements for the n_C=O_ → π*_COO_ interaction, which is possible only in the first case. The structures represent the lowest‐energy conformers obtained at B3LYP‐D3/6‐31+G(d) level of calculation.

A full rationalization of the observed reactivity would require a computational investigation of the reactive species formed with the coupling reagent and possible several reaction intermediates. On a simplified ground, we noticed that the products Boc‐l‐Pro‐(2*S*)‐Ind‐OMe (**15**) and Boc‐l‐Ala‐(2*S*)‐Ind‐OMe (**10**) present an interesting structural difference (Figure 2).

In compound **15**, a n_C=O_ → π*_COO_ interaction is possible between the carbamate and ester group, similar to the well‐known stabilizing interaction of proline derivatives [6, 23, 24]. By contrast, the same interaction is precluded in **10**, where the two carbonyl groups lie almost parallel to each other. Such stabilization may concur, at least in part, to the observed reactivity.

Summarizing, under the optimized reaction conditions, it is possible to synthesize dipeptides of the general formula PG‐AA‐(2*S*)‐Ind‐OMe, with AA = Gly, H‐(2*S*)‐Ind‐OH, and l‐Pro or d‐Pro with good yields, while the reaction with AA = l‐Ala o d‐Ala proceeds only in poor yields possibly because of steric reasons. This is an important piece of information because any synthetic strategy for synthesizing long sequences containing H‐(2*S*)‐Ind‐OH will have to consider the coupling of this amino acid at an early stage, followed by a block‐wise synthesis. Alternatively, a H‐(2*S*)‐Ind‐OH containing dipeptide could be synthesized by the intramolecular pyrrolidine formation approach proposed by He et al. and Zheng et al. [8, 9], which however will have to consider a block‐wise synthesis for longer peptides.

### 2.2 Diketopiperazine Formation

The possibility of introducing H‐Gly‐(2*S*)‐Ind‐OH, H‐l‐Pro‐(2*S*)‐Ind‐OH, H‐d‐Pro‐(2*S*)‐Ind‐OH, or H‐(2*S*)‐Ind‐(2*S*)‐Ind‐OH in longer peptide sequences as *β*‐turn or *β*‐hairpin inducer motifs or as a scaffold to improve peptide macrocyclization reactions motivated us to test the possibility to perform a 2 + 2 block‐wise oligomer synthesis. Moreover, we were interested in the synthesis of the deprotected dipeptide H‐(2*S*)‐Ind‐(2*S*)‐Ind‐OH because it represents an interesting constrained mimetic of H‐l‐Phe‐l‐Phe‐OH, a dipeptide widely known for its remarkable self‐assembly properties and used in several applications [25, 26, 27, 28, 29, 30, 31, 32].

However, we were amazed by the almost immediate formation of the 2,5‐diketopiperazine from Boc‐(2*S*)‐Ind‐(2*S*)‐Ind‐OR as a consequence of the *N*‐terminus deprotection, even when the *C*‐terminus was protected as a *tert*‐butyl ester, usually used to avoid the formation of diketopiperazines, or the free carboxylic acid, to yield a bad leaving group. The reaction led to a complete conversion to diketopiperazine (**19**), regardless of the *N*‐protecting group installed on the *N*‐terminus of the homo‐dipeptide and related cleavage conditions used, in acidic, basic, or neutral conditions, as shown in Table 3.

**TABLE 3:** 2,5‐Diketopiperazine **19** formation from the *N*‐terminus deprotection of H‐(2*S*)‐Ind‐OH homo‐dimers.

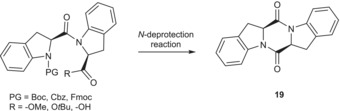					
Product	Protected homo‐dimer	Deprotection conditions	Solvent	Conv. (%)^a^	Yield (%)
**19**	Cbz‐((2*S*)‐Ind)_2_‐OMe	H_2_ (1 atm), 10% Pd/C, 18 h	MeOH	Quant.	Quant.
	Fmoc‐((2*S*)‐Ind)_2_‐OMe	LiOH·H_2_O (3 equiv.), 3 h	Dioxane/H_2_O	Quant.	72
	Boc‐((2*S*)‐Ind)_2_‐OMe	TFA (8 equiv.), 5 h	DCM	Quant.	/^b^
	Boc‐((2*S*)‐Ind)_2_‐OH	TFA (8 equiv.), 5 h	DCM	Quant.	43^c^
	Cbz‐((2*S*)‐Ind)_2_‐O*t*Bu	H_2_ (1 atm),10% Pd/C, 18 h	MeOH	Quant.	/^b^

^a^ The reaction was monitored by TLC and UPLC‐MS to confirm the complete conversion of the precursor into the relative 2,5‐DKP.

^b^ Complete conversion to **19** was confirmed by TLC and UPLC‐MS analysis, but the product was not isolated.

^c^ The low yield after chromatography in this case derives from the tendency of **19** to aggregate.

Diketopiperazine formation is a well‐known reaction occurring at the level of dipeptides, especially with the involvement of a *cis*‐inducing peptide bond amino acid like proline. However, this phenomenon is extremely favored using H‐(2*S*)‐Ind‐OH in any conditions, confirming this amino acid's remarkable conformational properties. On the one hand, the easy formation of diketopiperazines limits the synthetic strategies to insert H‐(2*S*)‐Ind‐OH into peptide sequences in solution; on the other hand, such synthesis of diketopiperazines in very mild conditions could provide easy access to a class of molecular scaffolds with many interesting biological properties in medicinal chemistry [33] or as new materials [34].

Therefore, we explored the possibility of synthesizing asymmetric diketopiperazines from the *N*‐deprotection of the synthesized hetero‐dipeptides **13**, **14**, **15**, **17**, and **18** (Table 4).

**TABLE 4:** Asymmetric 2,5‐diketopiperazine formation from *N*‐deprotection of H‐(2*S*)‐Ind‐OH containing hetero‐dipeptides.

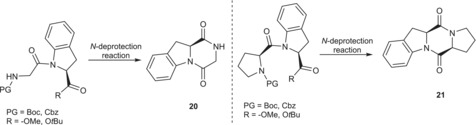					
Product	Protected dipeptides	Deprotection conditions	Solvent	Conv. (%)^a^	Yield (%)
**20**	Boc‐Gly‐(2*S*)‐Ind‐OMe (**13**)	TFA (8 equiv.), 5 h	DCM	Quant.	70
	Cbz‐Gly‐(2*S*)‐Ind‐OMe (**14**)	H_2_ (1 atm), Pd/C 10%, 18 h	MeOH	Quant.	57
**21**	Cbz‐l‐Pro‐(2*S*)‐Ind‐OMe (**17**)	H_2_ (1 atm), Pd/C 10%, 18 h	MeOH	Quant.	91
	Boc‐l‐Pro‐(2*S*)‐Ind‐OMe (**15**)	TFA (8 equiv.), 5 h	DCM	Quant.	/^b^
	Cbz‐l‐Pro‐(2*S*)‐Ind‐OtBu (**18**)	H_2_ (1 atm), Pd/C 10%, 18 h	MeOH	Quant.	/^b^

^a^ Conversion was followed by UPLC‐MS injections until completion.

^b^ Complete conversion to **20** or **21** was confirmed by TLC and UPLC‐MS analysis, but the product was not isolated.

The results reported in Table 4 show the high tendency of the scaffolds H‐Gly‐(2*S*)‐Ind‐OH and H‐l‐Pro‐(2*S*)‐Ind to form the corresponding diketopiperazines, both in acidic conditions for the Boc deprotection with TFA and in neutral conditions by hydrogenolysis for the cleavage of the Cbz protecting group, regardless of the nature of the ester used on the *C*‐terminus, that is, methyl or *tert*‐butyl ester. Therefore, a secure strategy for a modular synthesis in solution of longer peptides must be aware of such undesired reactivity, as it is not possible to perform the *N*‐deprotection on a dipeptide of general formula PG‐AA‐(2*S*)‐Ind‐OR avoiding diketopiperazine formation.

### 2.3 Synthesis of Cbz‐l‐Pro‐(2*S*)‐Ind‐l‐Pro‐OMe (**23**)

**SCHEME 3:**
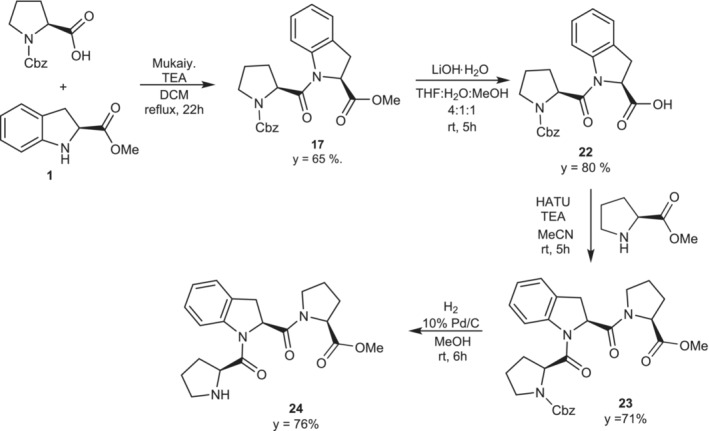
Synthesis of Cbz‐ l‐Pro‐(2*S*)‐Ind‐ l‐Pro‐OMe (**23**) and the corresponding *N*‐deprotected product (**24**).

As mentioned above, a synthetic strategy for the insertion of H‐(2*S*)‐Ind‐OH into a peptide sequence needs to consider the demonstrated unfeasibility to perform a 2 + 2 block‐wise oligomer synthesis or in general to avoid the deprotection at the *N*‐terminus of a H‐(2*S*)‐Ind‐OH containing dipeptide protected as an ester at the *C*‐terminus. Therefore, we decided to perform a classical modular approach for synthesizing the tripeptide Cbz‐l‐Pro‐(2*S*)‐Ind‐l‐Pro‐OMe (**23**) and to test the deprotection of the terminal amine function to exclude the formation of 2,5‐diketopiperazine. Such testing is necessary to comprehend the feasibility of inserting H‐(2*S*)‐Ind‐OH in longer sequences as an efficient conformational switch. As shown in Scheme 3, the synthesis of the dipeptide **17** achieved a good yield through the previously optimized method using the Mukaiyama coupling reagent. The methyl ester of dipeptide **17** was deprotected through the classical saponification method using lithium hydroxide to obtain the intermediate **22**. Subsequently, **22** was coupled with H‐l‐Pro‐OMe using HATU as coupling reagent, giving tripeptide **23**, with a good yield, which was deprotected at the *N*‐terminus through palladium‐catalyzed hydrogenolysis of the Cbz‐protecting group. The free *N*‐terminus tripeptide **24** was easily obtained with a good yield, fully characterized, and ready for further coupling reactions.

As expected, diketopiperazine does not occur during the deprotection of **23** on the *N*‐terminus, giving **24** as the only reaction product. Such a result allowed us to conclude that longer (2*S*)‐Ind‐OH‐containing sequences can be synthesized through a block‐wise approach, but the (2*S*)‐Ind‐containing *N*‐terminus partner of the reaction must be at least a trimer.

## 3 Materials and Methods

All nonaqueous reactions were run in oven‐dried glassware under a positive pressure of argon or nitrogen, with exclusion of moisture from reagents and glassware, transferring solvents and liquid reagents with hypodermic syringes. The glassware has been dried with a heating gun under vacuum and allowed to cool under argon. Anhydrous solvents and liquid reagents were obtained using standard drying techniques. Solid reagents were of commercially available grade, used without further purification, and, when necessary, stored in a controlled atmosphere and/or at −20°C. (*S*)‐Indoline‐2‐carboxylic acid was purchased from abcr GmbH. Microwave‐assisted reactions were performed by using a CEM/Discovery 2.0 instrument. Reactions were monitored by thin‐layer chromatography using Merck silica gel 60 F254 plates. The chromatogram developed was visualized by UV absorbance, aqueous potassium permanganate, or iodine. Flash chromatography was performed using Sigma‐Aldrich silica gel 60, particle size of 40–63 μm, with the indicated solvent system. Melting points were measured using a “Büchi Melting Point B‐545” instrument. ^1^HNMR and ^13^CNMR were measured on a Jeol instrument JNM‐ECZ400R or JNM‐ECZ500R and a Bruker AC‐400 spectrometer. Chemical shifts are reported in ppm with the deuterated solvent signal as the internal standard. Data are reported as follows: chemical shift, integration, and multiplicity (s = singlet, d = doublet, t = triplet, q = quartet, qn = quintet, m = multiplet, and br = broad), with the coupling constant in hertz.

The HPLC‐DAD analyses for **6**–**9**, **13**, and **14** were performed with a Jasco International Co. (Tokyo, Japan) system composed by a PU‐2089 quaternary pump with a degasser, an AS‐950 autosampler, and an MD‐2010 spectrophotometric DAD detector operating in 200–650 nm wavelength range with a 4 nm resolution, and a 0.2 s acquisition rate. JASCO ChromNav software was used for data acquisition and analysis. The HPLC‐ESI‐HRMS analyses for **6**–**9**, **13**, and **14** were performed with an HPLC 1200 Infinity, a Jet Stream ESIQ‐ToF 6530 Infinity detector, and an Agilent Infinity autosampler (Agilent Technologies, Palo Alto, CA, USA). HRMS spectra were obtained in the acquisition range of 100–1700 m/z at a scan rate of 1.04 spectra per second. MassHunter Workstation Software (B.10.00) was used for data acquisition and analysis. Both instrumentations were composed of an analytical reversed‐phase column Poroshell 120 EC‐C18 (3.0 × 75 mm, particle size 2.7 μm), equipped with a Poroshell 120 EC‐C18 (3.0 × 5 mm, particle size 2.7 μm) guard column, by Agilent Technologies (Palo Alto, CA, USA). The eluents used as mobile phase were A = formic acid (FA) 1% (v/v) in water and B = formic acid (FA) 0.3% (v/v) in acetonitrile. The column was kept at 30°C, with a flow rate of 0.6 mL/min and the following gradient: 5% B for 2.6 min, then to 50% B in 13.0 min, to 70% B in 5.2 min, to 100% B in 6.2 min and then hold for 3.0 min; re‐equilibration took 11 min.

The HPLC‐DAD analyses for **12**, **17**, **18**, **23**, and **24** were performed with a Agilent 1100 series coupled to DAD detector equipped with an NUCLEODUR 100‐3 (C18, 125 mm, Ø 4.6 mm) with a flow of 1 mL/min using as Solvent A: water (0.1% TFA) and Solvent B: MeCN; Gradient for analytical HPLC (17 min runs): 5% B (0–2 min), 5 → 95% B (2–9 min), 95% B (9–12 min), 95 → 5% B (12–14 min), 5% B (14–17 min). HPLC‐ESI‐HRMS analyses for **12**, **17**, **18**, **23**, and **24** were performed with a Q Exactive Quadrupole‐Orbitrap Mass Spectrometer coupled to a UPLC Ultimate 3000 (Kinetex EVO C18; 1,7 μm; 100 × 2.1 mm column with a flow rate of 0.45 mL/min with the following gradient: a linear gradient of Solvent B from 5% to 95% over 7.5 min (Solvent A = H_2_O + 0.1% formic acid, Solvent B = acetonitrile + 0.1% formic acid) equipped with a DAD UV/VIS 3000 RS detector).

### 3.1 General Method A: Synthesis of PG‐(2*S*)‐Ind‐AA‐OMe Dipeptides (6–9)

In a two‐neck round‐bottom flask, under nitrogen atmosphere, **2** or **3** (1 equiv.) and HBTU (1.1 equiv.) were dissolved in dry MeCN and let stir for 15 min. A mixture containing H‐l‐Ala‐OMe·HCl or H‐d‐Ala‐OMe·HCl (1 equiv.) and TEA (3.1 equiv.) was added dropwise. The reaction mixture was stirred at room temperature for 16 h. The solvent was removed under reduced pressure and the residue was re‐dissolved in DCM and washed with 1 M HCl, a saturated solution of NaHCO_3_ and brine. The organic phase was dried over Na_2_SO_4_ and filtered. The solvent was removed under reduced pressure. The crude product was purified by flash chromatography.

### 3.2 General Method B: Synthesis of Boc‐l‐Ala‐(2*S*)‐Ind‐OMe (10) and Boc‐d‐Ala‐(2*S*)‐Ind‐OMe (11) With BOP‐Cl Coupling Reagent

Boc‐l‐Ala or Boc‐d‐Ala (1.2 equiv.) was dissolved in dry DCM in a two‐neck round‐bottom flask equipped with a refrigerator under nitrogen atmosphere. The solution was cooled at 0°C and DIPEA (2 equiv.) and BOP‐Cl (1.4 equiv.) were added. The mixture was warmed at room temperature for 30 min and then cooled again at 0°C, to add **1** (1 equiv.), DIPEA (1 equiv.), and dry DCM. The reaction mixture was warmed to room temperature and stirred under reflux for 22 h. The reaction was cooled down at room temperature, and after dilution with DCM, it was washed with H_2_O and brine. The organic phase was dried over Na_2_SO_4_ and filtered, and the solvent was removed under reduced pressure. The crude product was purified by flash chromatography.

### 3.3 General Method C: Synthesis of PG‐AA‐(2*S*)‐Ind‐OR Dipeptides (12, 16–18) With Mukaiyama Coupling Reagent

In a two‐neck round‐bottom flask equipped with a refrigerator under argon atmosphere, *N*‐protected amino acid (1.1 equiv.) was dissolved in dry DCM or DMF. Mukaiyama reagent (1.4 equiv.) was added, and TEA (2.8 equiv.) was dropped slowly into the stirred solution. H‐(2S)‐Ind‐OMe **1** or H‐(2S)‐Ind‐O*t*Bu (1 equiv.) was added, and the reaction mixture was stirred under reflux in DCM or at 80°C in DMF. The reaction was monitored by TLC and in some cases by UPLC‐MS analysis. The reaction mixture was cooled down at room temperature, and after dilution with DCM, it was washed with 1 M HCl, a saturated solution of NaHCO_3_ and brine. The organic phase was dried over Na_2_SO_4_ and filtered, and the solvent was removed under reduced pressure. The crude product was purified by flash chromatography.

### 3.4 General Method D: Synthesis of PG‐AA‐(2*S*)‐Ind‐OR Dipeptides (5, 13–15) With T3P Coupling Reagent

In a two‐neck round‐bottom flask, under nitrogen atmosphere, **1** (1 equiv.) and DIPEA (4 equiv.) were added to a solution of *N*‐protected amino acid (1 equiv.) in dry EtOAc. Lastly, a 50% wt solution of T3P (2 equiv.) was added dropwise. According to the *N*‐protected amino acid used, the reaction mixture was stirred overnight at room temperature or reflux, as specified in the Supporting Information. The reaction was quenched by the addition of H_2_O and extracted with EtOAc. The organic phase was washed with brine, dried over Na_2_SO_4_, and filtered. The solvent was removed under reduced pressure, and the crude product was purified by flash chromatography.

### 3.5 General Method E: Synthesis of 2,5‐Diketopiperazines (19–21) Through Cbz‐ or Boc‐Protecting Group Cleavage

#### 3.5.1 Removal of Cbz

In a two‐neck round‐bottom flask, 10%wt palladium on charcoal was added to a mixture of **12** or **17** in dry MeOH. The mixture was stirred overnight at room temperature under hydrogen atmosphere. The reaction mixture was filtered on a celite pad, and the residue was washed with MeOH. The solvent was removed under reduced pressure to afford the 2,5‐DKPs **19** or **21** as the only reaction product.

#### 3.5.2 Removal of Boc

In a two‐neck round‐bottom flask under nitrogen atmosphere, **13** was dissolved in dry DCM. TFA (8 equiv.) was added drop by drop and the mixture was stirred at room temperature for 5 h. The solvent was removed under reduced pressure, and the residue was dissolved in DCM and washed with a saturated solution of NaHCO_3_ and brine. The collected organic phases were dried over Na_2_SO_4_ and filtered. The solvent was removed under reduced pressure to afford the 2,5‐DKPs **20** as the only reaction product.

### 3.6 Synthesis of Cbz‐l‐Pro‐(2*S*)‐Ind‐l‐Pro‐OMe (23) and H‐l‐Pro‐(2*S*)‐Ind‐l‐Pro‐OMe (24)

In a one‐neck round‐bottom flask, **17** (1 equiv.) was dissolved in a solution of THF/H_2_O/MeOH 4:1:1. LiOH·H_2_O (3 equiv.) was added, and the reaction mixture was stirred at room temperature for 5 h and monitored by TLC. The mixture was acidified with 1 M HCl and extracted with EtOAc. The organic phase was dried over Na_2_SO_4_, filtered, and the solvent removed under reduced pressure to recover the crude product **22** without further purification.

Afterwards, in a two‐neck round bottom flask, under nitrogen atmosphere, **22** (1 equiv.) was dissolved in dry MeCN and TEA (3.1 equiv.) and HATU (1.1 equiv.) were added. The mixture was stirred for 15 min at room temperature, and a solution of l‐Pro‐OMe (1 equiv.) in dry MeCN was added dropwise. The reaction mixture was stirred at room temperature for 6 h. The mixture was diluted with DCM and washed with 1 M HCl, a saturated solution of NaHCO_3_ and brine. The organic phase was dried over Na_2_SO_4_ and filtered. The solvent was removed under reduced pressure. The crude product was purified by flash chromatography to afford product **23**.

In a two‐neck round‐bottom flask, 10% wt palladium on charcoal was added to a solution of **23** in dry MeOH. The mixture was stirred at room temperature under hydrogen atmosphere for 6 h. The reaction mixture was filtered on a celite pad, and the residue was washed with MeOH. The solvent was removed under reduced pressure to afford the product **24** without further purification.

## 4 Conclusion

(*S*)‐Indoline‐2‐carboxylic acid possesses the useful property of switching the geometry of peptide bonds towards the *cis* amide isomer when dissolved in polar solvents. Such a feature could be beneficial for the formation of *β*‐ or *γ*‐turns, for the convenient synthesis of cyclopeptides, or for the synthesis of non‐natural new secondary structures. However, such applications cannot be developed without an understanding of its reactivity. As a matter of fact, the same desirable conformational properties of H‐(2*S*)‐Ind‐OH intrinsically limit its use, due to the scarce reactivity of the aromatic amine at the *N*‐terminus and the high propensity of H‐(2*S*)‐Ind‐OH‐containing dipeptides to form diketopiperazines. Herein, we reported a comprehensive reactivity study of coupling reactions under various conditions and proposed synthetic strategies to overcome these limitations. We found an efficient method to couple H‐(2*S*)‐Ind‐OH on the *N*‐terminus to itself, l‐proline, d‐proline, and glycine, but more hindered amino acids like alanine still pose a major challenge and were obtained in low yield. The obtained hetero‐dimers were found to have a high tendency to form the corresponding diketopiperazines in very good yields, when deprotected to the *N‐*terminus, regardless of the nature of the ester at the *C*‐terminus, giving access to interesting molecular scaffolds. Therefore, the insertion of H‐(2*S*)‐Ind‐OH into a peptide sequence in solution requires a modular approach to avoid the formation of diketopiperazine. We synthesized the tripeptide Cbz‐l‐Pro‐(2*S*)‐Ind‐l‐Pro‐OMe in very good yields and deprotected the *N*‐terminus, showing that the product is stable and ready to be coupled into more complex sequences.

## Author Contributions

**Fabiana Cordella:** investigation, writing – original draft, methodology, validation, visualization, data curation. **Sophie Faure:** funding acquisition, investigation, methodology, validation, supervision. **Claude Taillefumier:** funding acquisition, investigation, supervision, validation, methodology. **Gennaro Pescitelli:** investigation, validation, data curation, supervision, formal analysis. **Elisa Martinelli:** methodology, validation, visualization, supervision. **Giuseppe Alonci:** data curation, methodology, funding acquisition, validation, supervision. **Zhengming Liu:** investigation. **Gaetano Angelici:** conceptualization, funding acquisition, writing – original draft, methodology, supervision, data curation, visualization.

## Supporting information


**Table S1** Synthesis of H‐(2*S*)‐Ind‐OH containing dipeptides.

## Data Availability

^1^HNMR and ^13^CNMR raw data are openly available, as FID files, in figshare repository, at https://doi.org/10.6084/m9.figshare.27609684.v1.
