# Supplementary material for: On the Reactivity of (S)‐Indoline‐2‐Carboxylic Acid
Source: Chirality. 2024 Dec 16;36(12):e70008. doi: 10.1002/chir.70008 (PMC11649396; doi:10.1002/chir.70008)
Supplement: Supplementary file 1 — Table S1 Synthesis of H‐(2S)‐Ind‐OH containing dipeptides. [file CHIR-36-e70008-s001.docx]

**Supporting Information**

On the reactivity of (*S*)-indoline-2-carboxylic acid

Fabiana Cordella^*[a]^, Sophie Faure^[b]^, Claude Taillefumier^[b]^, Gennaro Pescitelli^[a]^, Elisa Martinelli^[a]^, Giuseppe Alonci^[c]^, Zhengming Liu^[a]^, Gaetano Angelici^*[a]^

^[a]^ Dipartimento di Chimica e Chimica Industriale, Università di Pisa, 56124, Pisa, Italy

^[b]^ Université Clermont Auvergne, CNRS, ICCF, 63000, Clermont-Ferrand, France

^[c]^ UB-CARE S.r.l., Spin-Off University of Pavia, 27100 Pavia, Italy

**Index**

[1 Peptide coupling reaction between (2*S*)-Ind-OMe and PG-Ala-OH 1](#_Toc181632611)

[2 Synthesis 2](#_Toc181632612)

[1.1 General method A 2](#_Toc181632613)

[Ac-(2*S*)-Ind-l-Ala-OMe (**6**) 2](#_Toc181632614)

[Boc-(2*S*)-Ind-l-Ala-OMe (**7**) 2](#_Toc181632615)

[Ac-(2*S*)-Ind-d-Ala-OMe (**8**) 2](#_Toc181632616)

[Boc-(2*S*)-Ind-d-Ala-OMe (**9**) 2](#_Toc181632617)

[1.2 General method B 3](#_Toc181632618)

[Boc-l-Ala-(2*S*)-Ind-OMe (**10**) 3](#_Toc181632619)

[Boc-d-Ala-(2*S*)-Ind-OMe (**11**) 3](#_Toc181632620)

[1.3 General method C 3](#_Toc181632621)

[Cbz-((2*S*)-Ind)_2_-O*t*Bu (**12**) 3](#_Toc181632622)

[Boc-d-Pro-(2*S*)-Ind-OMe (**16**) 4](#_Toc181632623)

[Cbz-l-Pro-(2*S*)-Ind-OMe (**17**) 4](#_Toc181632624)

[Cbz-l-Pro-(2*S*)-Ind-O*t*Bu (**18**) 4](#_Toc181632625)

[1.4 General method D 4](#_Toc181632626)

[Boc-((2*S*)-Ind)_2_-OMe (**5**) 4](#_Toc181632627)

[Boc-Gly-(2*S*)-Ind-OMe (**13**) 5](#_Toc181632628)

[Cbz-Gly-(2*S*)-Ind-OMe (**14**) 5](#_Toc181632629)

[Boc-l-Pro-(2*S*)-Ind-OMe (**15**) 5](#_Toc181632630)

[1.5 2,5-diketopiperazine formation 5](#_Toc181632631)

[Diketopiperazine **19** 5](#_Toc181632632)

[Diketopiperazine **20** 6](#_Toc181632633)

[Diketopiperazine **21** 6](#_Toc181632634)

[1.6 Synthesis of Cbz-l-Pro-(2*S*)-Ind-l-Pro-OMe **(23)** and the *N*-deprotected product **(24)** 6](#_Toc181632635)

[Cbz-l-Pro-(2*S*)-Ind-l-Pro-OMe (**23**) 6](#_Toc181632636)

[HN-l-Pro-(2*S*)-Ind-l-Pro-OMe (**24**) 6](#_Toc181632637)

[3 Reference 7](#_Toc181632638)

[4 NMR spectra 8](#_Toc181632639)

[^1^H NMR of Ac-(2*S*)-Ind-l-Ala-OMe (**6**) in CDCl_3_ 8](#_Toc181632640)

[^13^C NMR of Ac-(2*S*)-Ind-l-Ala-OMe (**6**) in CDCl_3_ 9](#_Toc181632641)

[^1^H NMR of Ac-(2*S*)-Ind-l-Ala-OMe (**6**) in DMSO-d_6_ 10](#_Toc181632642)

[^1^H NMR of Boc-(2*S*)-Ind-l-Ala-OMe (**7**) in CDCl_3_ 11](#_Toc181632643)

[^13^C NMR of Boc-(2*S*)-Ind-l-Ala-OMe (**7**) in CDCl_3_ 12](#_Toc181632644)

[^1^H NMR of Ac-(2*S*)-Ind-d-Ala-OMe (**8**) in CDCl_3_ 13](#_Toc181632645)

[^13^C NMR of Ac-(2*S*)-Ind-d-Ala-OMe (**8**) in CDCl_3_ 14](#_Toc181632646)

[^1^H NMR of Ac-(2*S*)-Ind-d-Ala-OMe (**8**) in DMSO-d_6_ 15](#_Toc181632647)

[^1^H NMR of Boc-(2*S*)-Ind-d-Ala-OMe (**9**) in CDCl_3_ 16](#_Toc181632648)

[^13^C NMR of Boc-(2*S*)-Ind-d-Ala-OMe (**9**) in CDCl_3_ 17](#_Toc181632649)

[^1^H NMR of Boc-l-Ala-(2*S*)-Ind-OMe (**10**) in CDCl_3_ 18](#_Toc181632650)

[^13^C NMR of Boc-l-Ala-(2*S*)-Ind-OMe (**10**) in CDCl_3_ 19](#_Toc181632651)

[^1^H NMR of Boc-d-Ala-(2*S*)-Ind-OMe (**11**) in CDCl_3_ 20](#_Toc181632652)

[^13^C NMR of Boc-d-Ala-(2*S*)-Ind-OMe (**11**) in CDCl_3_ 21](#_Toc181632653)

[^1^H NMR Cbz-((2*S*)-Ind)_2_-OtBu (**12**) in CDCl_3_ 22](#_Toc181632654)

[DEPT ^13^C NMR Cbz-((2*S*)-Ind)_2_-OtBu (**12**) in CDCl_3_ 23](#_Toc181632655)

[^1^H NMR of Boc-Gly-(2*S*)-Ind-OMe (**13**) in CDCl_3_ 24](#_Toc181632656)

[^13^C NMR of Boc-Gly-(2*S*)-Ind-OMe (**13**) in CDCl_3_ 25](#_Toc181632657)

[^1^H NMR of Cbz-Gly-(2*S*)-Ind-OMe (**14**) in CDCl_3_ 26](#_Toc181632658)

[^13^C NMR of Cbz-Gly-(2*S*)-Ind-OMe (**14**) in CDCl_3_ 27](#_Toc181632659)

[^1^ HNMR of Boc-l-Pro-(2*S*)-Ind-OMe (**15**) in CDCl_3_ 28](#_Toc181632660)

[^13^C NMR of Boc-l-Pro-(2*S*)-Ind-OMe (**15**) in CDCl_3_ 29](#_Toc181632661)

[^1^H NMR of Boc-d-Pro-(2*S*)-Ind-OMe (**16**) in CDCl_3_ 30](#_Toc181632662)

[^13^C NMR of Boc-d-Pro-(2*S*)-Ind-OMe (**16**) in CDCl_3_ 31](#_Toc181632663)

[^1^H NMR of Cbz-l-Pro-(2*S*)-Ind-OMe (**17**) in CDCl_3_ 32](#_Toc181632664)

[^13^C NMR of Cbz-l-Pro-(2*S*)-Ind-OMe (**17**) in CDCl_3_ 33](#_Toc181632665)

[^1^H NMR of Cbz-l-Pro-(2*S*)-Ind-OtBu (**18**) in CDCl_3_ 34](#_Toc181632666)

[^13^C NMR of Cbz-l-Pro-(2*S*)-Ind-OtBu (**18**) in CDCl_3_ 35](#_Toc181632667)

[^1^H NMR of 2,5-diketopiperazine: Product **19** in CDCl_3_ 36](#_Toc181632668)

[^1^H NMR of 2,5-diketopiperazine: Product **20** in CDCl_3_ 37](#_Toc181632669)

[^13^C NMR of 2,5-diketopiperazine: Product **20** in CDCl_3_ 38](#_Toc181632670)

[^1^H NMR of 2,5-diketopiperazine: Product **21** in CDCl_3_ 39](#_Toc181632671)

[^1^H NMR of Cbz-l-Pro-(2*S*)-Ind-OH (**22**) in CDCl_3_ 40](#_Toc181632672)

[DEPT ^13^C NMR of Cbz-l-Pro-(2*S*)-Ind-OH (**22**) in CDCl_3_ 41](#_Toc181632673)

[^1^H NMR of Cbz-l-Pro-(2*S*)-Ind-l-Pro-OMe (**23**) in CDCl_3_ 42](#_Toc181632674)

[DEPT ^13^C NMR of Cbz-l-Pro-(2*S*)-Ind-l-Pro-OMe (**23**) in CDCl_3_ 43](#_Toc181632675)

[^1^H NMR of HN-l-Pro-(2*S*)-Ind-l-Pro-OMe (**24**) in CDCl_3_ 44](#_Toc181632676)

[DEPT ^13^C NMR of HN-l-Pro-(2*S*)-Ind-l-Pro-OMe (**24**) in CDCl_3_ 45](#_Toc181632677)

[5 HPLC-DAD and Mass spectra 46](#_Toc181632678)

[HPLC-MS of Ac-(2*S*)-Ind-l-Ala-OMe (**6**) 46](#_Toc181632679)

[HPLC-MS of Boc-(2*S*)-Ind-l-Ala-OMe (**7**) 47](#_Toc181632680)

[HPLC-MS of Ac-(2*S*)-Ind-d-Ala-OMe (**8**) 48](#_Toc181632681)

[HPLC-MS of Boc-(2*S*)-Ind-d-Ala-OMe (**9**) 49](#_Toc181632682)

[HPLC-MS of Boc-Gly-(2*S*)-Ind-OMe (**13**) 50](#_Toc181632683)

[HPLC-MS of Cbz-Gly-(2*S*)-Ind-OMe (**14**) 51](#_Toc181632684)

## Peptide coupling reaction between (2*S*)-Ind-OMe and PG-Ala-OH

TABLE S1: Synthesis of H-(2*S*)-Ind-OH containing dipeptides.

| ***PG-Ala-OH***  (equiv.) | ***Base***  (equiv.) | ***Coupling reag.***  (equiv.) | ***Solvent*** | ***T*** (°C) | ***Yield***  (%) |
| --- | --- | --- | --- | --- | --- |
| Boc-l-Ala-OH (1) | TEA (2.8) | Mukaiy. (1.4) | DCM | reflux | **/** |
| Boc- l-Ala-OH (1) | TEA (2.8) | Mukaiy. (1.4) | DMF | 80 | **/** |
| Cbz- l-Ala-OH (1.1) | TEA (2.8) | Mukaiy. (1.4) | DMF | 80 | **/** |
| Boc- d-Ala-OH (1.1) | TEA (2.8) | Mukaiy. (1.4) | DCM | reflux | **/** |
| Boc- l-Ala-OH (1.1) | DBU (2.8) | Mukaiy. (1.4) | DMF | 80 | **/** |
| Cbz- l-Ala-OH (1.1) | DBU (2.8) | Mukaiy. (1.4) | DMF | 80 | **/** |
| Cbz- l-Ala-OH (1.1) | TEA (2.8) | Mukaiy. (1.4) | DMF | MW^a^ | **/** |
| Cbz- l-Ala-OH (1.2) | TEA (3.1) | SOCl_2_ (1.2) | DCM | rt | **/** |
| Boc- l-Ala-OH (1) | DIPEA (2) | PyBOP (1.1) | DCM | rt | **/** |

^a^ Reaction performed in microwave at 100 °C, 150 W, 45 min.

## Synthesis

# General method A

### Ac-(2*S*)-Ind-l-Ala-OMe (**6**)

Following general method A, 100 mg of Ac-(*2S*)-Ind-OH (0.49 mmol) and 203 mg HBTU (0.54 mmol), were let stirring in 6 mL of dry MeCN for 15 minutes at room temperature. A mixture containing 68 mg of l-Ala-OMe۰HCl (0.49 mmol) and 0.21 mL of TEA (1,51 mmol) was added dropwise. The reaction mixture was stirred at room temperature, for 16 h. The solvent was removed under reduced pressure and the residue was re-dissolved in DCM and washed with 1 M HCl, a saturated solution of NaHCO_3_ and brine. The organic phase was dried over Na_2_SO_4_ and filtered. The solvent was removed under reduced pressure. The crude product was purified by flash chromatography, using DCM/MeOH 97:3 as eluent mixture, to afford the pure product **6** (92 mg, 65 %) as a white solid. MW = 290.32 g/mol; ^1^H NMR (500 MHz, CDCl_3_) mixture of rotamers δ 8.44 – 6.91 (m, 4H), 5.49 – 4.30 (m, 2H), 3.73 (s, 5H), 2.61 – 2.16 (m, 3H), 1.33 (d, J = 6.9 Hz, 3H); ^1^H NMR (400 MHz, DMSO-*D*6) prevalence of one specific rotamers δ 8.73 (d, *J* = 7.1 Hz, 1H), 7.99 (d, *J* = 8.0 Hz, 1H), 7.32 – 7.02 (m, 2H), 6.93 (td, *J* = 7.4, 1.1 Hz, 1H), 4.97 (dd, *J* = 11.1, 3.3 Hz, 1H), 4.27 (p, *J* = 7.2 Hz, 1H), 3.57 (s, 5H), 3.52 (d, *J* = 4.9 Hz, 1H), 3.00 (dd, *J* = 16.8, 3.2 Hz, 1H), 1.97 (s, 3H). ^13^C NMR (100 MHz, CDCl_3_) δ 8 170.0, 127.4, 126.0, 124.5, 117.8, 115.1, 63.1, 61.8, 52.6, 48.2, 34.5, 29.8, 24.0; HRMS (TOF MS ES+): expected m/z for C_15_H_19_N_2_O_4_ [M+H]^+^ 291.1339; found 291.1350 (3.74 ppm).

### Boc-(2*S*)-Ind-l-Ala-OMe (**7**)

Following general method A, 100 mg of Boc-(*2S*)-Ind-OH (0.38 mmol) and 159 mg of HBTU (0,43 mmol), were let stirring in 5 mL of dry MeCN for 15 minutes at room temperature. A mixture containing 53 mg of l-Ala-OMe۰HCl (0.38 mmol) and 0,16 mL of TEA (1,18 mmol) was added dropwise. The reaction mixture was stirred at room temperature, for 16 h. The solvent was removed under reduced pressure and the residue was re-dissolved in DCM and washed with 1 M HCl, a saturated solution of NaHCO_3_ and brine. The organic phase was dried over Na_2_SO_4_ and filtered. The solvent was removed under reduced pressure. The crude product was purified by flash chromatography, using n-hexane/EtOAc 60:40 as eluent mixture, to afford the pure product **7** (61 mg, 46 %) as white solid. MW = 348.40 g/mol; Rf = 0.33 (n-hexane/EtOAc 60:40); ^1^H NMR (400 MHz, CDCl_3_) δ 7.69 (s, 1H), 7.23 – 7.09 (m, 2H), 6.97 (td, *J* = 7.5, 1.0 Hz, 1H), 4.85 (dd, *J* = 10.8, 4.3 Hz, 1H), 4.56 (p, *J* = 7.2 Hz, 1H), 3.73 (s, 3H), 3.61 – 3.13 (m, 2H), 1.55 (s, 9H), 1.35 (d, *J* = 7.2 Hz, 3H); ^13^C NMR (100 MHz, CDCl_3_) δ 173.1, 171.2, 127.7, 124.9, 123.3, 116.4, 115.6, 82.5, 61.8, 52.6, 48.1, 28.3, 18.5; HRMS (TOF MS ES+): expected m/z for C_18_H_25_N_2_O_5_ [M+H]^+^ 349.1758; found 349.1762 (3.48 ppm).

### Ac-(2*S*)-Ind-d-Ala-OMe (**8**)

Following general method A, 57 mg of Ac-(*2S*)-Ind-OH (0.28 mmol) and 116 mg of HBTU (0.30 mmol), were let stirring in 4 mL of dry MeCN for 15 minutes at room temperature. A mixture containing 39 mg of d-Ala-OMe۰HCl (0.28 mmol) and 0,12 mL of TEA (0.86 mmol) was added dropwise. The reaction mixture was stirred at room temperature, for 16 h. The solvent was removed under reduced pressure and the residue was re-dissolved in DCM and washed with 1 M HCl, a saturated solution of NaHCO_3_ and brine. The organic phase was dried over Na_2_SO_4_ and filtered. The solvent was removed under reduced pressure. The crude product was purified by flash chromatography, using DCM/MeOH 97:3 as eluent mixture, to afford the pure product **8** (64 mg, 79 %) as a white solid. MW = 290.32 g/mol; ^1^H NMR (500 MHz, CDCl_3_) δ 8.34 – 6.92 (m, 4H), 5.34 – 4.37 (m, 2H), 3.66 (s, 5H), 2.60 – 2.07 (m, 3H), 1.40 (d, J =7.1 Hz, 3H); ^1^H NMR (400 MHz, DMSO-*D*6) prevalence of one specific rotamers δ 8.77 (d, *J* = 6.8 Hz, 1H), 7.99 (d, *J* = 8.1 Hz, 1H), 7.19 – 7.04 (m, 2H), 6.93 (td, *J* = 7.4, 1.1 Hz, 1H), 4.95 (dd, *J* = 11.1, 3.3 Hz, 1H), 4.19 (p, *J* = 7.2 Hz, 1H), 3.60 – 3.48 (m, 5H), 2.96 (dd, *J* = 16.9, 3.2 Hz, 1H), 2.01 (s, 3H). ^13^C NMR (100 MHz, CDCl_3_) δ 169.9, 127.5, 125.8, 124.6, 115.3, 63.3, 61.7, 52.6, 48.3, 34.4, 29.8, 23.9; HRMS (TOF MS ES+): expected m/z for C_15_H_19_N_2_O_4_ [M+H]^+^ 291.1339; found 291.1351 (4.15 ppm).

### Boc-(2*S*)-Ind-d-Ala-OMe (**9**)

Following general method A, 100 mg of Boc-(*2S*)-Ind-OH (0.38 mmol) and 159 mg of HBTU (0,43 mmol), were let stirring in 5 mL of dry MeCN for 15 minutes at room temperature. A mixture containing 53 mg of d-Ala-OMe۰HCl (0.38 mmol) and 0,16 mL of TEA (1,18 mmol) was added dropwise. The reaction mixture was stirred at room temperature, for 16 h. The solvent was removed under reduced pressure and the residue was re-dissolved in DCM and washed with 1 M HCl, a saturated solution of NaHCO_3_ and brine. The organic phase was dried over Na_2_SO_4_ and filtered. The solvent was removed under reduced pressure. The crude product was purified by flash chromatography, using n-hexane/EtOAc 60:40 as eluent mixture, to afford the pure product **9** (108 mg, 82%) as a white solid. MW = 348.40 g/mol; ^1^H NMR (400 MHz, CDCl_3_*)* δ 7.70 (s, 1H), 7.23 – 7.08 (m, 2H), 6.98 (td, *J* = 7.4, 1.1 Hz, 1H), 4.92 – 4.78 (m, 1H), 4.56 (p, *J* = 7.2 Hz, 1H), 3.68 (s, 3H), 3.56 – 3.18 (m, 2H), 1.55 (s, 9H), 1.40 (d, *J* = 7.2 Hz, 3H); NMR (100 MHz, CDCl_3_) δ 173.1, 127.8, 124.9, 123.4, 115.5, 82.4, 61.9, 52.6, 48.1, 28.4, 18.5; HRMS (TOF MS ES+): expected m/z for C_18_H_25_N_2_O_5_ [M+H]^+^ 349.1758; found 349.1763 (3.76 ppm).

# General method B

### Boc-l-Ala-(2*S*)-Ind-OMe (**10**)

Following general method B, 129 mg of Boc-l-Ala-OH (0.68 mmol), were dissolved in 3 mL of dry DCM. The solution was cooled at 0 °C and 0.19 mL of DIPEA (1.12 mmol) and 201 mg of BOP-Cl (0.79 mmol) were added. The mixture was warmed at room temperature for 30 minutes and then cooled again at 0 °C, to add 100 mg (0.56 mmol) of **1**, 0.10 mL of DIPEA (0.56 mmol), and 3 mL of dry DCM. The reaction mixture was warmed to room temperature and stirred under reflux for 22 h. The reaction was cooled down at room temperature and after dilution with DCM, it was washed with H_2_O and brine. The organic phase was dried over Na_2_SO_4_, and the solvent was removed under reduced pressure. The crude product was purified by flash chromatography using Cyclohexane/EtOAc 75:25 as eluent mixture, to afford the pure product **10** (34 mg, 17%) as a white solid. MW = 348.40 g/mol; Rf = 0.43 (Cyclohexane/EtOAc 70:30); ^1^H NMR (400 MHz, CDCl_3_) δ 8.29 – 6.97 (m, 4H), 5.64 – 4.81 (m, 2H), 3.81 – 3.70 (m, 3H), 3.69 – 3.03 (m, 2H), 1.56 – 1.34 (m, 12H); ^13^C NMR (100 MHz, CDCl_3_) δ 171.9, 171.8, 171.6, 171.5, 155.2, 154.9, 142.4, 140.0, 131.1, 128.8, 128.4, 128.1, 125.9, 125.6, 124.9, 124.7, 124.5, 124.2, 117.6, 116.5, 114.2, 79.9, 79, 63.6, 60.5, 60.3, 53.3, 53.2, 52.6, 52.3, 48.7, 48.5, 33.6, 28.4, 19.9, 18.9.

### Boc-d-Ala-(2*S*)-Ind-OMe (**11**)

Following general method B, 129 mg of Boc-d-Ala-OH (0.68 mmol), were dissolved in 3 mL of dry DCM. The solution was cooled at 0 °C and 0.19 mL of DIPEA (1.12 mmol) and 201 mg of BOP-Cl (0.79 mmol) were added. The mixture was warmed at room temperature for 30 minutes and then cooled again at 0 °C, to add 100 mg (0.56 mmol) of **1** and 0.10 mL of DIPEA (0.56 mmol), and 3 mL of dry DCM. The reaction mixture was warmed to room temperature and stirred under reflux for 22 h. The reaction was cooled down at room temperature and after dilution with DCM, it was washed with H_2_O and brine. The organic phase was dried over Na_2_SO_4_, and the solvent was removed under reduced pressure. The crude product was purified by flash chromatography using Cyclohexane/EtOAc 75:25 as eluent mixture, to afford the pure product **11** (21 mg, 11%) as white solid. MW = 348.40 g/mol; Rf = 0.41 (Cyclohexane/EtOAc 70:30); ^1^H NMR (400 MHz, CDCl_3_) δ 8.32 – 6.94 (m, 4H), 5.76 – 4.89 (m, 2H), 3.87 – 3.68 (m, 3H), 3.68 – 2.92 (m, 2H), 1.60 – 1.05 (m, 12H). ^13^C NMR (100 MHz, CDCl_3_) δ 172.4, 172.2, 155.4, 142.3, 129.8, 129.6, 128.0, 127.9, 125.1, 124.6, 124.5, 118.0, 117.5, 116.53, 115.9, 80.2, 61.1, 60.6, 53.0, 48.2, 33.4, 28.5, 18.9, 18.3.

# General method C

### Cbz-((2*S*)-Ind)_2_-O*t*Bu (**12**)

Following general method C, 149 mg of Cbz-(2*S*)-Ind-OH (0.50 mmol) were dissolved in 7 mL of dry DCM. 164 mg of Mukaiyama reagent (0.64 mmol were added and 0.18 mL of TEA (1.28 mmol) were dropped slowly into the stirred solution. 100 mg of H-(2*S*)-Ind-OtBu (0.46 mmol) were added. The reaction mixture was stirred under reflux for 5 h. The mixture was cooled down at room temperature and after dilution with DCM, it was washed with 1M HCl, a saturated solution of NaHCO_3_ and brine. The organic phase was dried over Na_2_SO_4,_ filtered, and the solvent was removed under reduced pressure. The crude product was purified by flash chromatography using Cyclohexane/EtOAc 75:25 as eluent mixture, to afford the pure product **12** (147 mg, 65 %) as a white solid. MW = 498.58 g/mol; Rf = 0.43 (Cyclohexane/EtOAc 75:25); ^1^H NMR (400 MHz, CDCl_3_) δ 8.32 – 6.83 (m, 13H), 5.75 – 4.53 (m, 4H), 3.89 – 2.78 (m, 4H), 1.76 – 1.33 (m, 9H); ^13^C NMR (100 MHz, CDCl_3_) δ 127.9, 127.5, 126.0, 124.5, 122.9, 114.9, 114.7, 113.3, 67.1, 61.0, 59.3, 33.1, 31.4, 27.9; HRMS (TOF MS ES+): m/z calcd for C_30_H_31_N_2_O_5_ [M+H]^+^: 499.2227; found: 499.2218 (-1.84 ppm).

### Boc-d-Pro-(2*S*)-Ind-OMe (**16**)

Following general method C, 130 mg of Boc-d-Pro-OH (0.62 mmol), were dissolved in 9 mL of dry DCM. 200 mg of Mukaiyama reagent (0.79 mmol) were added and 0.22 mL of TEA (1.58 mmol) were dropped slowly into the stirred solution. 100 mg of **1** (0.56 mmol) were added. The reaction mixture was stirred under reflux for 24 h. The mixture was cooled down at room temperature and after dilution with DCM, it was washed with 1M HCl, a saturated solution of NaHCO_3_ and brine. The organic phase was dried over Na_2_SO_4,_ filtered, and the solvent was removed under reduced pressure. The crude product was purified by flash chromatography using n-hexane/EtOAc 60:40 as eluent mixture, to afford the pure product **16** (55 mg, 26 %) as a pale-yellow solid. MW = 374.44 g/mol; Rf = 0.32 (n-hexane/EtOAc 60:40); ^1^H NMR (500 MHz, CDCl_3_) δ 8.30 – 6.92 (m, 4H), 5.74 – 5.16 (m, 1H), 4.37 – 4.17 (m, 1H), 3.80 – 3.21 (m, 7H), 2.39 – 1.75 (m, 4H), 1.47 – 1.27 (m, 9H)< ^13^C NMR (100 MHz, CDCl_3_) δ 172.6, 172.6, 172.5, 171.9, 154.7, 153.4, 142.8, 142.7, 129.3, 128.23, 128.2, 127.8, 124.3, 124.3, 124.2, 117.9, 117.8, 80.2, 79.8, 60.6, 60.5, 58.1, 58.1, 53.0, 52.9, 33.8, 33.7, 28.6, 28.2.

### Cbz-l-Pro-(2*S*)-Ind-OMe (**17**)

Following general method C, 154 mg of Cbz-l-Pro-OH (0.62 mmol) were dissolved in 9 mL of dry DCM. 200 mg of Mukaiyama reagent (0.79 mmol) were added and 0.22 mL of TEA (1.58 mmol) were dropped slowly into the stirred solution. 100 mg of **1** (0.56 mmol) were added. The reaction mixture was stirred at reflux for 22 h. The mixture was cooled down at room temperature and after dilution with DCM, it was washed with 1M HCl, a saturated solution of NaHCO_3_ and brine. The organic phase was dried over Na_2_SO_4,_ filtered, and the solvent was removed under reduced pressure. The crude product was purified by flash chromatography using Cyclohexane/EtOAc 50:50 as eluent mixture, to afford the pure product **17** (149 mg, 65 %) as a pale-yellow solid. MW = 408.45 g/mol; Rf = 0.26 (n-hexane/EtOAc 60:40); ^1^H NMR (400 MHz, CDCl_3_) δ 8.32 – 6.94 (m, 9H), 5.35 – 4.35 (m, 4H), 3.89 – 3.41 (m, 5H), 3.38 – 2.90 (m, 1H), 2.57 – 1.81 (m, 4H).^13^C NMR (100 MHz, CDCl_3_) δ 171.9, 171.7, 140.6, 140.5, 136.9, 136.8, 131.4, 131.3, 128.5, 128.3, 128.1, 128.0, 127.9, 127.8, 127.4, 125.9, 124.4, 123.9, 123.8, 117.8, 114.1, 114.0, 67.1, 66.8, 65.9, 60.5, 60.4, 59.4, 58.3, 52.6, 52.5, 47.5, 46.9, 31.5, 31.4; HRMS (TOF MS ES+): expected m/z for C_23_H_25_N_2_O_5_ [M+H]^+^: 409.1758; found: 409.1753 (-1.31 ppm).

### Cbz-l-Pro-(2*S*)-Ind-O*t*Bu (**18**)

Following general method C, 125 mg of Cbz-l-Pro-OH (0.50 mmol) were dissolved in 7 mL of dry DCM. 164 mg of Mukaiyama reagent (0.64 mmol) were added and 0.18 mL of TEA (1.28 mmol) were dropped slowly into the stirred solution.100 mg of (2*S*)-Ind-O*t*Bu (0.46 mmol) were added. The reaction mixture was stirred at reflux for 23 h. The mixture was cooled down at room temperature and after dilution with DCM, it was washed with 1M HCl, a saturated solution of NaHCO_3_ and brine. The organic phase was dried over Na_2_SO_4,_ filtered, and the solvent was removed under reduced pressure. The crude product was purified by flash chromatography, using Cyclohexane/EtOAc 65:35 as eluent mixture to afford the pure product **18** (103 mg, 50 %) as a pale brown solid. MW = 450.53 g/mol; Rf = 0.46 (n-hexane/EtOAc 60:40); ^1^H NMR (400 MHz, CDCl_3_) δ 8.36 – 6.92 (m, 9H), 5.48 – 4.28 (m, 4H), 3.97 – 2.82 (m, 4H), 2.61 – 1.85 (m, 4H), 1.52 – 1.34 (m, 8H). ^13^C (100 MHz CDCl_3_) δ 128.4, 128.2, 127.9, 127.9, 127.7, 127.7, 127.6, 127.2, 125.8, 123.6, 123.5, 61.1, 61.0, 59.21, 58.3, 27.9; HRMS (TOF MS ES+): expected m/z for C_26_H_31_N_2_O_5_ [M+H]^+^: 451.2227; found: 451.2222 (-1.29 ppm).

# General method D

### Boc-((2*S*)-Ind)_2_-OMe (**5**)

Following general method D, 100 mg of **1** (0.56 mmol) and 0.39 ml of DIPEA (2.26 mmol) were added to a solution of148 mg of Boc-(2*S*)-Ind-OH (0.56 mmol) in 5 mL of dry EtOAc. Lastly, 0.67 mL of T3P^®^ 50% wt/wt EtOAc solution (1.13 mmol) were added dropwise. The reaction mixture was stirred overnight under reflux. The reaction was quenched by the addition of H_2_O and extracted with EtOAc. The organic phase was washed with brine, dried over Na_2_SO_4_, and filtered. The solvent was removed under reduced pressure. The crude product was purified by flash chromatography, using n-hexane/EtOAc 70:30 as eluent mixture to afford the pure product **5** (95 mg, 40 %) as a white solid. MW = 422.48 g/mol; Rf = 0.34 (n-hexane/EtOAc 70:30); ^1^H NMR (400 MHz, CDCl_3_) δ 8.31 – 6.78 (m, 9H), 5.66 – 4.82 (m, 2H), 3.91 – 2.91 (m, 8H), 1.58 (s, 9H); ^13^C NMR (101 MHz, CDCl_3_) δ 171.8, 171.5, 169.8, 169.2, 164.4, 152.8, 151.5, 143.3, 143.0, 142.3, 140.6, 131.3, 131.1, 129.8, 128.5, 128.4, 128.1, 128.0, 127.5, 126.2, 126.1, 125.1, 124.4, 124.1, 123.9, 122.6, 122.5, 117.8, 115.9, 114.9, 114.7, 114.6, 113.5, 113.4, 110.6, 82.3, 81.0, 61.8, 60.4, 60.0, 59.6, 53.1, 52.7, 52.5, 28.5, 28.2.

### Boc-Gly-(2*S*)-Ind-OMe (**13**)

Following general method D, 100 mg of **1** (0.56 mmol) and 0.39 ml of DIPEA (2.26 mmol) were added to a solution of 98 mg of Boc-Gly-OH (0. 56 mmol) in 5 mL of dry EtOAc. Lastly, 0.67 mL of T3P^®^ 50% wt/wt EtOAc solution (1.13 mmol) were added dropwise. The reaction mixture was stirred overnight at room temperature. The reaction was quenched by the addition of H_2_O and extracted with EtOAc. The organic phase was washed with brine, dried over Na_2_SO_4_, and filtered. The solvent was removed under reduced pressure. The crude product was purified by flash chromatography, using n-hexane/EtOAc 60:40 as eluent mixture to afford the pure product **13** (153 mg, 82 %) as a white solid. MW = 334.37 g/mol; Rf = 0.40 (n-hexane/EtOAc 60:40); ^1^H NMR (400 MHz, CDCl_3_) δ 8.25 – 7.09 (m, 3H), 7.05 (t, *J* = 7.4 Hz, 1H), 5.69 – 5.35 (m, 1H), 5.30 – 4.78 (m, 1H), 4.61 – 2.94 (m, 7H), 1.44 (s, 9H);^13^C (100 MHz, CDCl_3_) δ 171.6, 171.4, 156.0, 151.8, 12 8.1, 124.5, 117.5, 84.9, 79.9, 79.7, 77.4, 77.1, 76.9, 60.5, 60.0, 53.3, 46.9, 46.5, 33.8, 27.9; HRMS (TOF MS ES+): expected m/z C_17_H_23_N_2_O_5_ for [M+H]^+^: 335.1601; found: 335.1609 (4.01 ppm).

### Cbz-Gly-(2*S*)-Ind-OMe (**14**)

Following general method D, 100 mg of **1** (0.56 mmol) and 0.39 ml of DIPEA (2.26 mmol) were added to a solution of 118 mg of Cbz-Gly-OH (0. 56 mmol) in 5 mL of dry EtOAc Lastly, 0.67 mL of T3P^®^ 50% wt/wt EtOAc solution (1.13 mmol) were added dropwise. The reaction mixture was stirred overnight at room temperature. The reaction was quenched by the addition of H_2_O and extracted with EtOAc. The organic phase was washed with brine and dried over Na_2_SO_4,_ and the solvent was removed under reduced pressure. The crude product was purified by flash chromatography, using n-hexane/EtOAc 55:45 as eluent mixture to afford the pure product **14** (144 mg, 70 %) as a white solid. MW = 368.39 g/mol; Rf = 0.27 (n-hexane/EtOAc 60:40); ^1^H NMR (400 MHz, CDCl_3_) δ 8.28 – 7.27 (m, 5H), 7.28 – 7.10 (m, 3H), 7.10 – 7.02 (m, 1H), 5.90 – 5.64 (m, 1H), 5.27 – 4.83 (m, 3H), 4.58 – 3.01 (m, 7H); ^13^C NMR (101 MHz, CDCl_3_) δ 171.26, 166.86, 156.35, 142.15, 136.43, 130.87, 128.64, 128.26, 128.16, 126.01, 124.81, 124.51, 124.36, 117.49, 114.13, 67.15, 60.37, 59.85, 53.36, 52.78, 45.48, 44.00, 33.78; HRMS (TOF MS ES+): expected m/z C_20_H_20_N_2_O_5_Na for [M+Na]^+^: 391.1264; found: 391.1276 (3.12 ppm).

### Boc-l-Pro-(2*S*)-Ind-OMe (**15**)

Following general method D, 100 mg of **1** (0.56 mmol) and 0.39 ml of DIPEA (2.26 mmol) were added to a solution of 121 mg of Boc-l-Pro-OH (0.56 mmol) in 5 mL of dry EtOAc. Lastly, 0.67 mL of T3P^®^ 50% wt/wt EtOAc solution (1.13 mmol) were added dropwise. The reaction mixture was stirred overnight at reflux. The reaction was quenched by the addition of H_2_O and extracted with EtOAc. The organic phase was washed with brine and dried over Na_2_SO_4,_ and the solvent was removed under reduced pressure. The crude product was purified by flash chromatography, using n-hexane/EtOAc 55:45 as eluent mixture to afford the pure product **15** (115 mg, 55 %) as white solid. MW = 374.44 g/mol; Rf = 0.40 (n-hexane/EtOAc 55:45);  ^1^H NMR (400 MHz, CDC_l3_) δ 8.35 – 6.83 (m, 4H), 5.34 – 5.16 (m, 1H), 5.08 – 4.80 (m, 1H), 3.86 – 2.97 (m, 7H), 2.50 – 1.81 (m, 4H), 1.44 (s, 9H).; ^13^C (100 MHz, CDCl_3_) δ 171.9, 170.8, 154.5, 153.8, 142.5, 140.6, 131.2, 131.1, 128.0, 127.9, 117.8, 114.0, 110.6, 79.8, 79.6, 61.0, 60.4, 60.3, 58.9, 58.4, 52.6, 52.5, 46.9, 46.8, 34.5, 28.6, 28.3.

# 2,5-diketopiperazine formation

### Diketopiperazine **19**

Following general method E for Cbz protecting group removal, 80 mg of **12** (0.16 mmol) were dissolved in 4 mL of dry MeOH and 10 % wt Pd/C was added. The mixture was stirred overnight at room temperature under hydrogen atmosphere. The reaction mixture was filtered on a celite pad, and the residue was washed with MeOH. The solvent was removed under reduced pressure to afford the 2,5-diketopiperazine **19** (48 mg, quantitative) as a white solid without further purification. MW = 290.32 g/mol; ^1^H NMR (400 MHz, CDCl_3_) δ 8.14 (d, J = 8.0 Hz, 2H), 7.34 – 7.25 (m, 6H), 7.14 (td, J = 7.5, 1.1 Hz, 2H), 5.02 (dd, J = 10.4, 8.8 Hz, 2H), 3.85 (dd, J = 16.8, 8.7 Hz, 2H), 3.57 – 3.41 (m, 2H). The complete characterization of **19** is reported in the literature.^1^

### Diketopiperazine **20**

Following general method E for Boc protecting group removal, 60 mg of **13** (0,18 mmol) were dissolved in 1 mL of dry DCM. 0.11 mL of TFA (1.44 mmol) were added dropwise and the mixture was stirred at room temperature for 5 h. The solvent was removed under reduced pressure and the residue was dissolved in DCM and washed with a saturated solution of NaHCO_3_ and brine. organic phase was dried over Na_2_SO_4,_ and the solvent was removed under reduced pressure to afford the 2,5-diketopiperazine **20** (25 mg, 70 %) as a white solid without further purification. MW = 202.21 g/mol; ^1^H NMR (400 MHz, CDCl_3_) δ 8.08 (dd, J = 7.8, 1.2 Hz, 1H), 7.29 – 7.20 (m, 3H), 7.11 (td, J = 7.5, 1.1 Hz, 1H), 6.42 (s, 1H), 4.80 (td, J = 10.1, 1.3 Hz, 1H), 4.24 (dd, J = 16.7, 1.5 Hz, 1H), 4.08 – 3.95 (m, 1H), 3.58 (dd, J = 16.6, 9.8 Hz, 1H), 3.39 (dd, J = 16.6, 10.1 Hz, 1H); ^13^C NMR (100 MHz, CDCl_3_) δ 169.6, 162.5, 140.9, 129.3, 128.0, 125.2, 125.0, 116.1, 59.6, 47.3, 31.1.

### Diketopiperazine **21**

Following general method E for Cbz protecting group removal, 100 mg of **17** (0.24 mmol) were dissolved in 5 mL of dry MeOH and 10 % wt Pd/C was added. The mixture was stirred overnight at room temperature under hydrogen atmosphere. The reaction mixture was filtered on a celite pad and the residue was washed with MeOH. The solvent was removed under reduced pressure to afford the 2,5-diketopiperazine **21** (53 mg, 91 %) as a white solid without further purification MW = 242.28 g/mol; ^1^H NMR (400 MHz, CDCl_3_) δ 8.10 (dd, J = 7.8, 1.3 Hz, 1H), 7.32 – 7.21 (m, 3H), 7.11 (td, J = 7.5, 1.1 Hz, 1H), 4.88 (ddd, J = 10.4, 9.3, 1.2 Hz, 1H), 4.39 – 4.30 (m, 1H), 3.72 (DDT, J = 16.8, 9.2, 1.2 Hz, 1H), 3.64 (dd, J = 8.3, 5.6 Hz, 2H), 3.39 (dd, J = 16.7, 10.5 Hz, 1H), 2.52 – 2.29 (m, 2H), 2.17 – 1.91 (m, 2H). The complete characterization of **21** is reported in the literature.^1^

# Synthesis of Cbz-l-Pro-(2*S*)-Ind-l-Pro-OMe **(23)** and the *N*-deprotected product **(24)**

### Cbz-l-Pro-(2*S*)-Ind-l-Pro-OMe (**23**)

630 mg of **17** (1.54 mmol) were dissolved in 20 mL of a solution of THF/H_2_O/MeOH 4:1:1. 190 mg of LiOH۰H_2_O (4.62 mmol) were added, and the reaction mixture was stirred at room temperature for 5 h. The mixture was acidified with 1 M HCl and extracted with EtOAc. The organic phase was dried over Na_2_SO_4_, and the solvent was removed under reduced pressure to recover the crude intermediate **22** (485 mg, 80 %) as a pale brown solid, without further purification. MW = 394.43 g/mol; ^1^H NMR (400 MHz, CDCl_3_) δ 8.41 – 6.85 (m, 9H), 5.36 – 4.22 (m, 4H), 3.99 – 2.74 (m, 4H), 2.57 – 1.80 (m, 4H).; ^13^C NMR (101 MHz, CDCl3) δ 128.5, 128.3, 127.9, 127.8, 127.8, 127.4, 126.1, 124.5, 124.3, 61.0, 58.9, 57.9.

Afterwards, 320 mg (0.81 mmol) of **22** was dissolved in 15 mL of dry MeCN and 340 mg of HATU (0.89 mmol) were added. The mixture was stirred for 15 min at room temperature and a solution of 105 mg of l-Pro-OMe (0.81 mmol) in 5 mL of dry MeCN was added. 0.35 mL of TEA (2.5 mmol) were added dropwise. The reaction mixture was stirred at room temperature for 6 h, then diluted with DCM and washed with 1 M HCl, a saturated solution of NaHCO_3_ and brine. The organic phase was dried over Na_2_SO_4,_ and the solvent was removed under reduced pressure. The crude product was purified by flash chromatography, using EtOAc/n-hexane (90:10) as eluent mixture, to afford the pure product **23** (288 mg, 71 %) as a white solid. MW = 505.57 g/mol; Rf = 0.38 (EtOAc/n-hexane 90:10); ^1^H NMR (400 MHz, CDCl_3_) δ 7.45 – 6.92 (m, 9H), 5.45 – 4.92 (m, 4H), 4.66 – 4.48 (m, 1H), 3.93 – 3.45 (m, 7H), 3.31 – 2.95 (m, 1H), 2.50 – 1.68 (m, 8H); ^13^C NMR (100 MHz, CDCl3) δ 128.4, 128.2, 127.9, 127.8, 127.7, 127.7, 127.6, 127.5, 126.0, 123.6, 123.5, 113.7, 113.7, 59.5, 59.5, 59.4, 58.5, 58.5, 58.4, 52.2; HRMS (TOF MS ES+): expected m/z for C_28_H_32_N_3_O_6_ [M+H]^+^: 506.2286; found: 506.2279 (-1.24 ppm).

### HN-l-Pro-(2*S*)-Ind-l-Pro-OMe (**24**)

30 mg of **23** (0.06 mmol) were dissolved in 1 mL of dry MeOH and 10% wt Pd/C was added. The mixture was stirred at room temperature under hydrogen atmosphere for 6 h. The reaction mixture was filtered on a celite pad and the residue was washed with MeOH. The solvent was removed under reduced pressure to afford **24** (17 mg, 76 %), as a white solid, without further purification MW = 371. 44 g/mol; ^1^H NMR (400 MHz, CDCl_3_) δ 8.59 – 6.74 (m, 4H), 5.67 – 2.73 (m, 11H), 2.71 – 1.50 (m, 9H).^13^C NMR (100 MHz, CDCl3) δ 127.8, 125.9, 125.0, 124.9, 123.5, 115.8, 113.7, 61.4, 60.9, 59.9, 59.6, 58.5, 52.2; HRMS (TOF MS ES+): expected m/z for C_20_H_26_N_3_O_4_ [M+H]^+^: 372.1918; found: 372.1912 (-1.67 ppm).

## Reference

1. Jainta, M., Nieger, M. & Bräse, S. Microwave-assisted stereoselective one-pot synthesis of symmetrical and unsymmetrical 2,5-diketopiperazines from unprotected amino acids. *European J. Org. Chem.* 5418–5424 (2008)

## NMR spectra

### ^1^H NMR of Ac-(2*S*)-Ind-l-Ala-OMe (**6**) in CDCl_3_


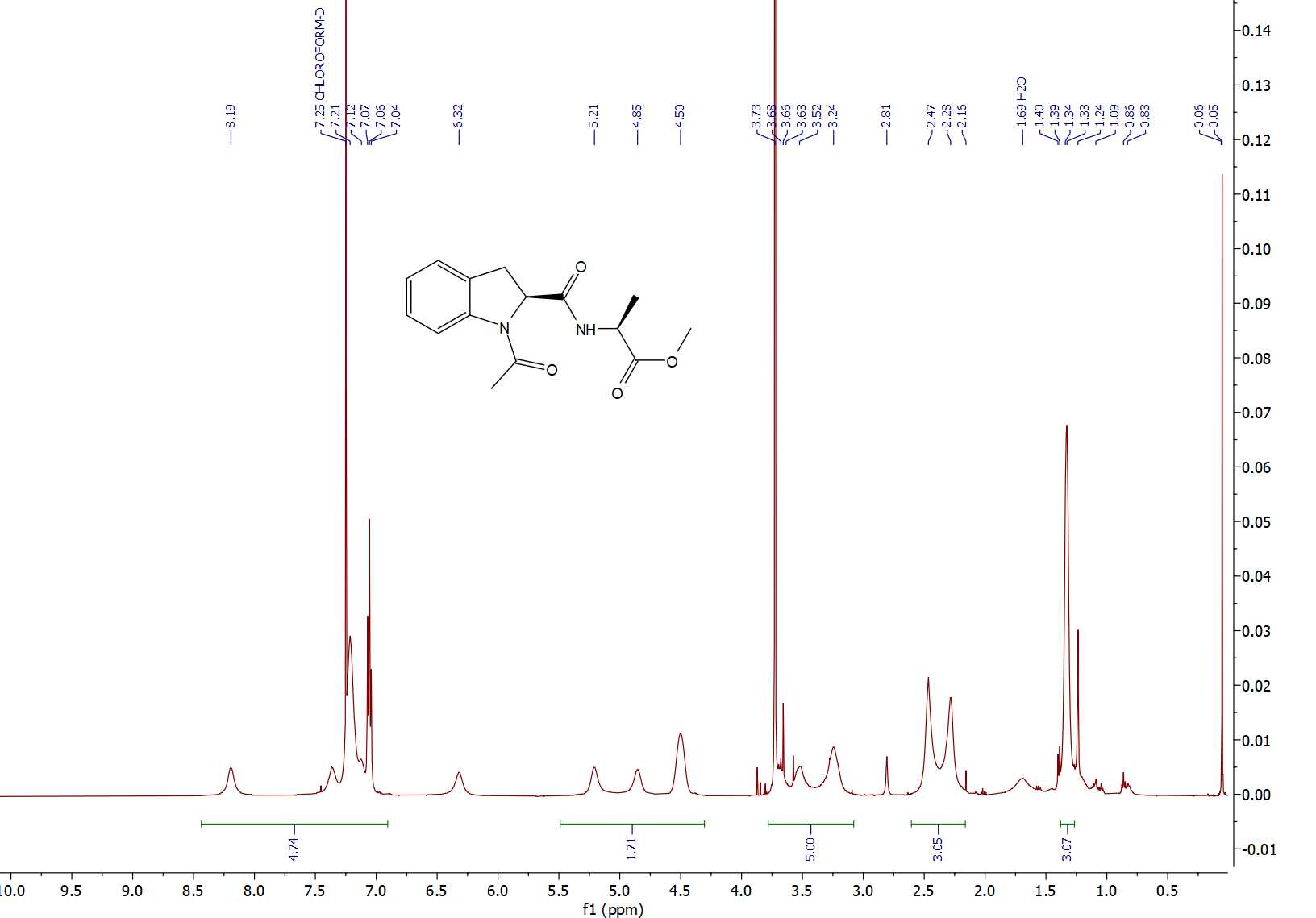


### ^13^C NMR of Ac-(2*S*)-Ind-l-Ala-OMe (**6**) in CDCl_3_


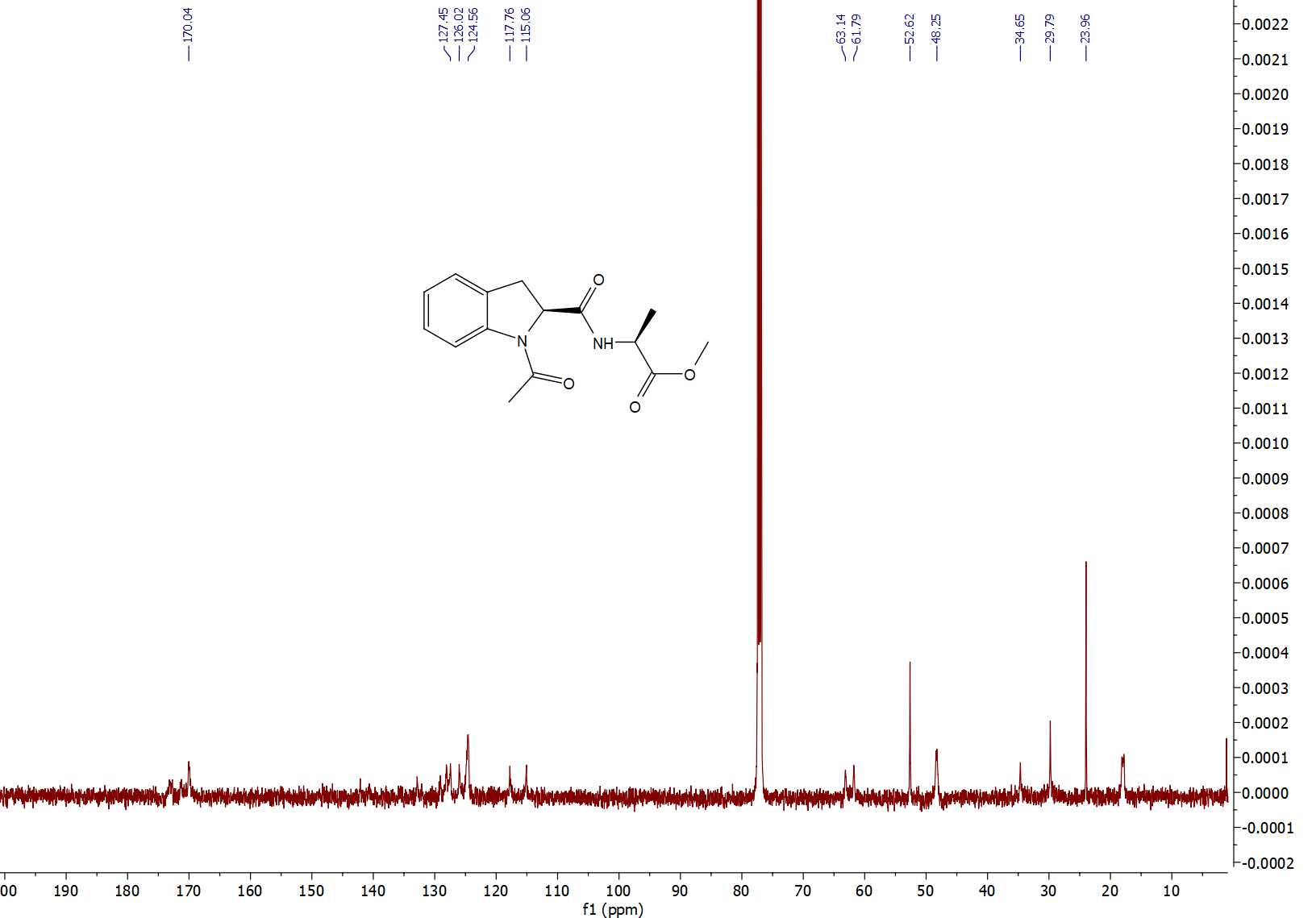


### ^1^H NMR of Ac-(2*S*)-Ind-l-Ala-OMe (**6**) in DMSO-d_6_


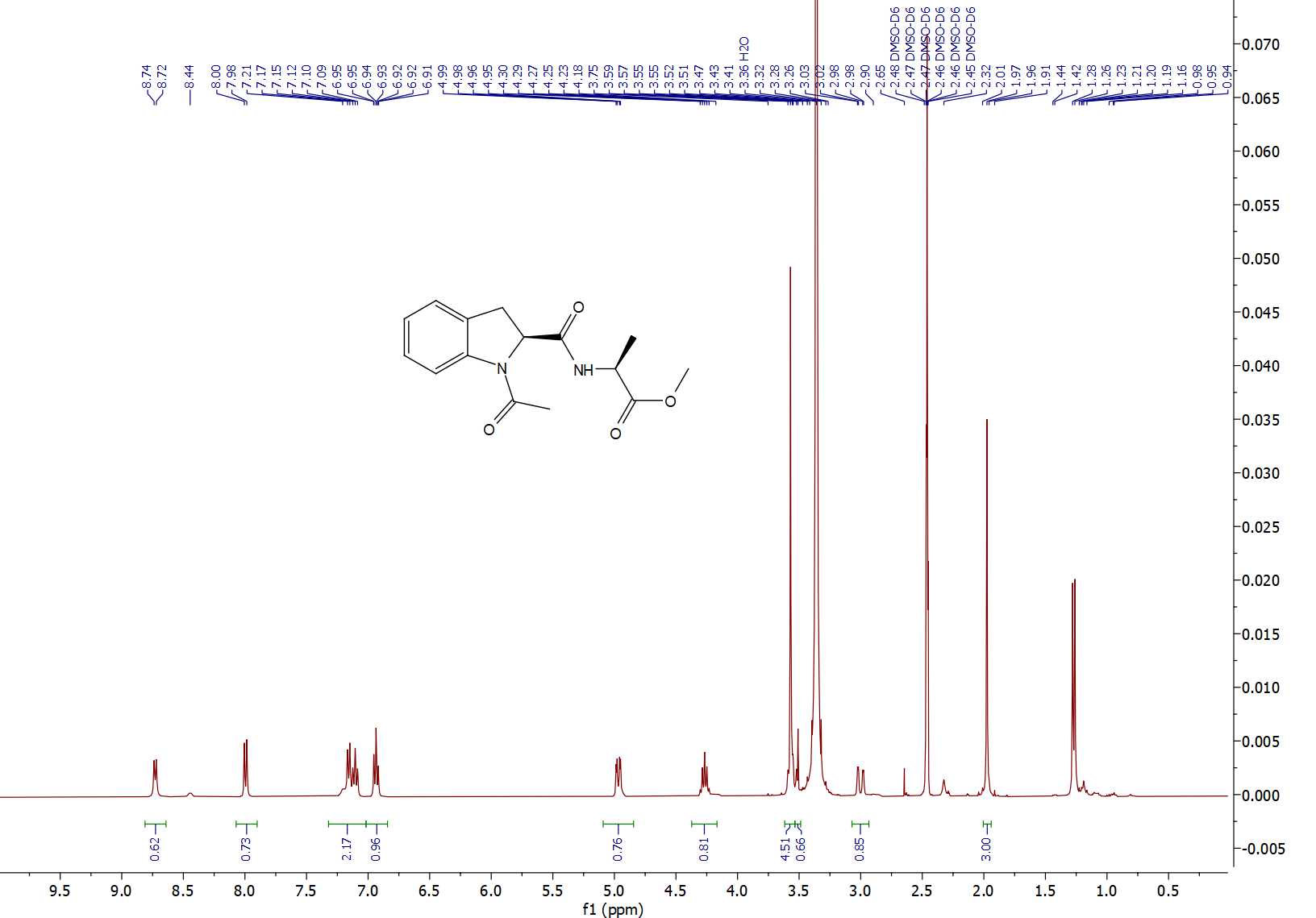


### ^1^H NMR of Boc-(2*S*)-Ind-l-Ala-OMe (**7**) in CDCl_3_

***
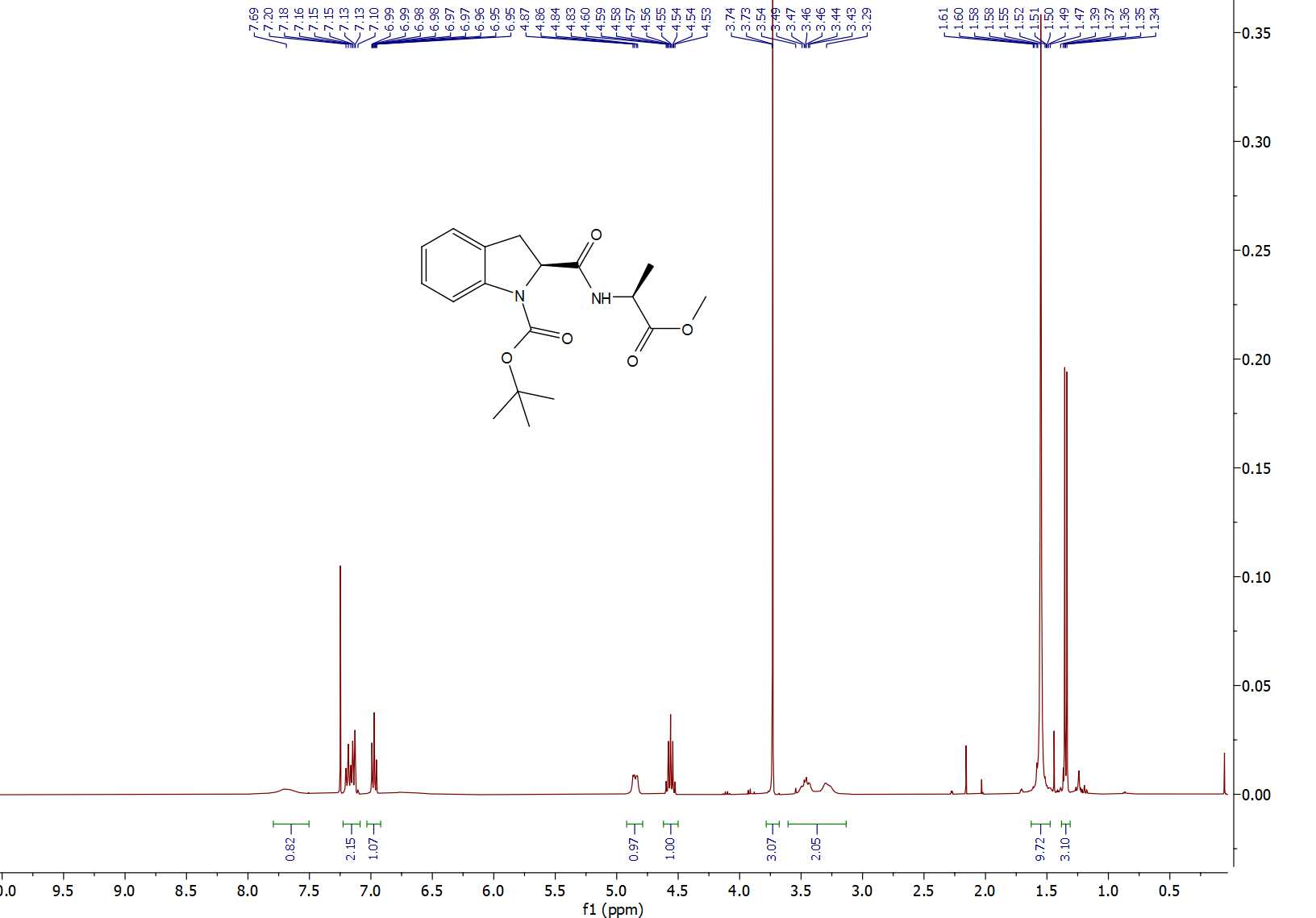
***

### ^13^C NMR of Boc-(2*S*)-Ind-l-Ala-OMe (**7**) in CDCl_3_

***
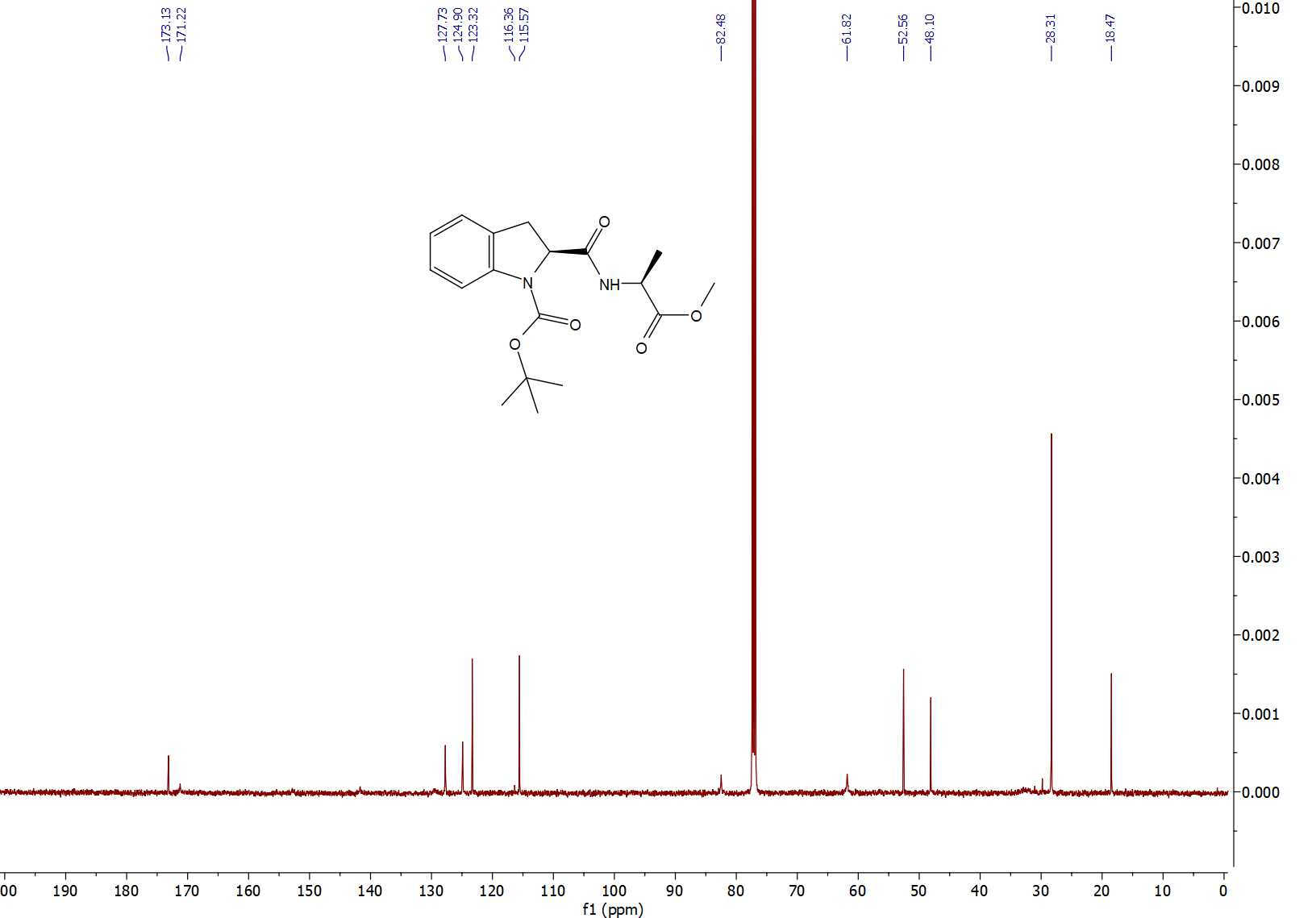
***

### ^1^H NMR of Ac-(2*S*)-Ind-d-Ala-OMe (**8**) in CDCl_3_

***
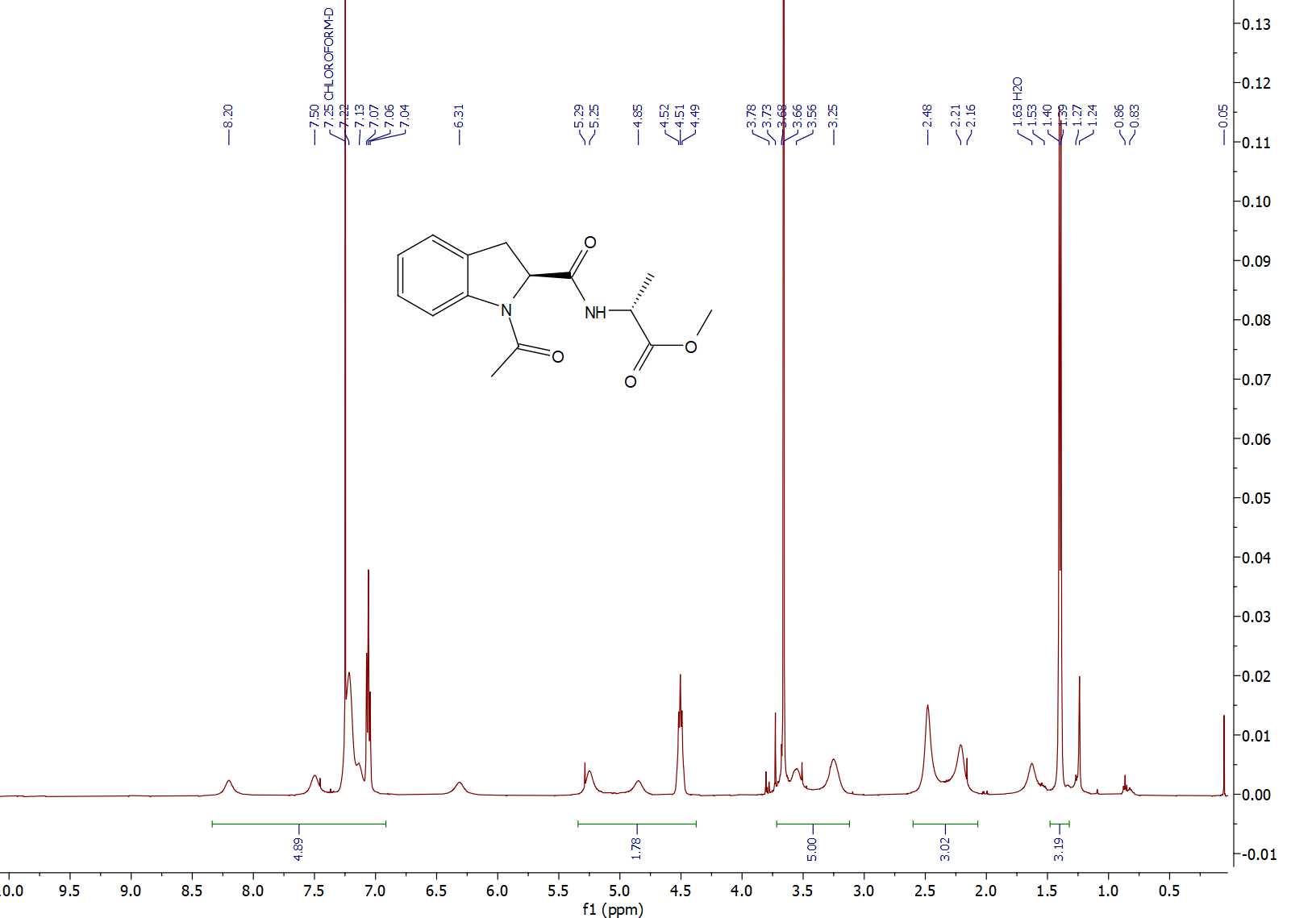
***

### ^13^C NMR of Ac-(2*S*)-Ind-d-Ala-OMe (**8**) in CDCl_3_

***
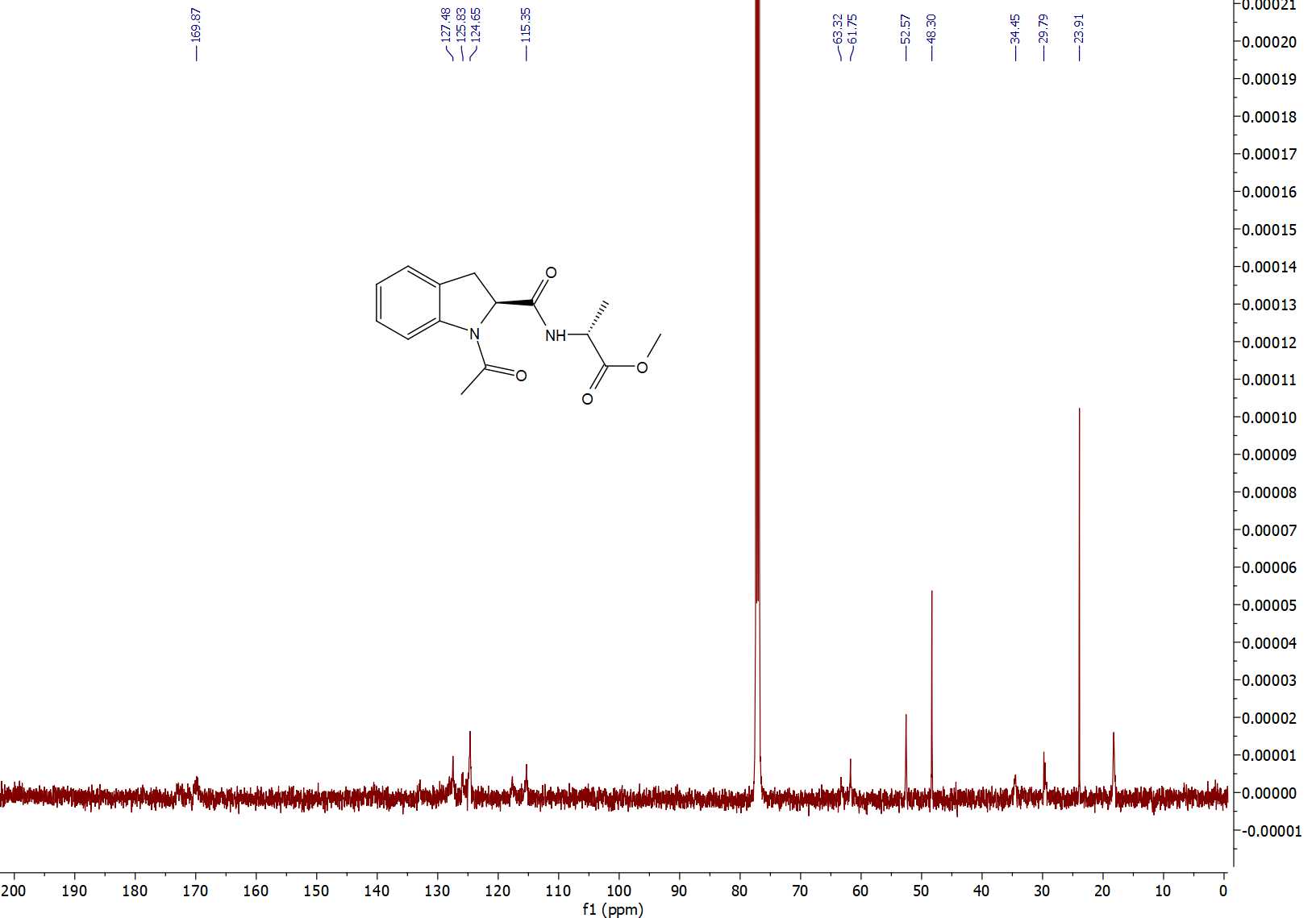
***

### ^1^H NMR of Ac-(2*S*)-Ind-d-Ala-OMe (**8**) in DMSO-d_6_

^
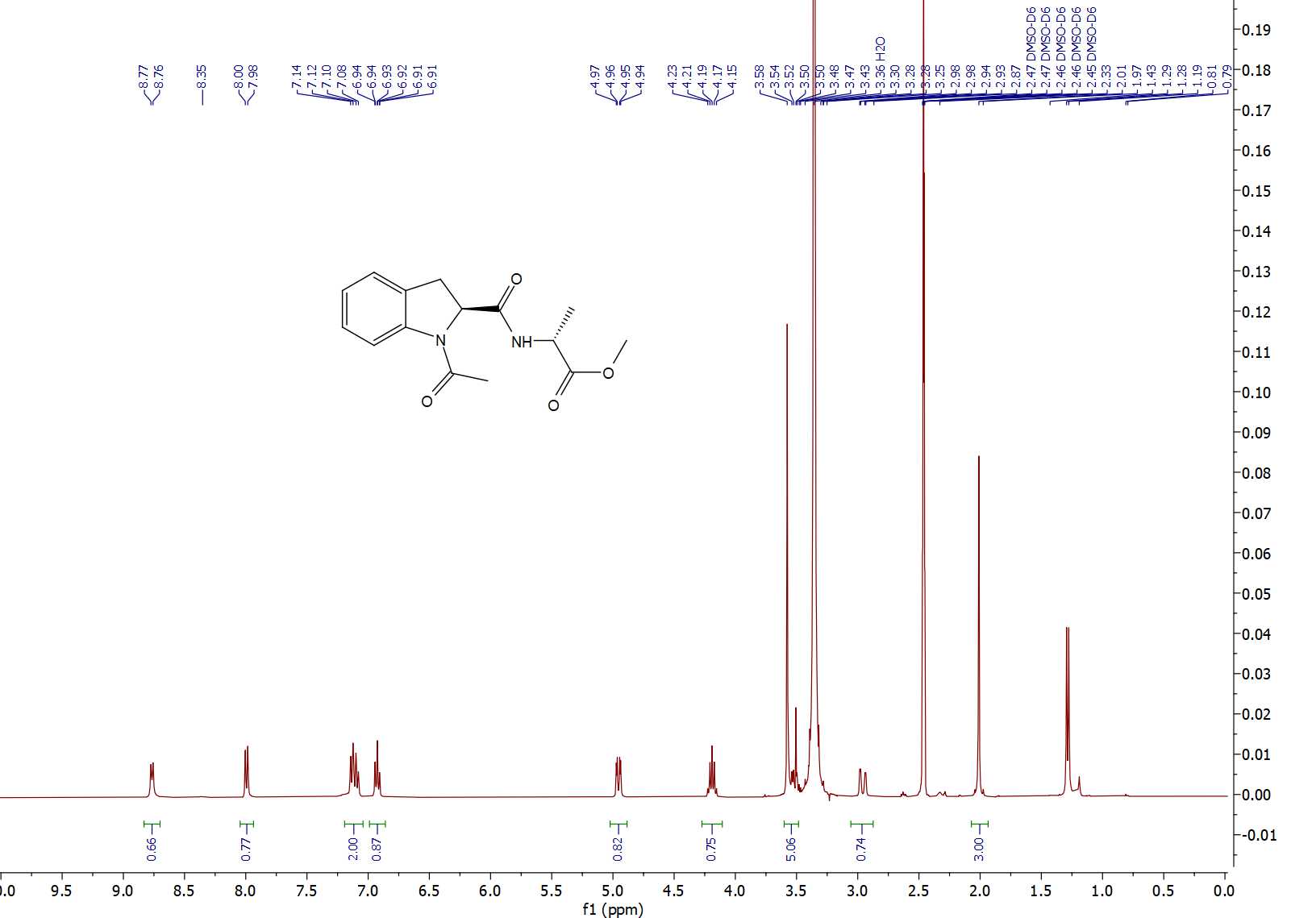
^

### ^1^H NMR of Boc-(2*S*)-Ind-d-Ala-OMe (**9**) in CDCl_3_

***
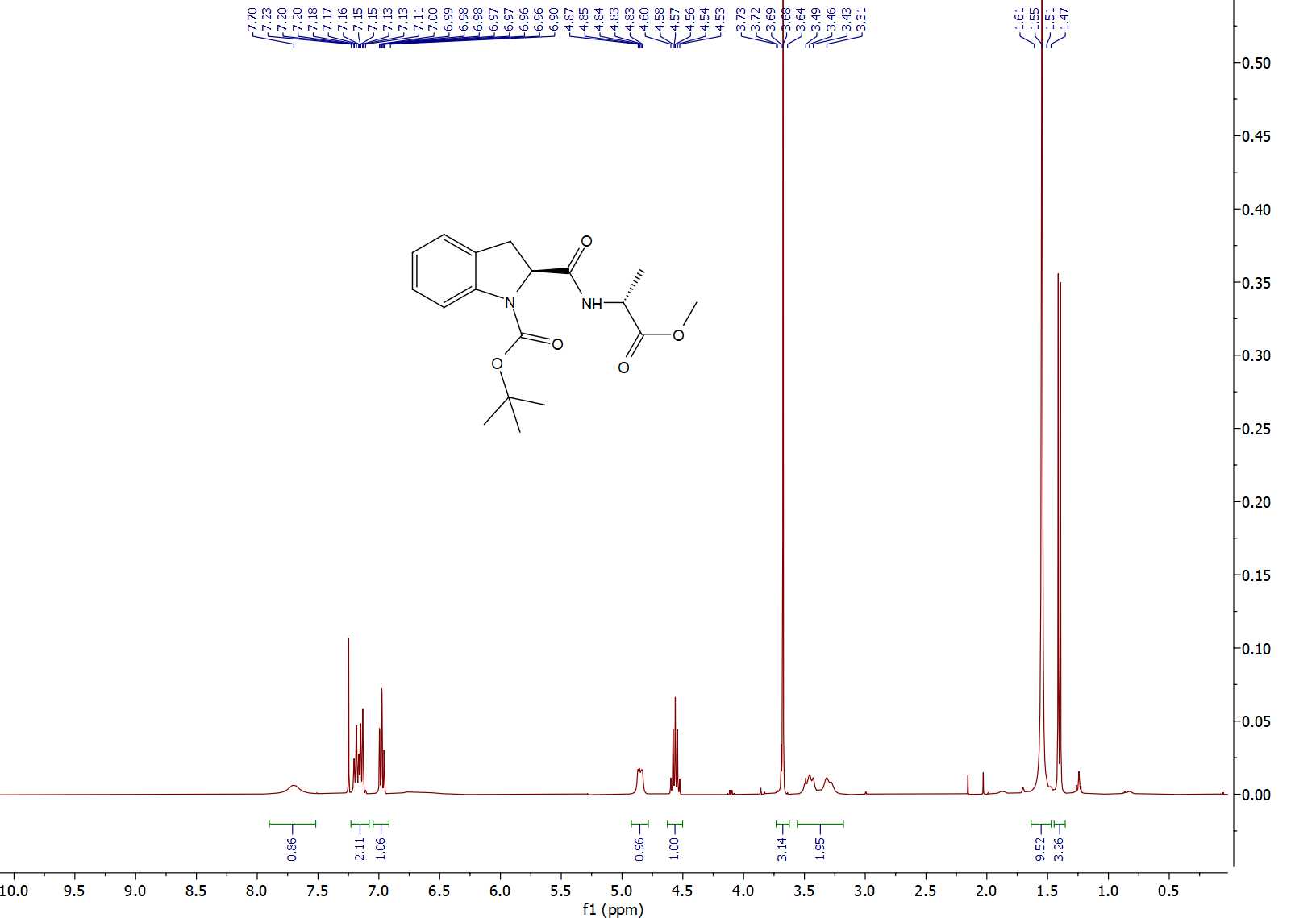
***

### ^13^C NMR of Boc-(2*S*)-Ind-d-Ala-OMe (**9**) in CDCl_3_

***
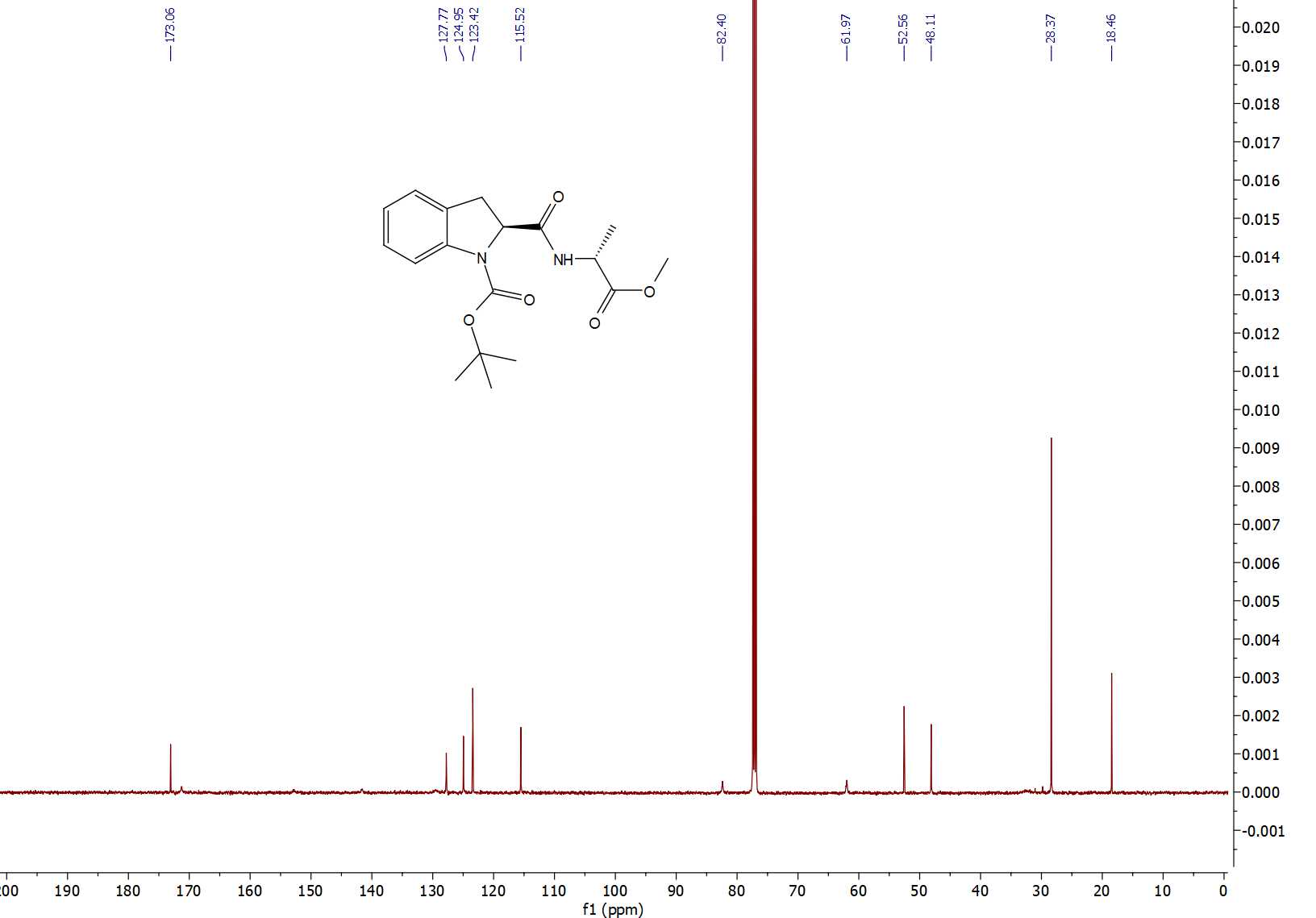
***

### ^1^H NMR of Boc-l-Ala-(2*S*)-Ind-OMe (**10**) in CDCl_3_

***
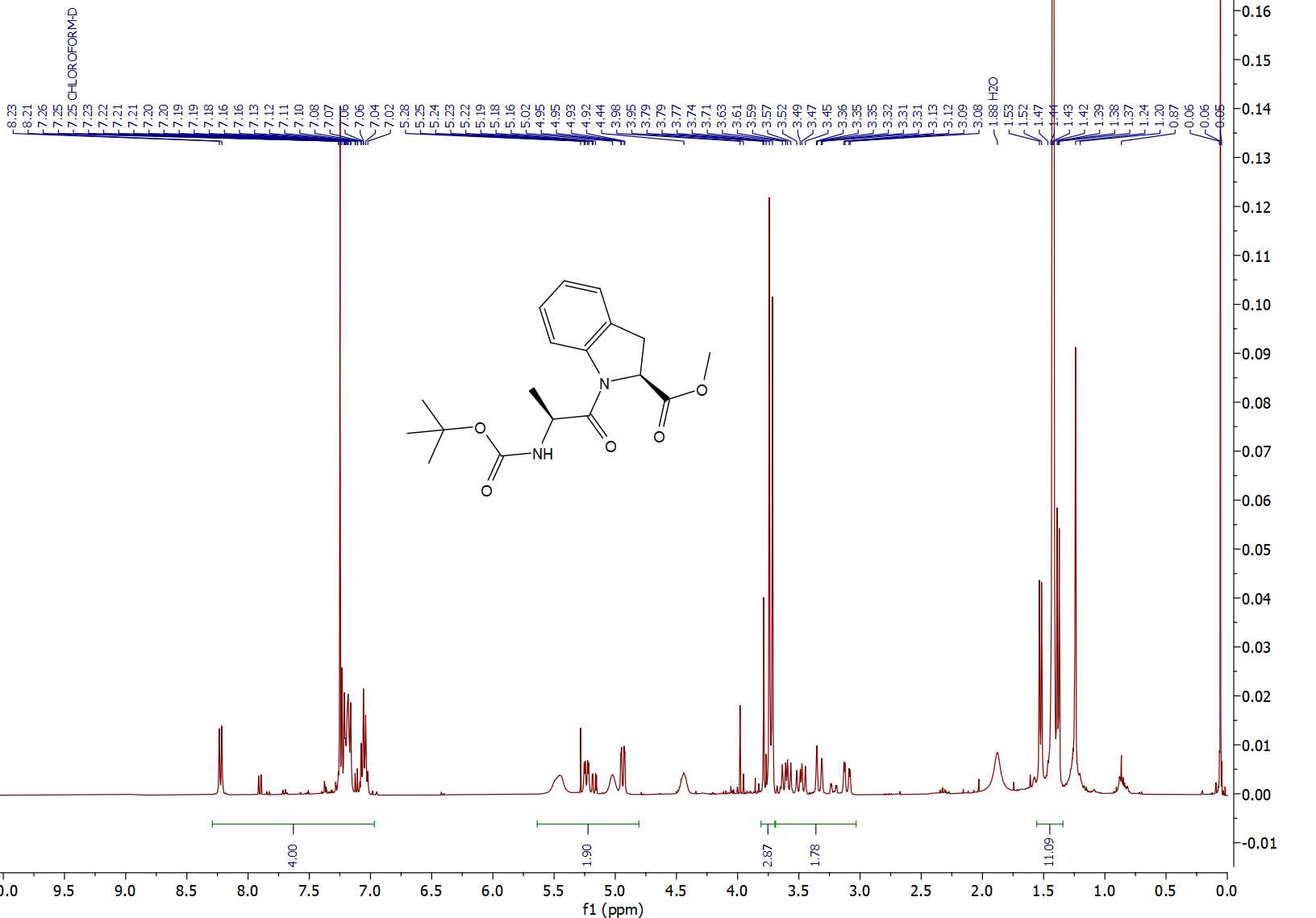
***

### ^13^C NMR of Boc-l-Ala-(2*S*)-Ind-OMe (**10**) in CDCl_3_

***
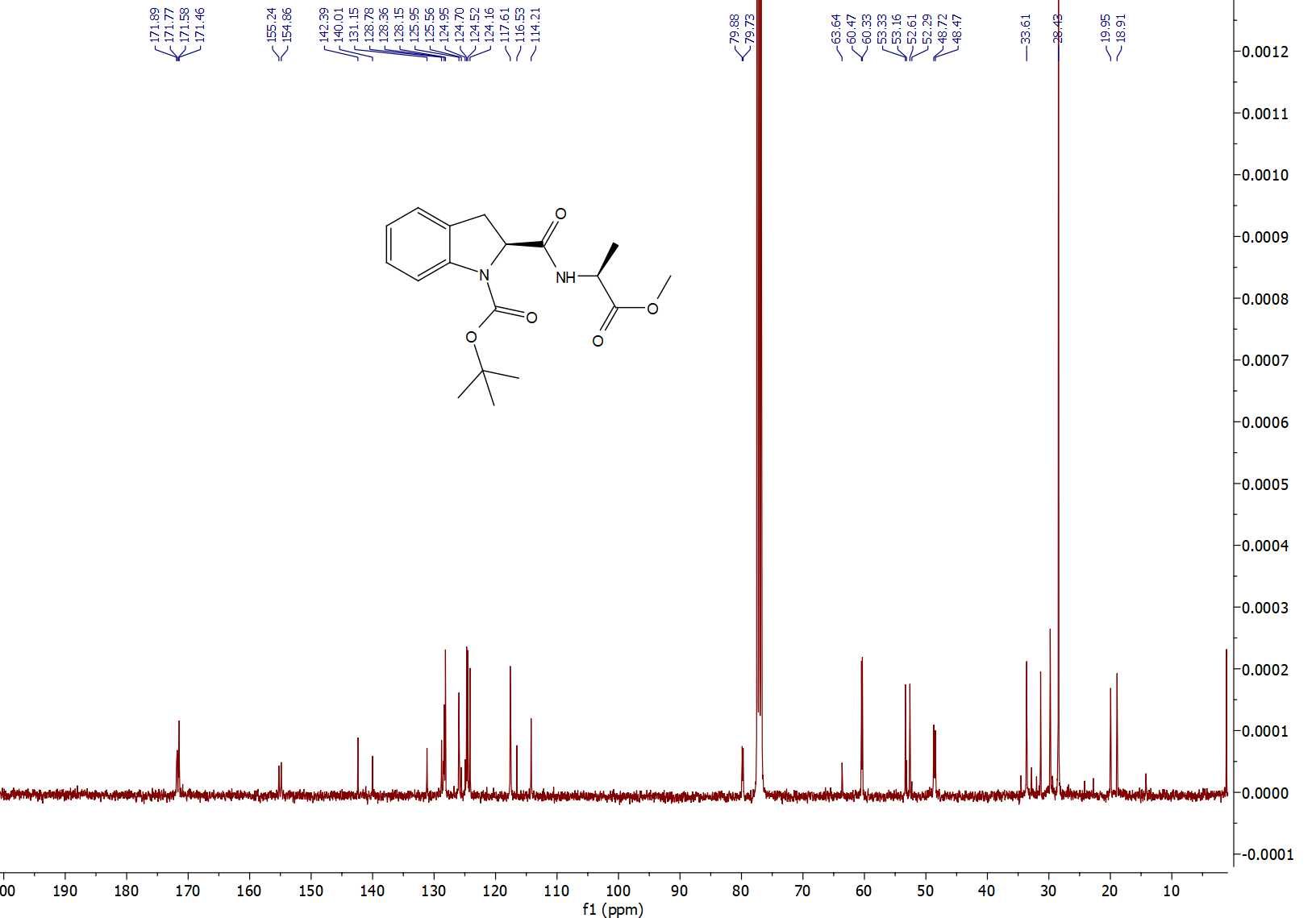
***

### ^1^H NMR of Boc-d-Ala-(2*S*)-Ind-OMe (**11**) in CDCl_3_

***
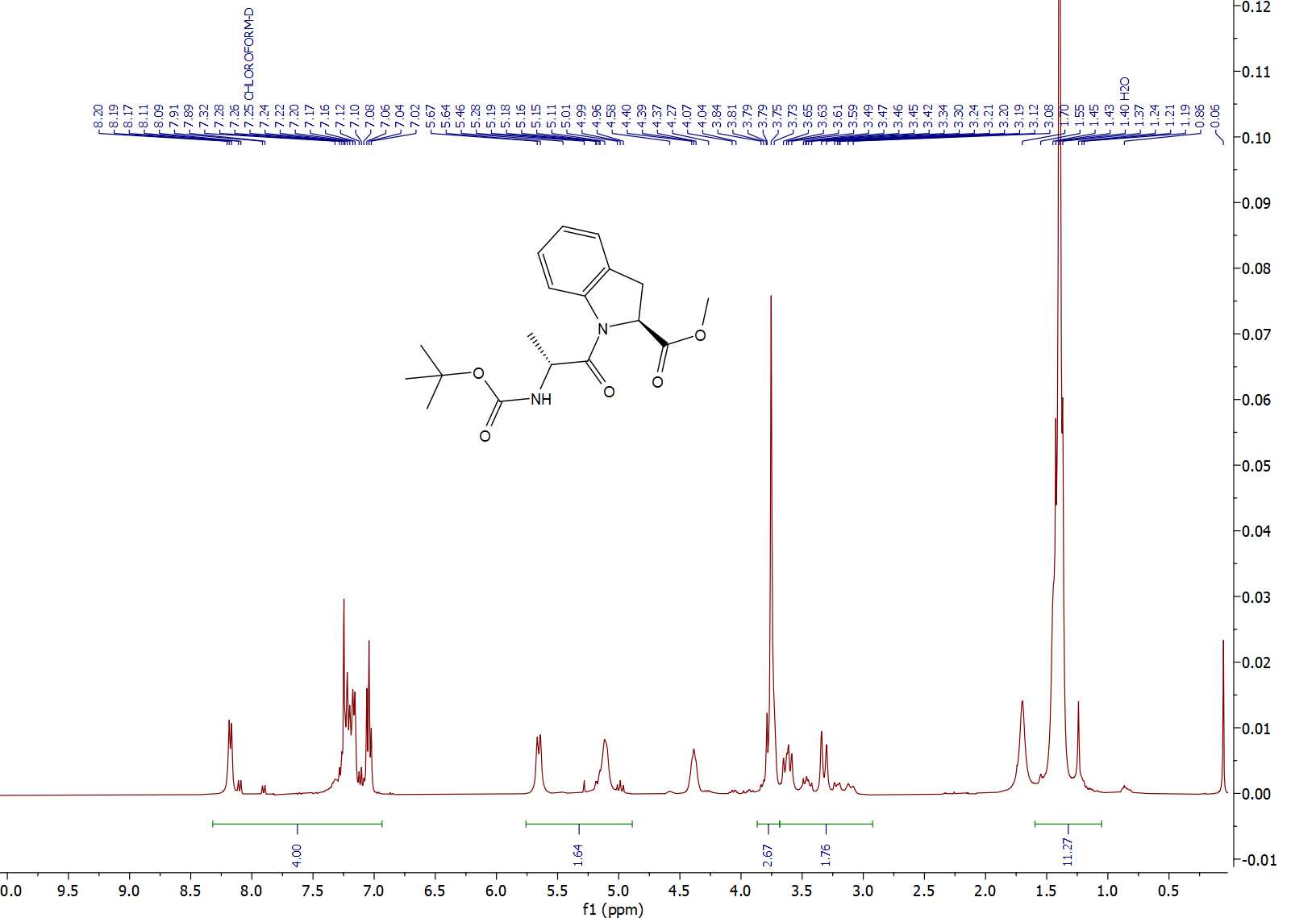
***

### ^13^C NMR of Boc-d-Ala-(2*S*)-Ind-OMe (**11**) in CDCl_3_

***
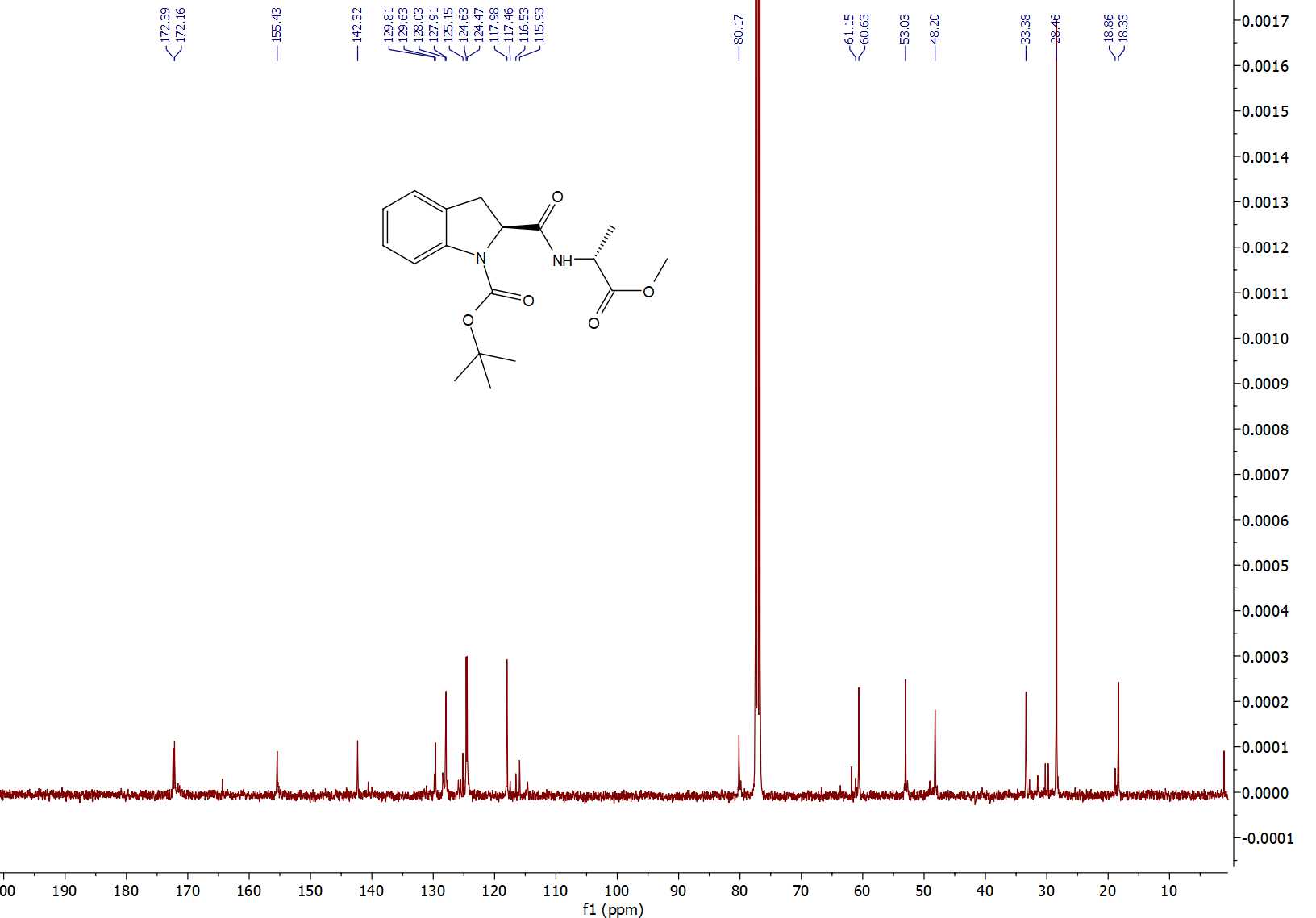
***

### ^1^H NMR Cbz-((2*S*)-Ind)_2_-OtBu (**12**) in CDCl_3_


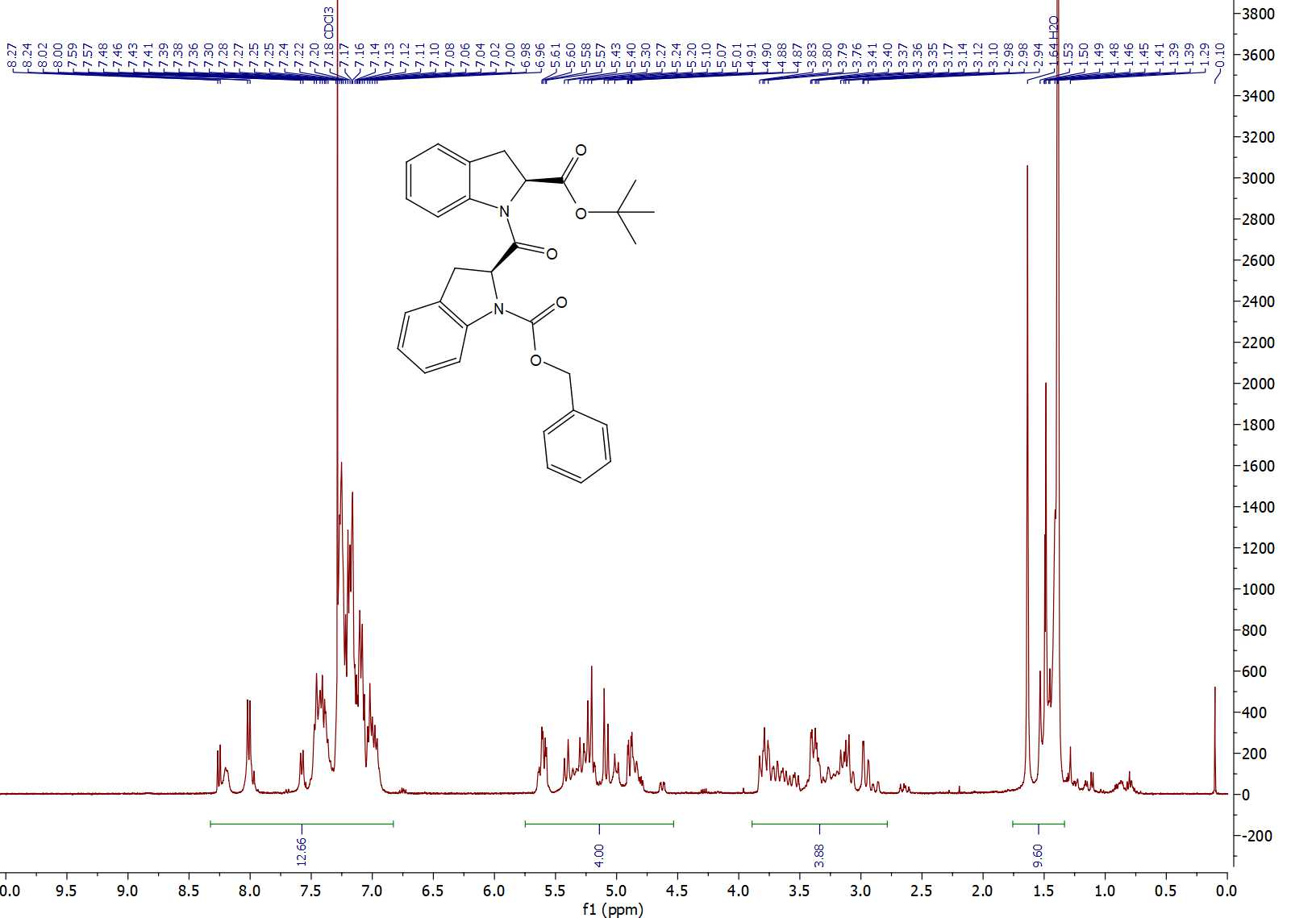


### DEPT ^13^C NMR Cbz-((2*S*)-Ind)_2_-OtBu (**12**) in CDCl_3_


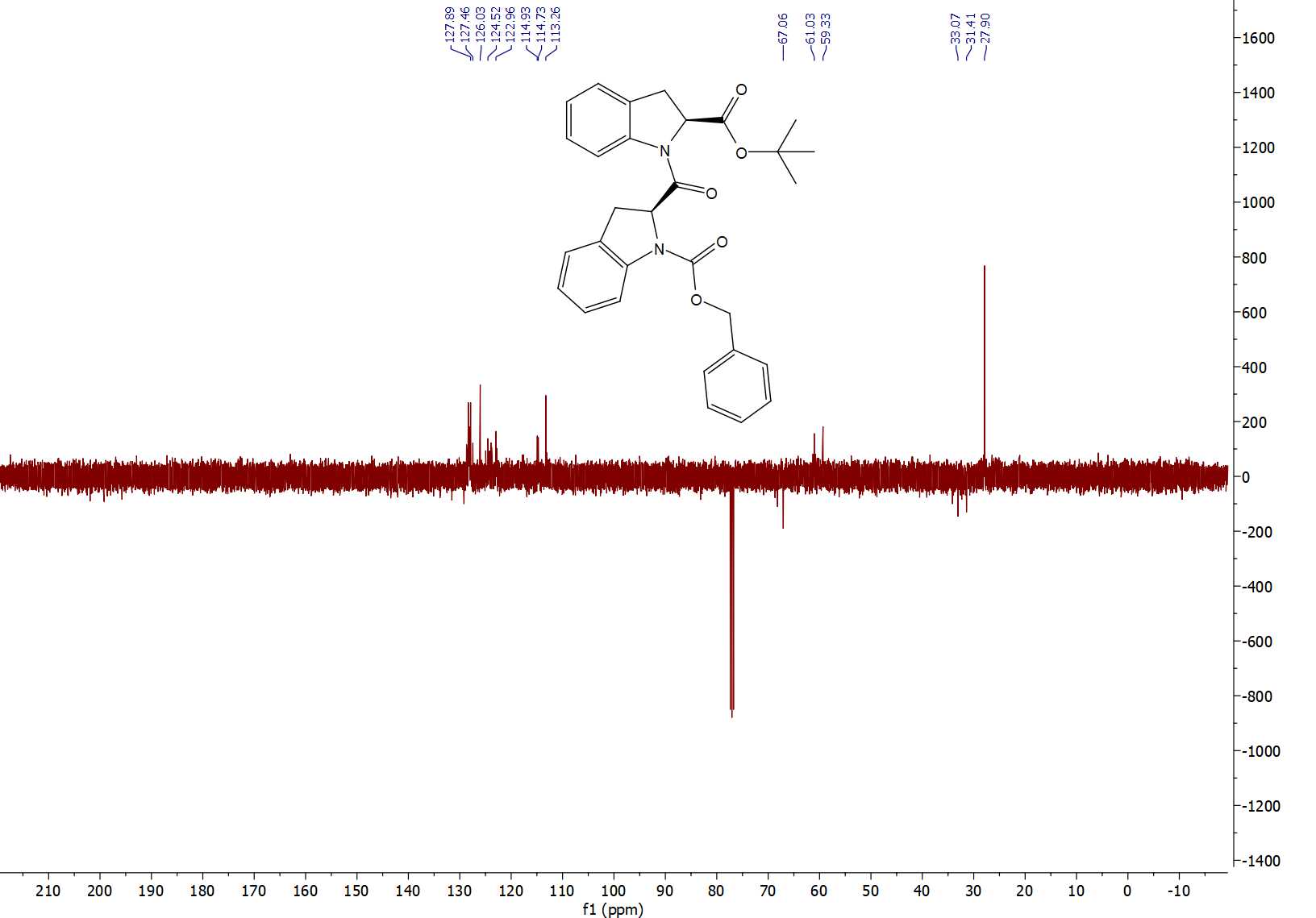


### ^1^H NMR of Boc-Gly-(2*S*)-Ind-OMe (**13**) in CDCl_3_

***
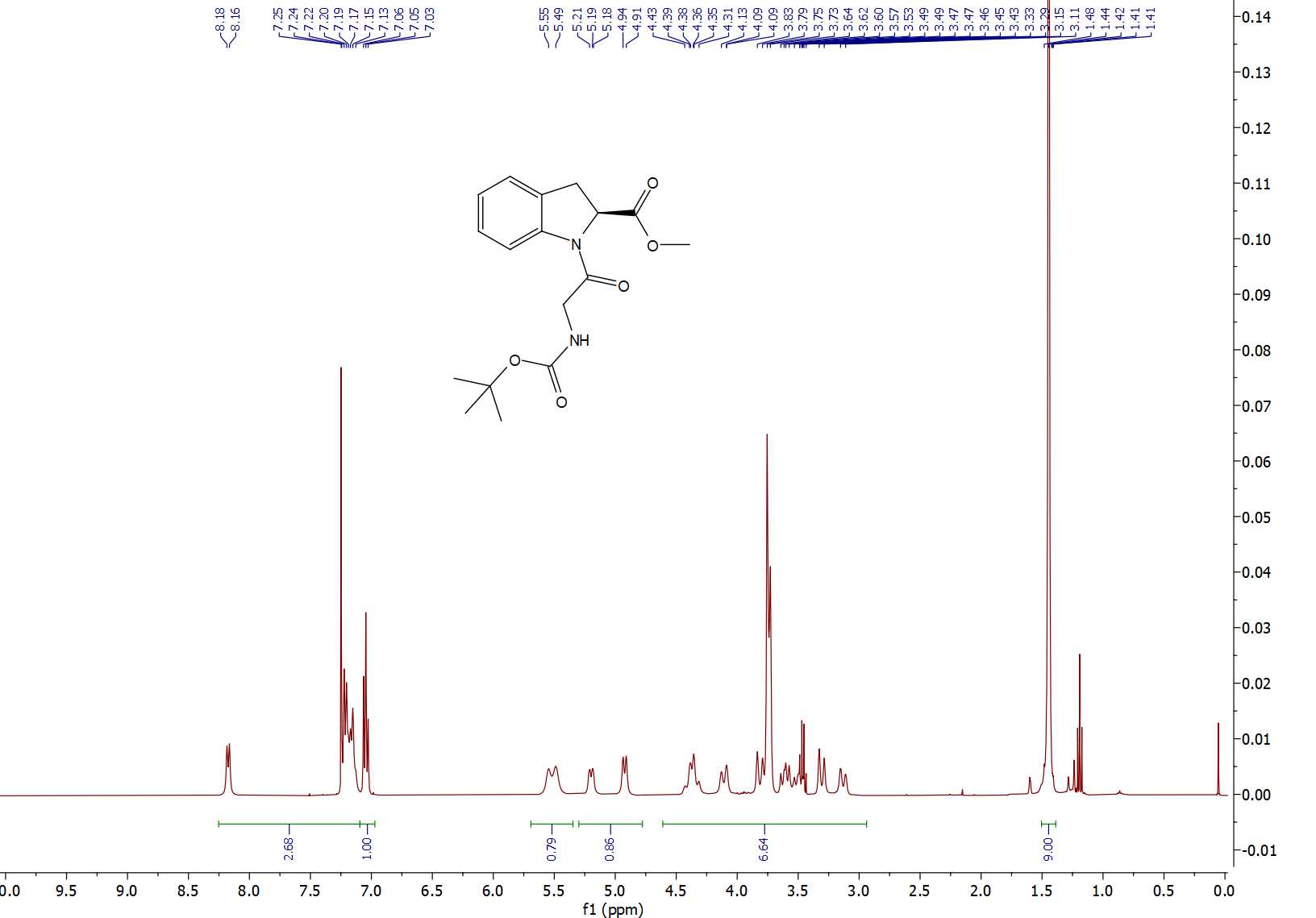
***

### ^13^C NMR of Boc-Gly-(2*S*)-Ind-OMe (**13**) in CDCl_3_

***
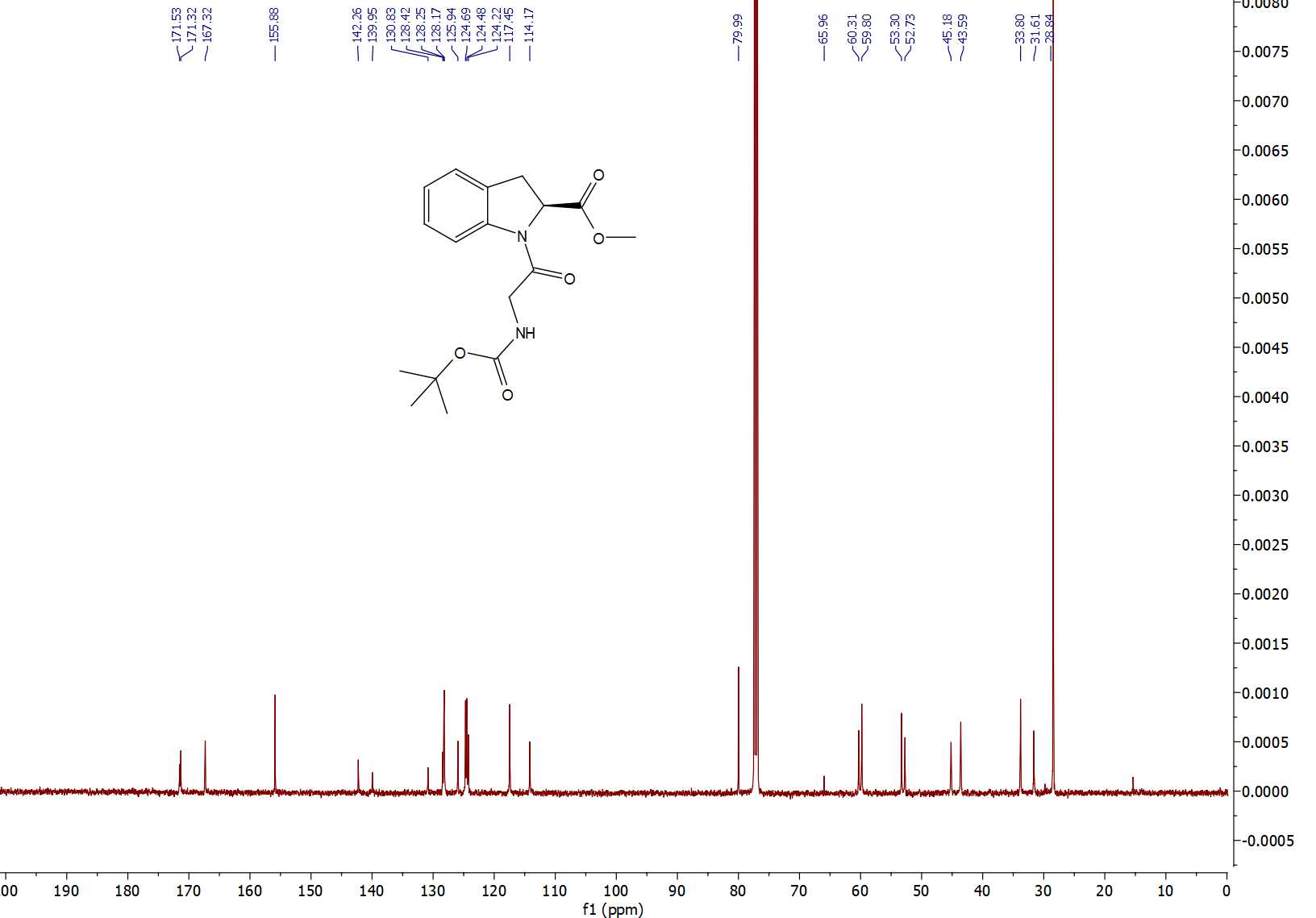
***

### ^1^H NMR of Cbz-Gly-(2*S*)-Ind-OMe (**14**) in CDCl_3_

***
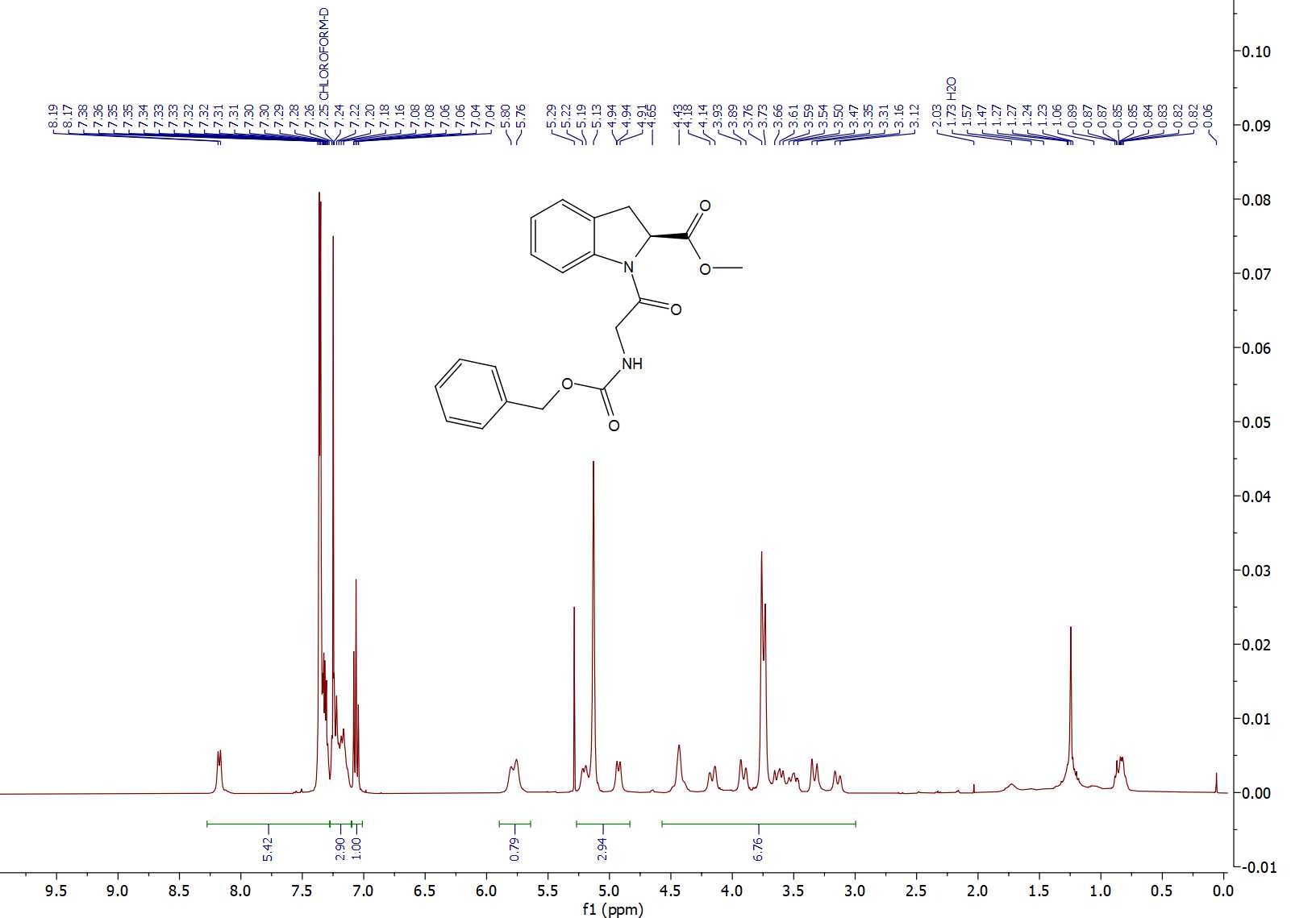
***

### ^13^C NMR of Cbz-Gly-(2*S*)-Ind-OMe (**14**) in CDCl_3_

***
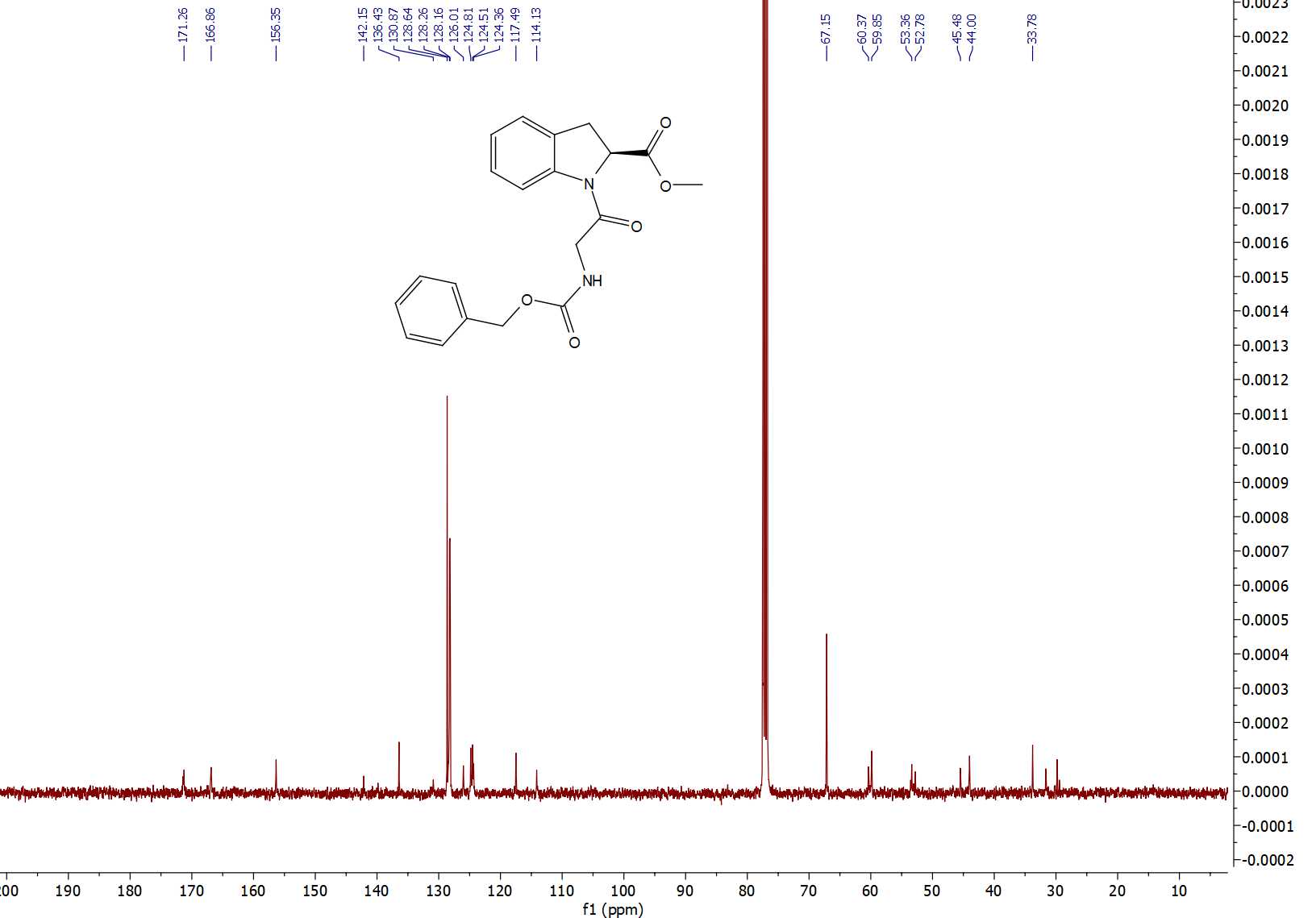
***

### ^1^ HNMR of Boc-l-Pro-(2*S*)-Ind-OMe (**15**) in CDCl_3_

***
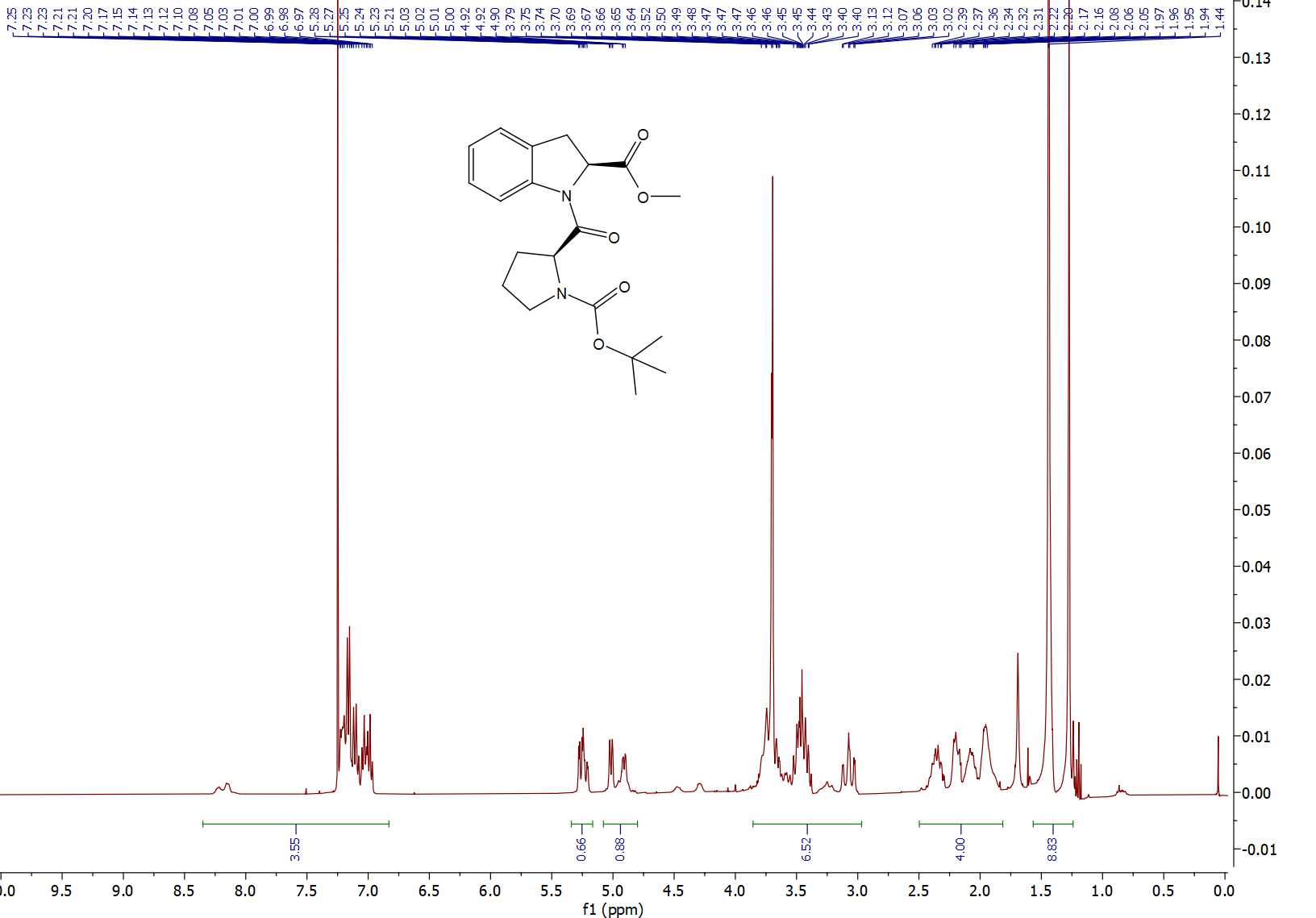
***

### ^13^C NMR of Boc-l-Pro-(2*S*)-Ind-OMe (**15**) in CDCl_3_

***
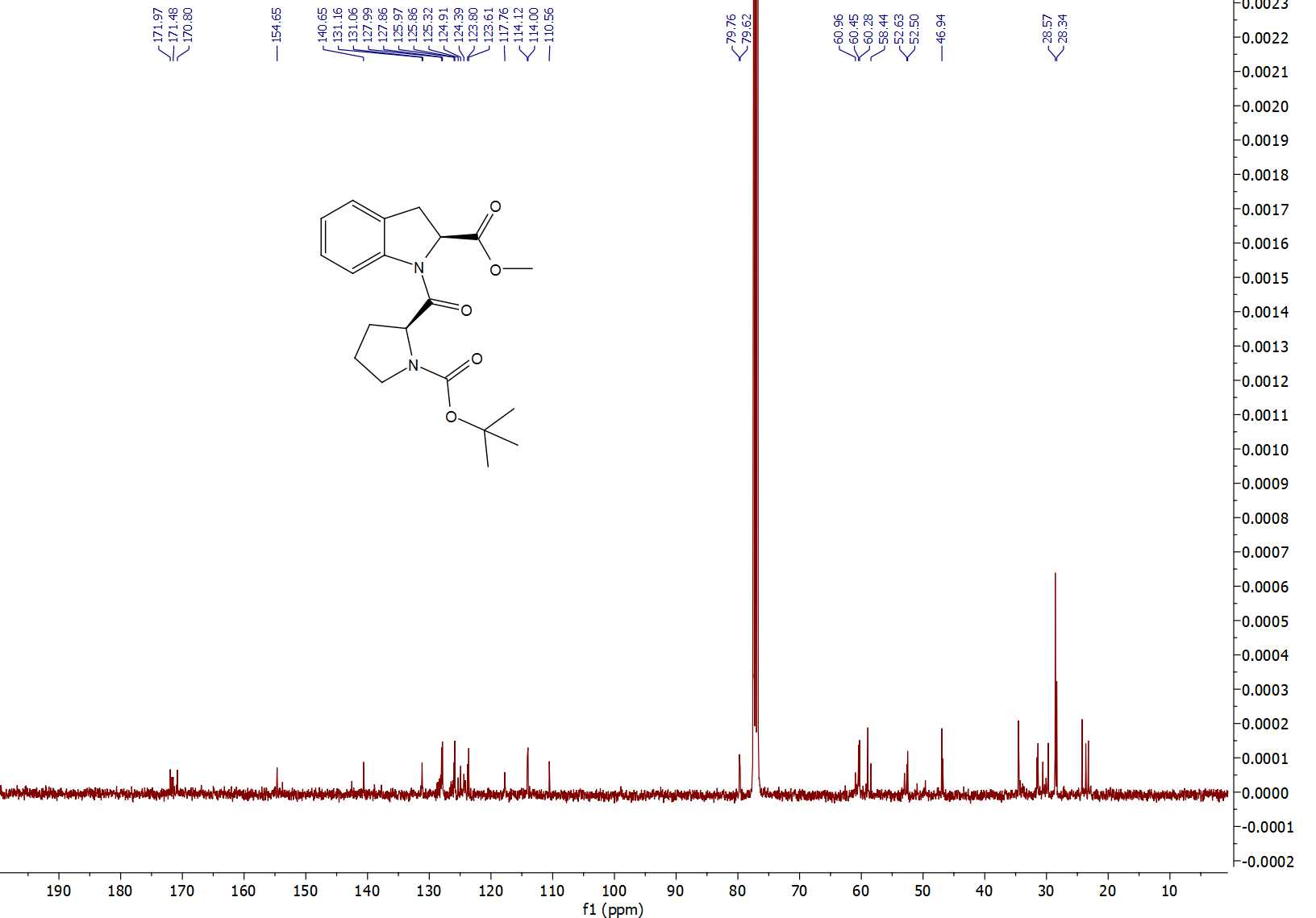
***

### ^1^H NMR of Boc-d-Pro-(2*S*)-Ind-OMe (**16**) in CDCl_3_

***
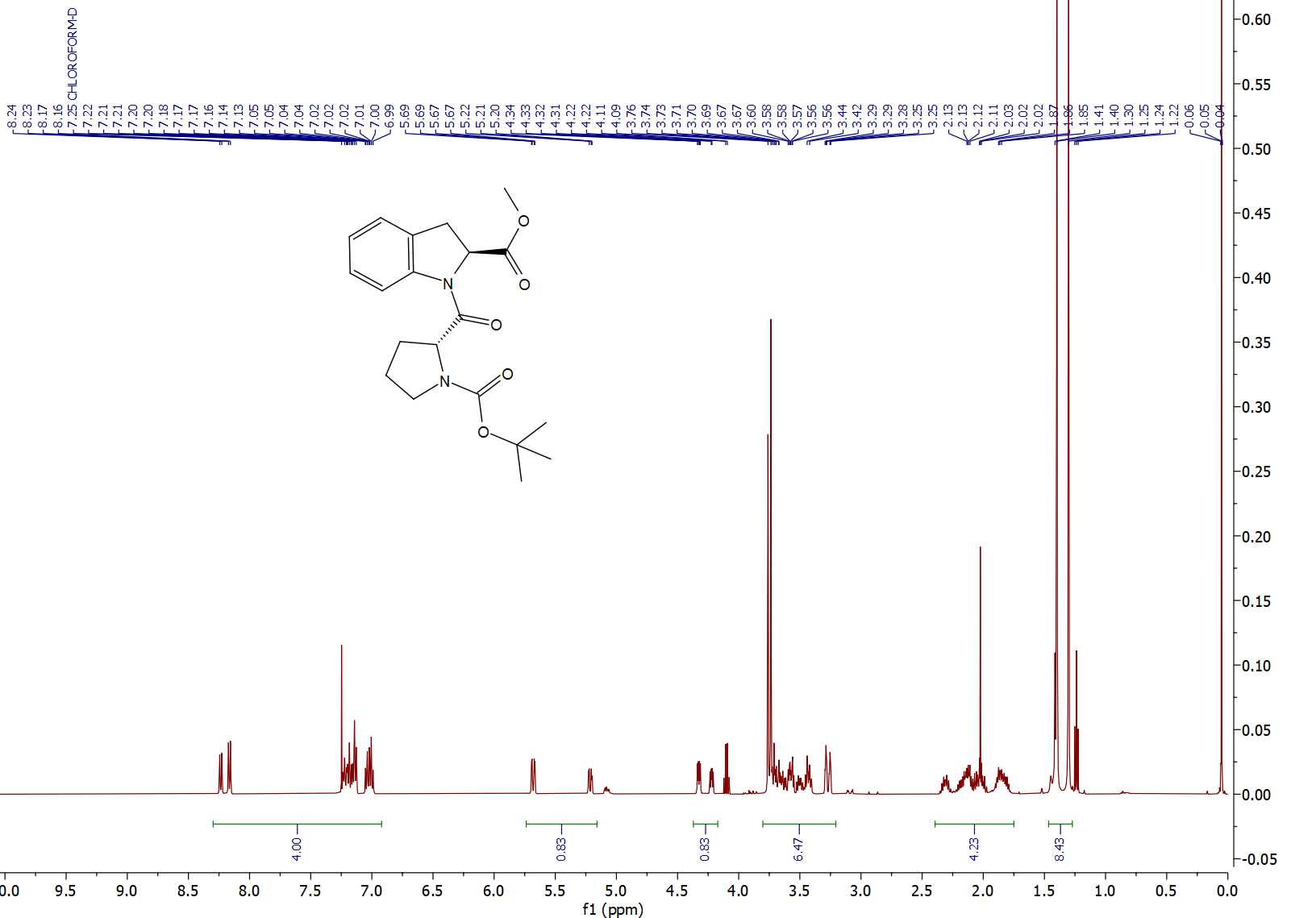
***

### ^13^C NMR of Boc-d-Pro-(2*S*)-Ind-OMe (**16**) in CDCl_3_

***
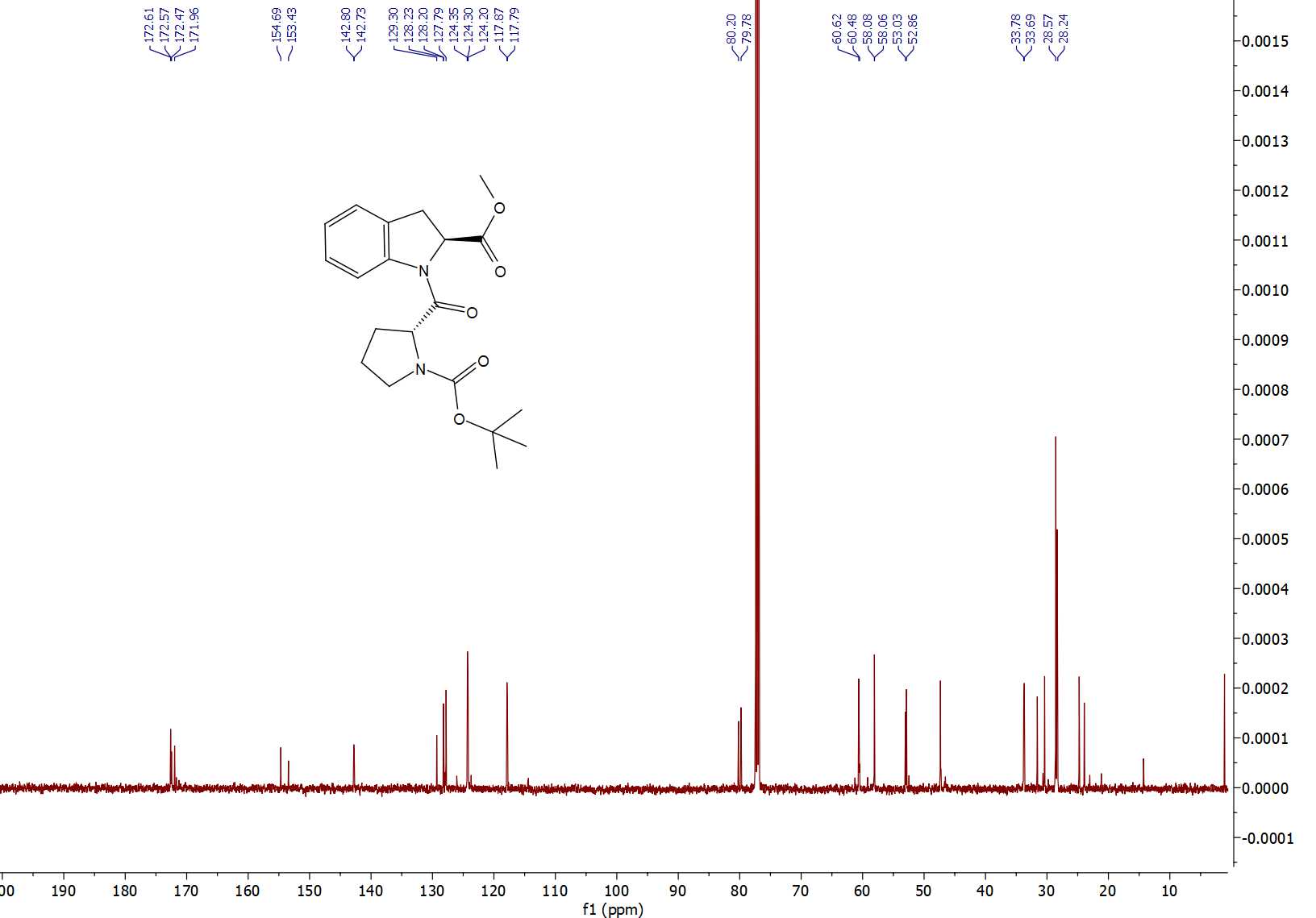
***

### ^1^H NMR of Cbz-l-Pro-(2*S*)-Ind-OMe (**17**) in CDCl_3_

***
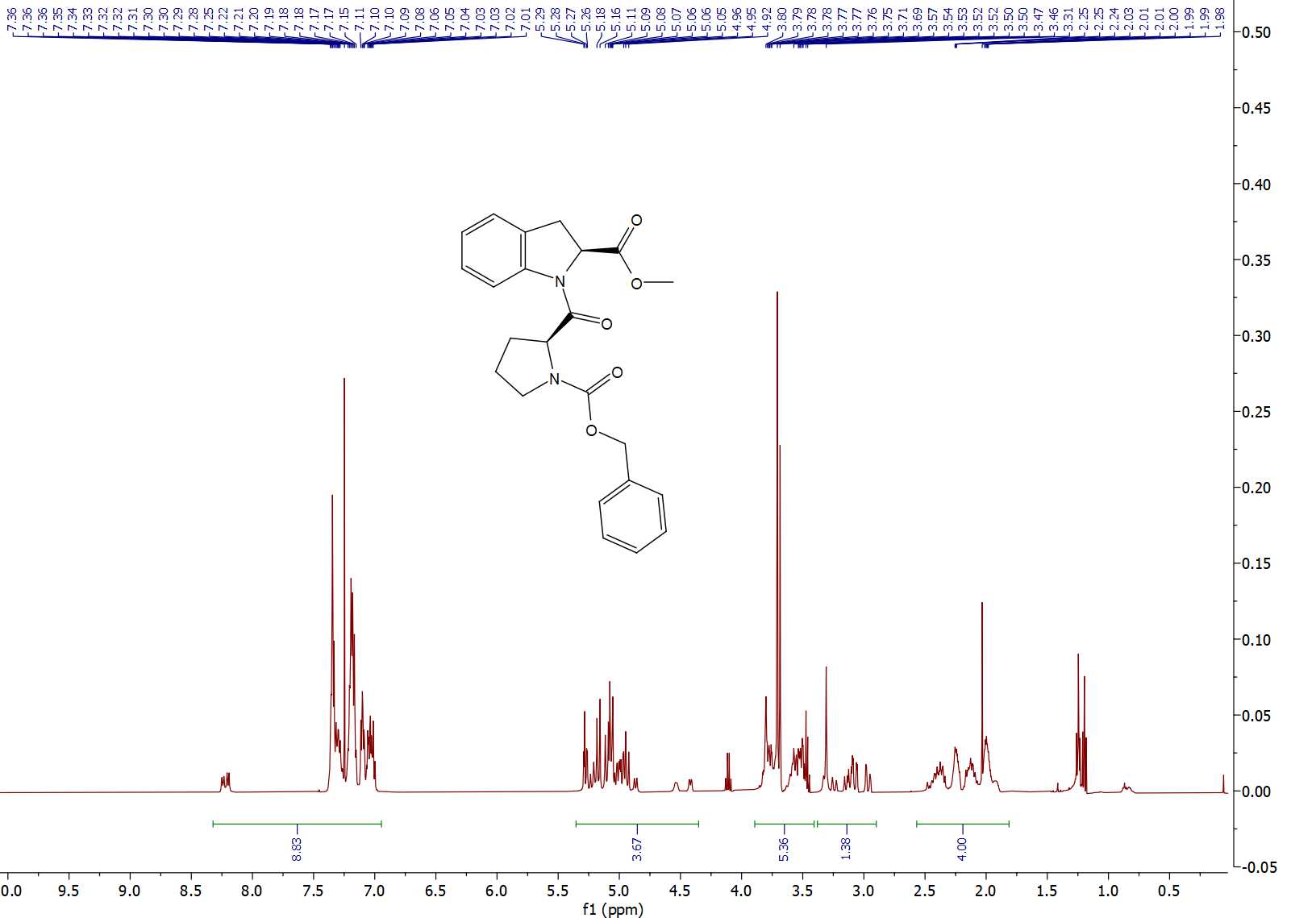
***

### ^13^C NMR of Cbz-l-Pro-(2*S*)-Ind-OMe (**17**) in CDCl_3_


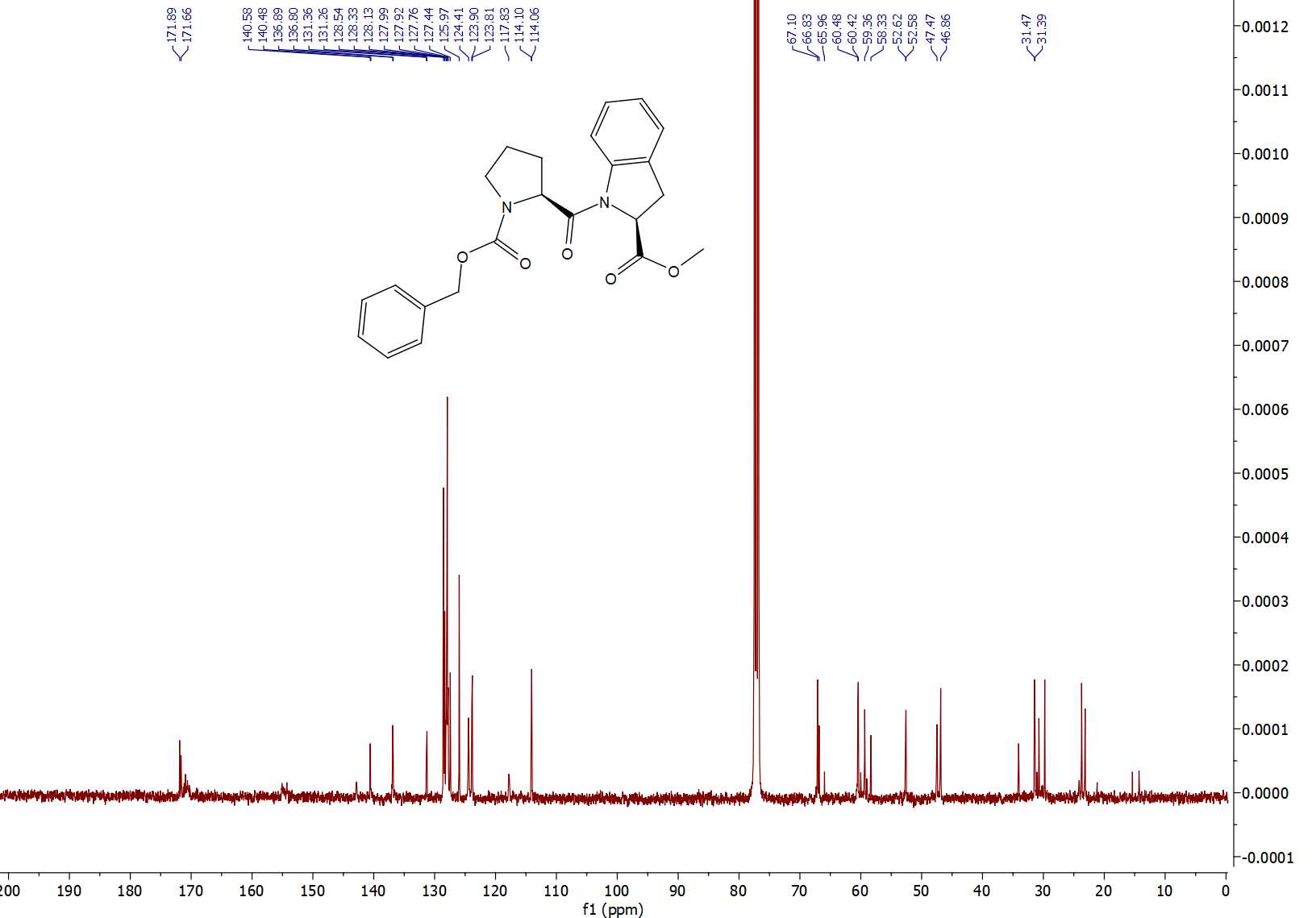


### ^1^H NMR of Cbz-l-Pro-(2*S*)-Ind-OtBu (**18**) in CDCl_3_


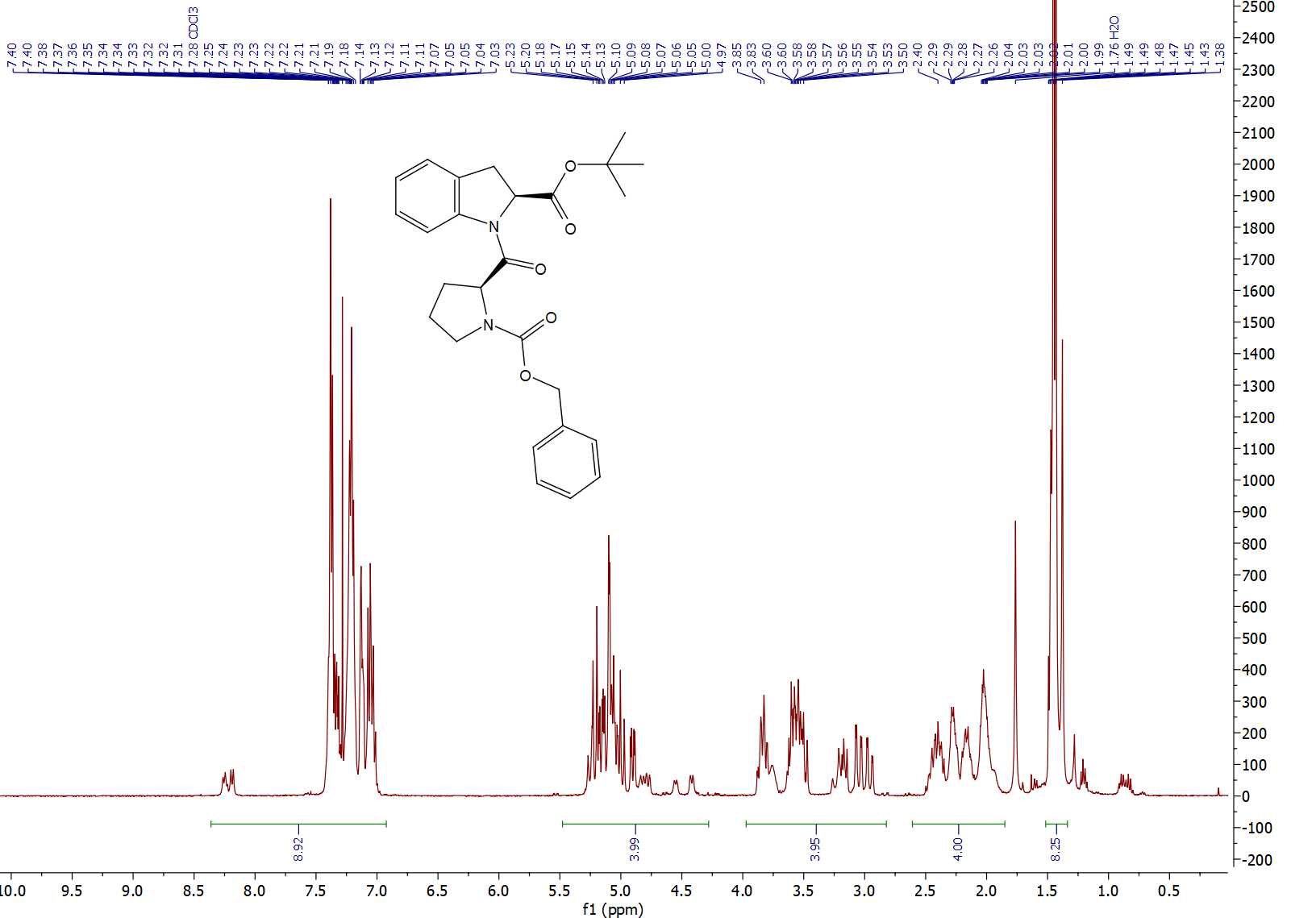


### ^13^C NMR of Cbz-l-Pro-(2*S*)-Ind-OtBu (**18**) in CDCl_3_


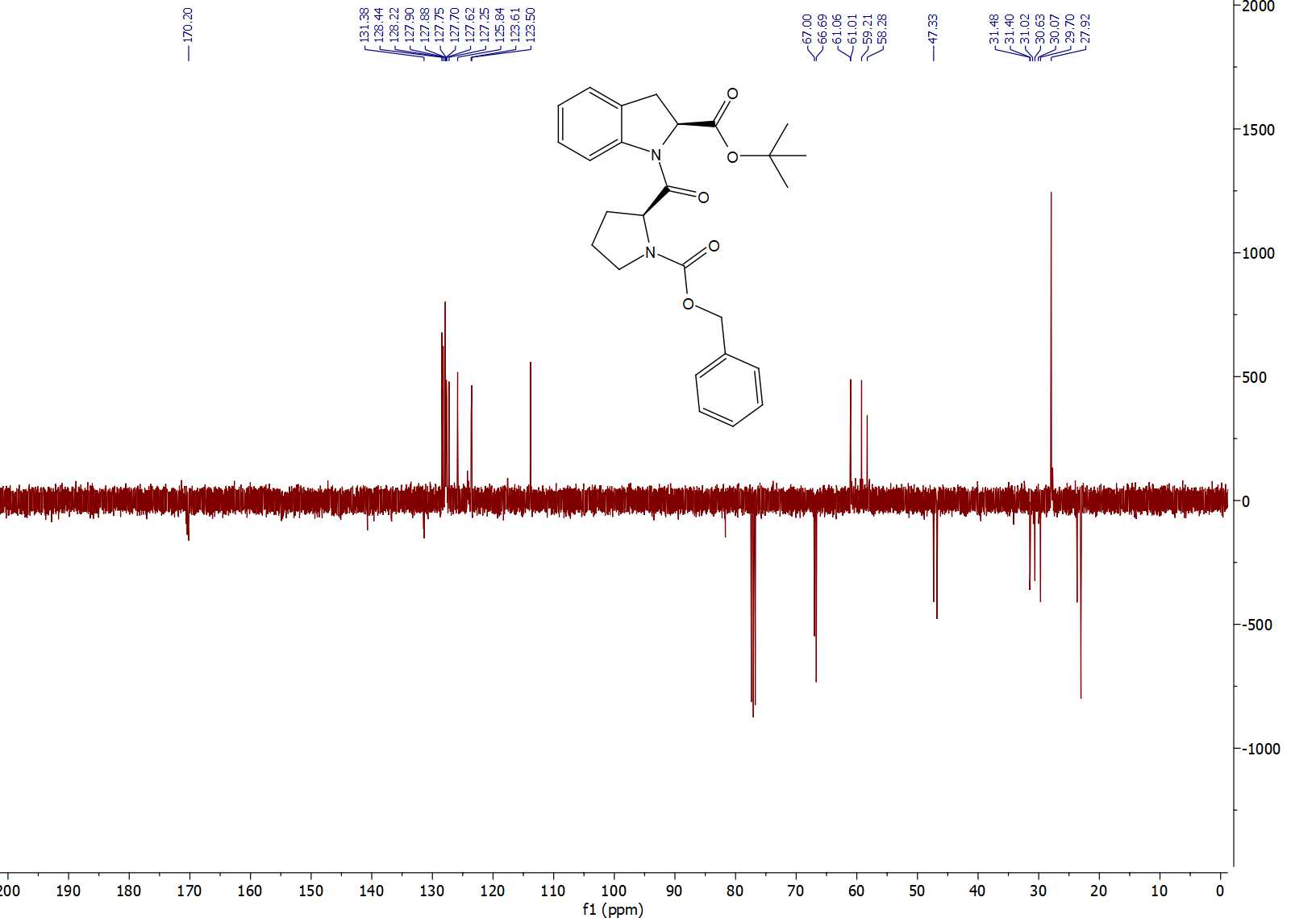


### ^1^H NMR of 2,5-diketopiperazine: Product **19** in CDCl_3_

***
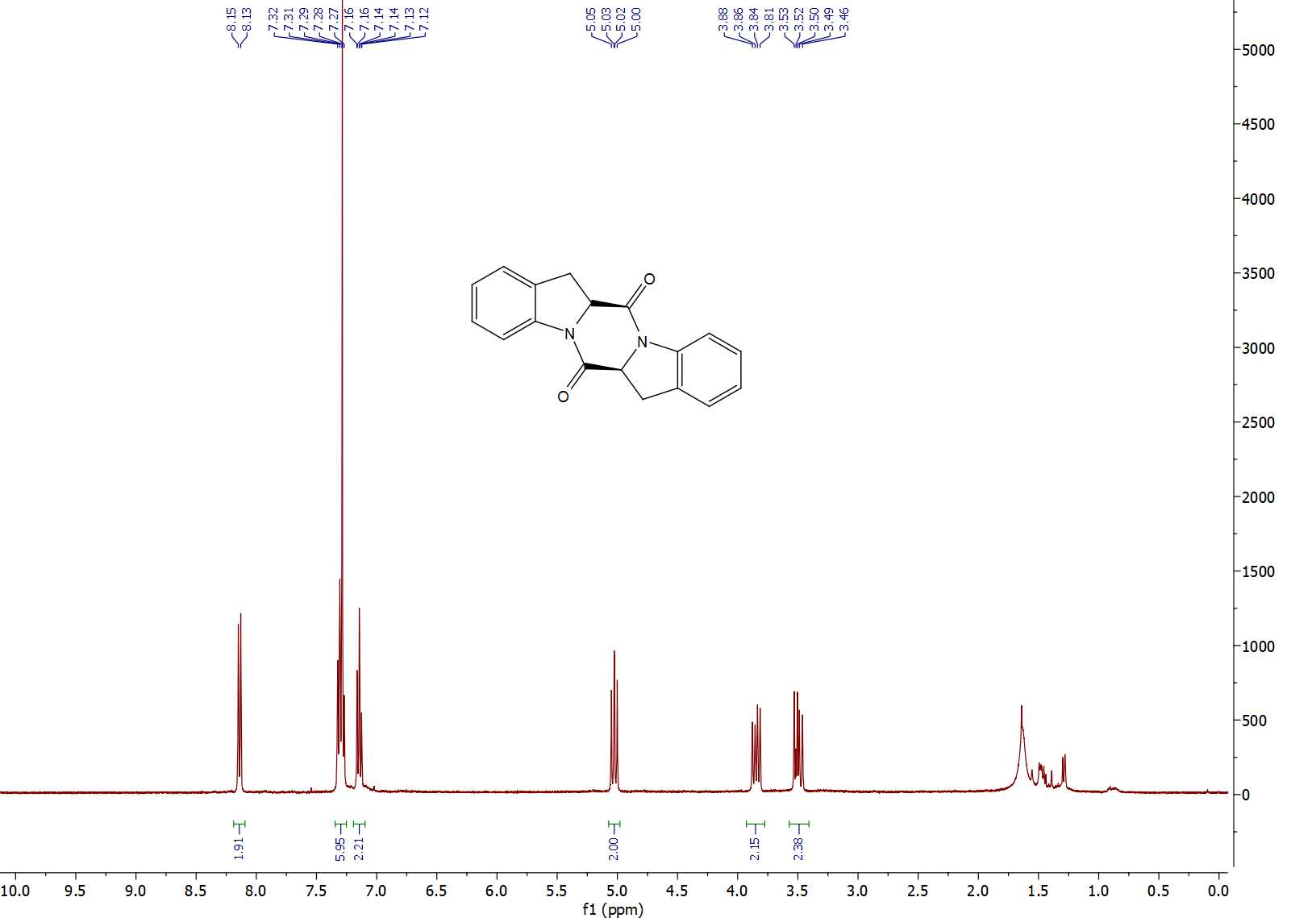
***

### ^1^H NMR of 2,5-diketopiperazine: Product **20** in CDCl_3_

***
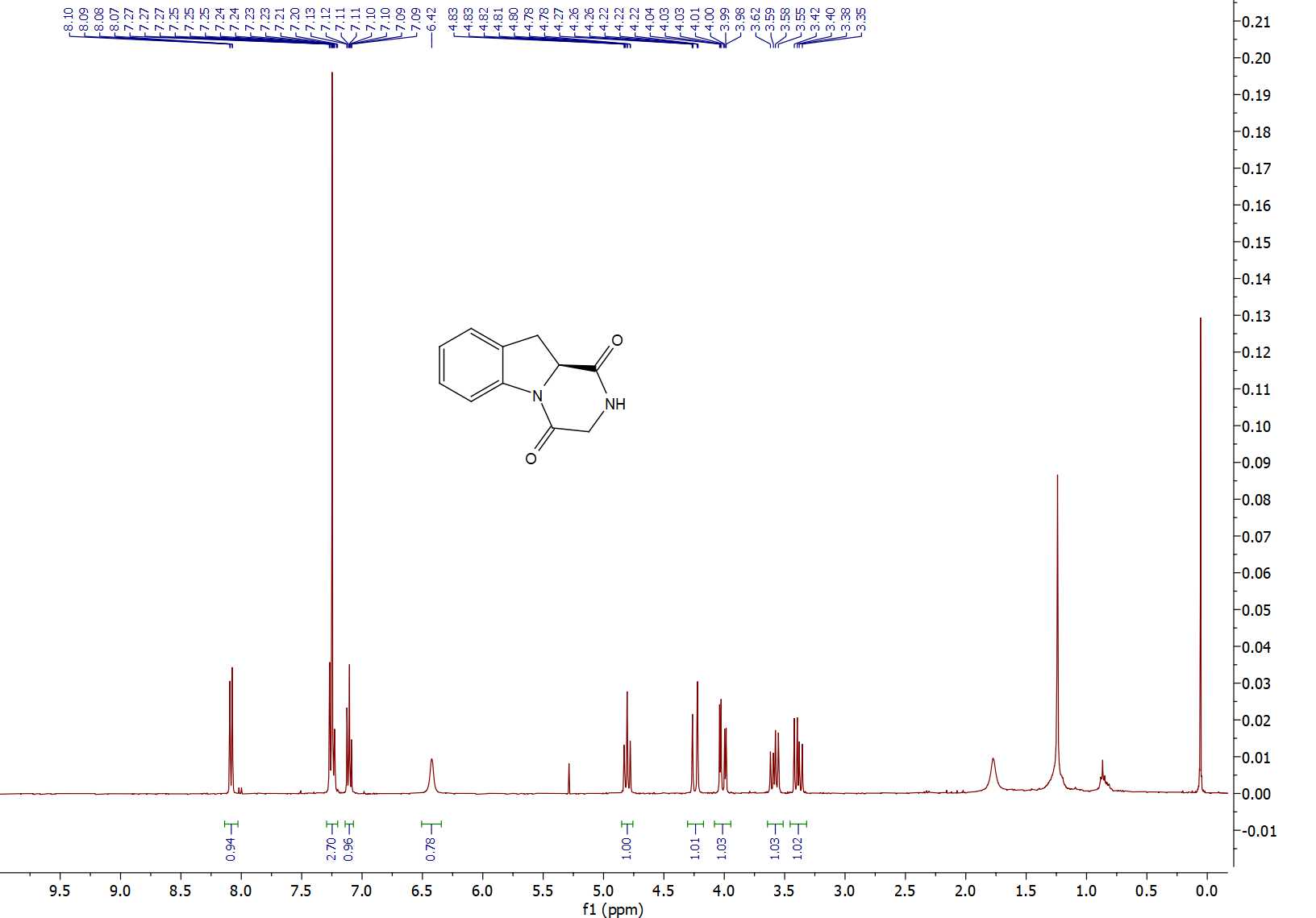
***

### ^13^C NMR of 2,5-diketopiperazine: Product **20** in CDCl_3_

***
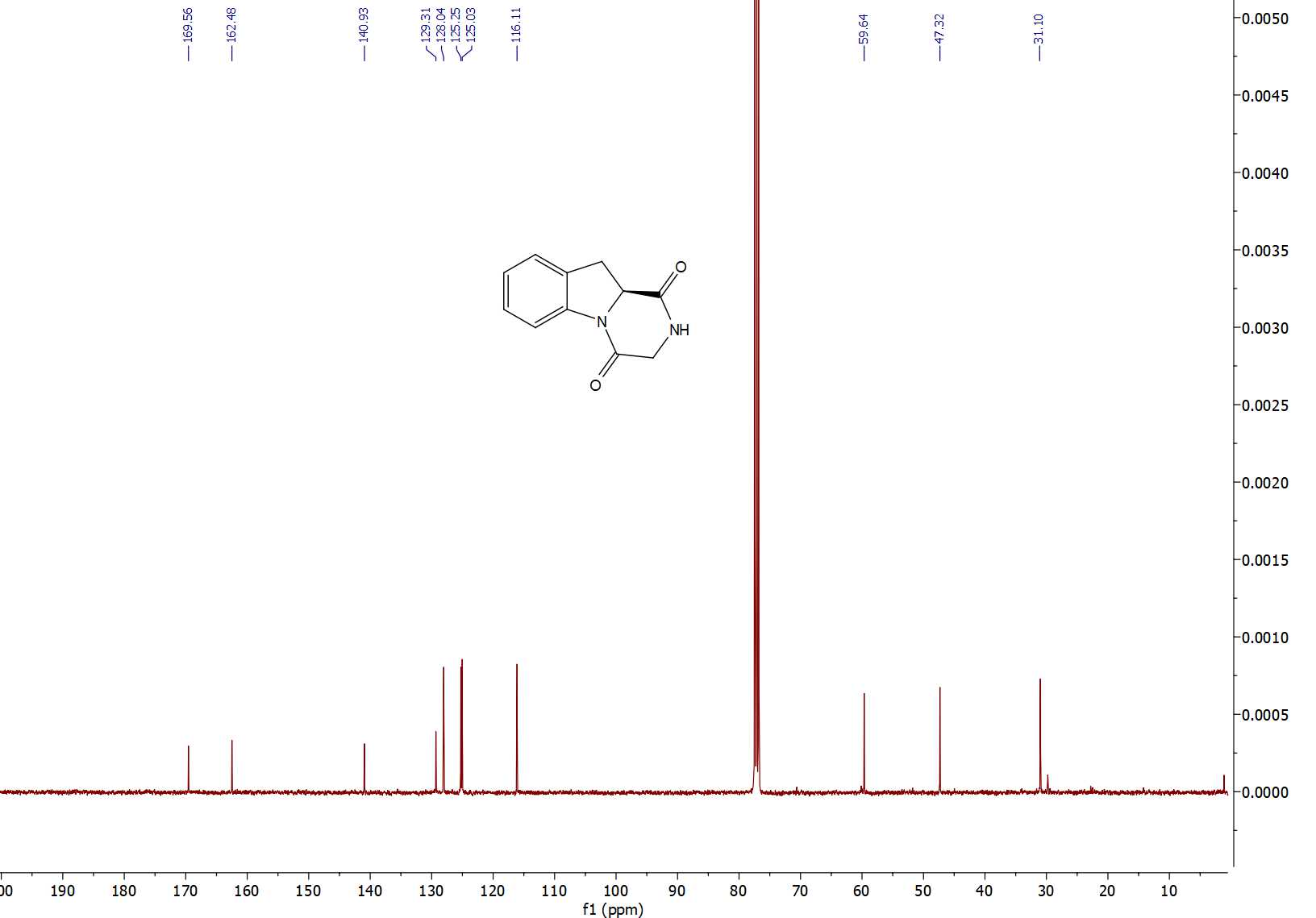
***

### ^1^H NMR of 2,5-diketopiperazine: Product **21** in CDCl_3_

***
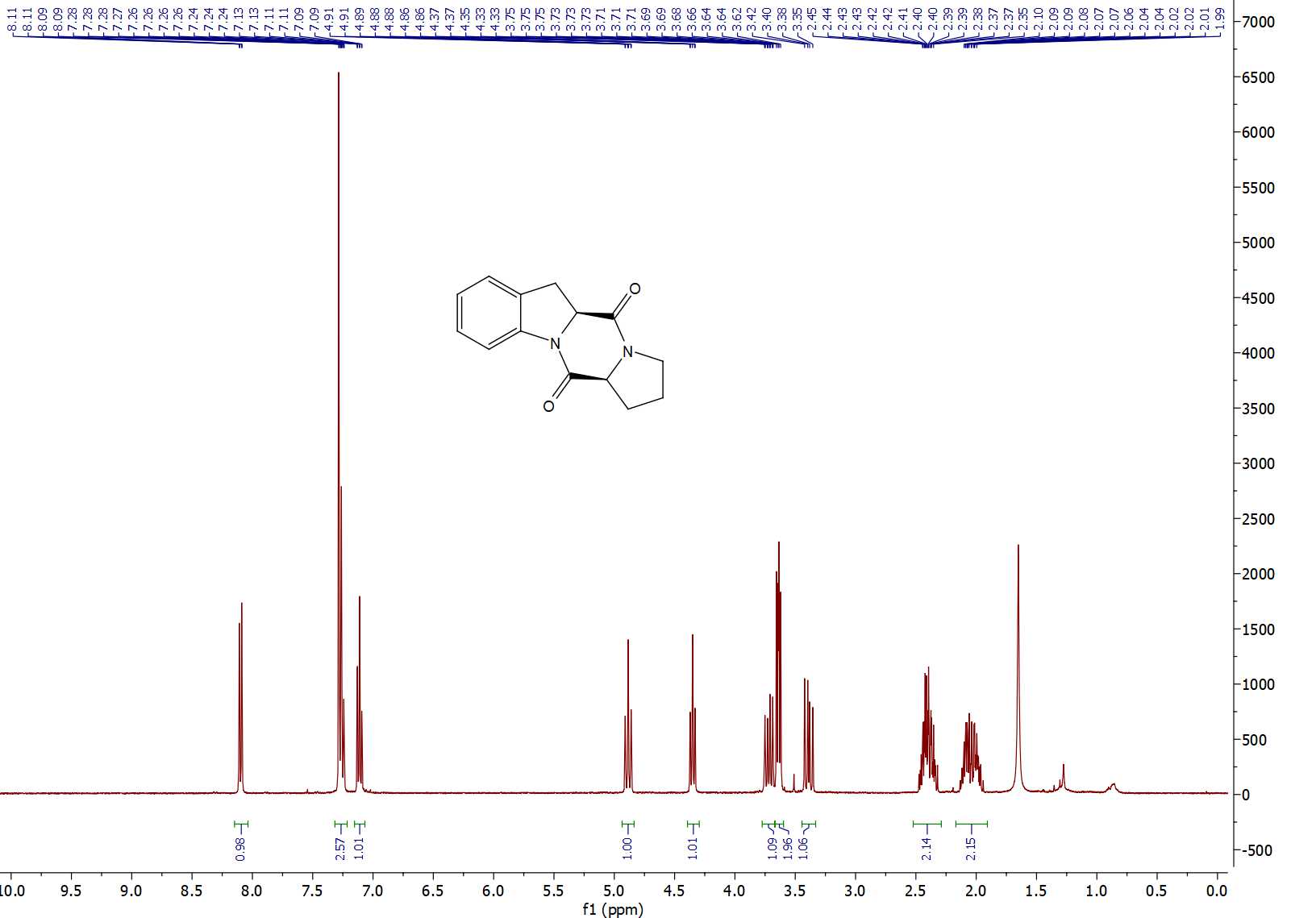
***

### ^1^H NMR of Cbz-l-Pro-(2*S*)-Ind-OH (**22**) in CDCl_3_

***
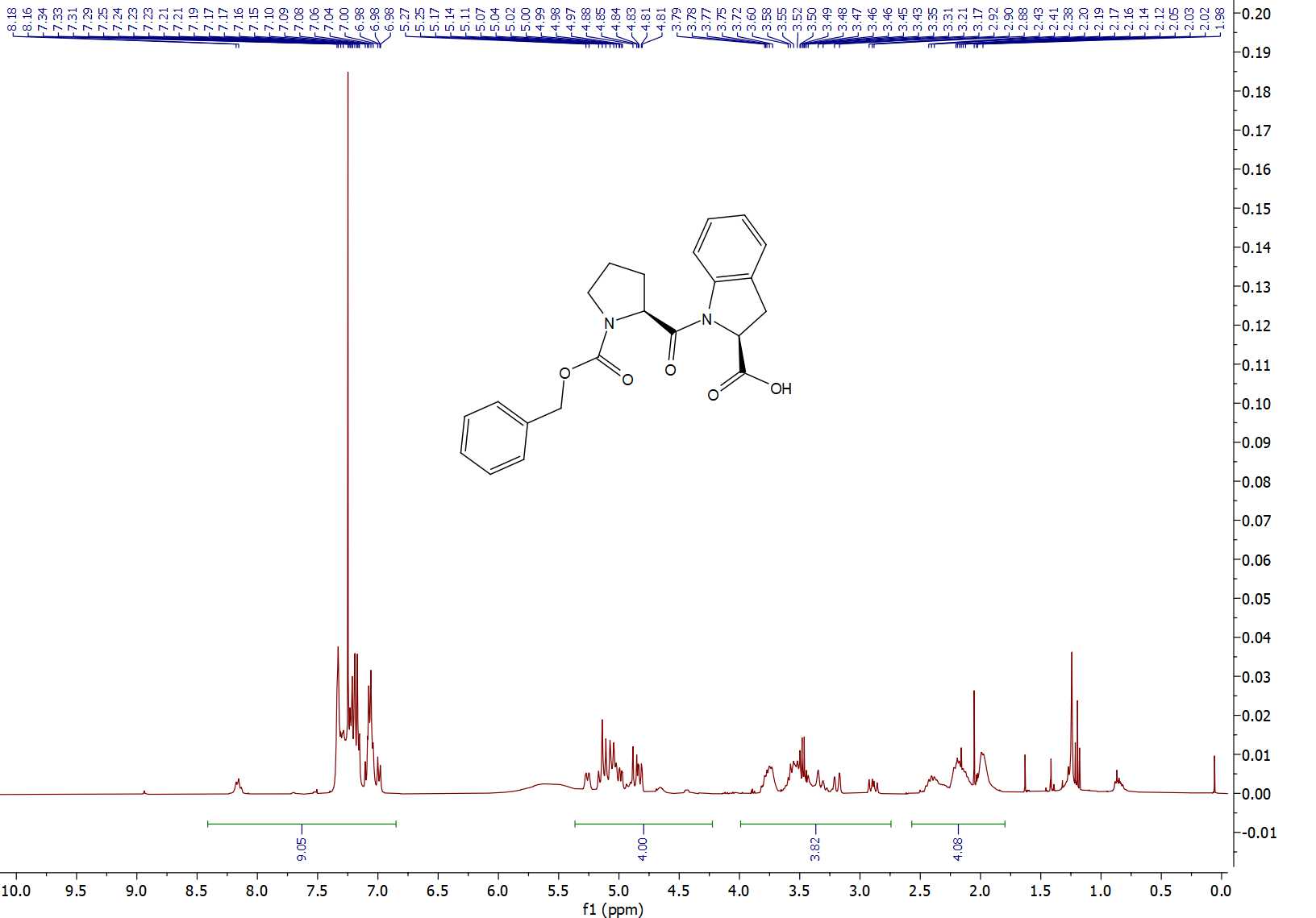
***

### DEPT ^13^C NMR of Cbz-l-Pro-(2*S*)-Ind-OH (**22**) in CDCl_3_

***
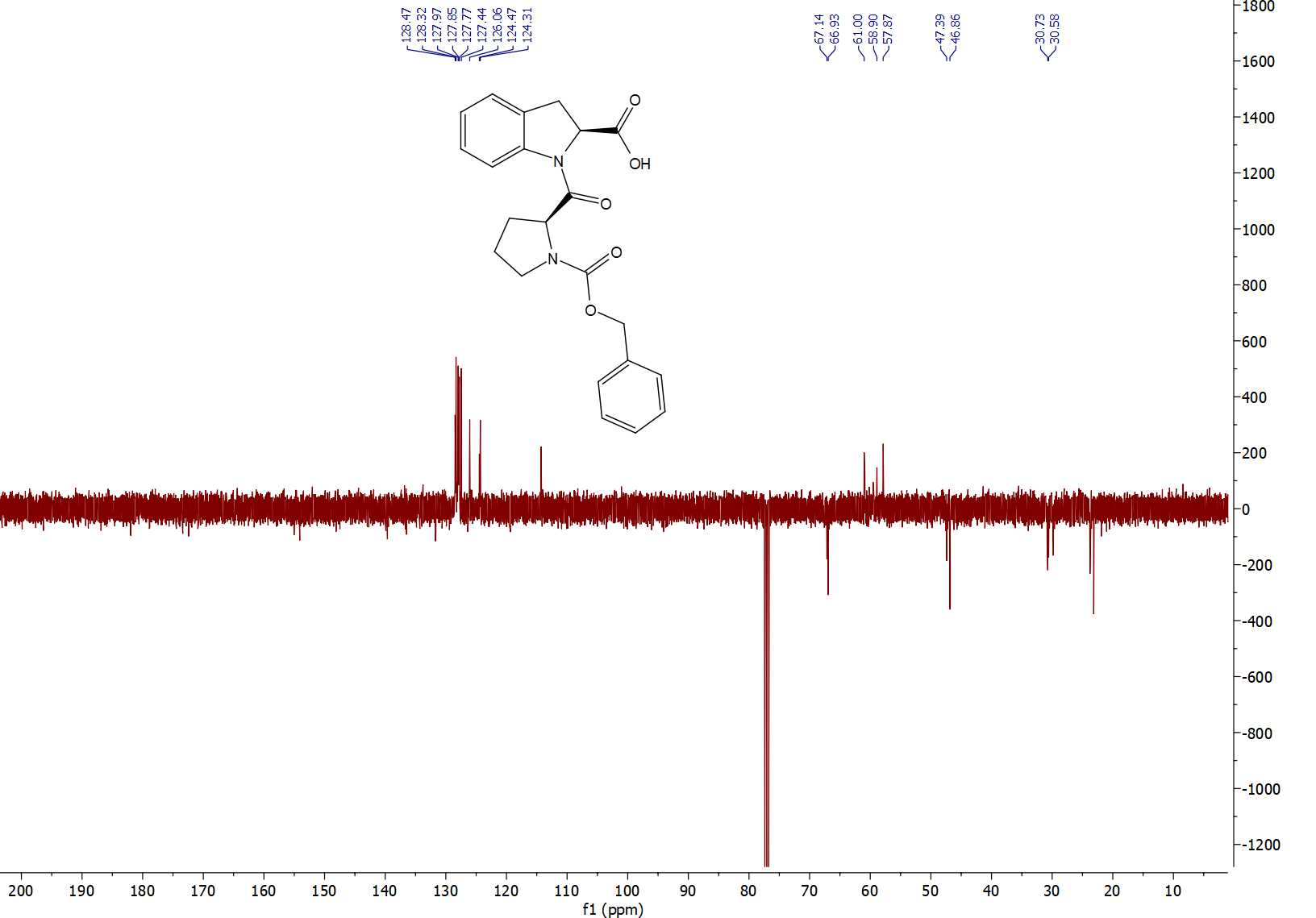
***

### ^1^H NMR of Cbz-l-Pro-(2*S*)-Ind-l-Pro-OMe (**23**) in CDCl_3_

***
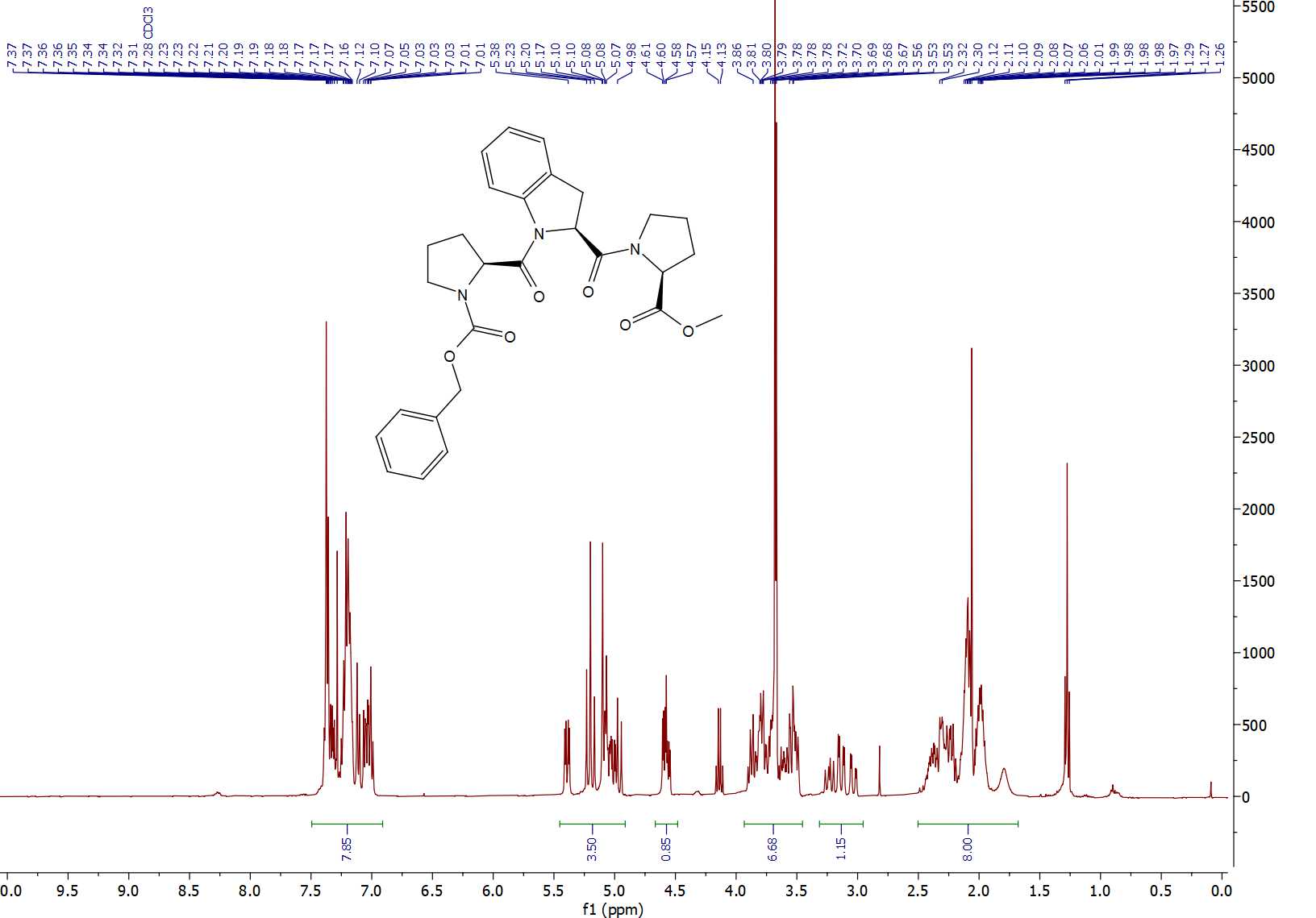
***

### DEPT ^13^C NMR of Cbz-l-Pro-(2*S*)-Ind-l-Pro-OMe (**23**) in CDCl_3_

***
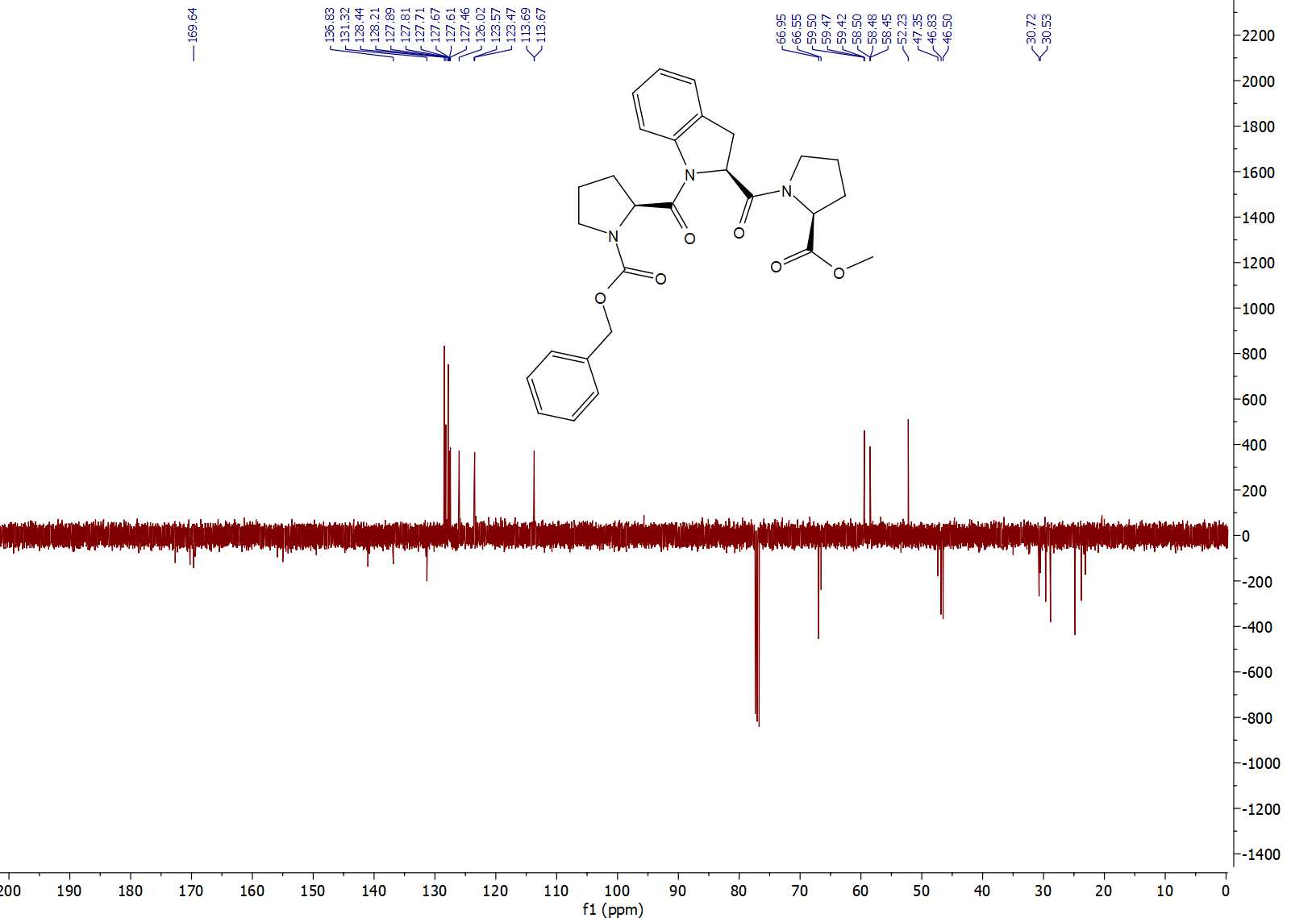
***

### ^1^H NMR of HN-l-Pro-(2*S*)-Ind-l-Pro-OMe (**24**) in CDCl_3_

***
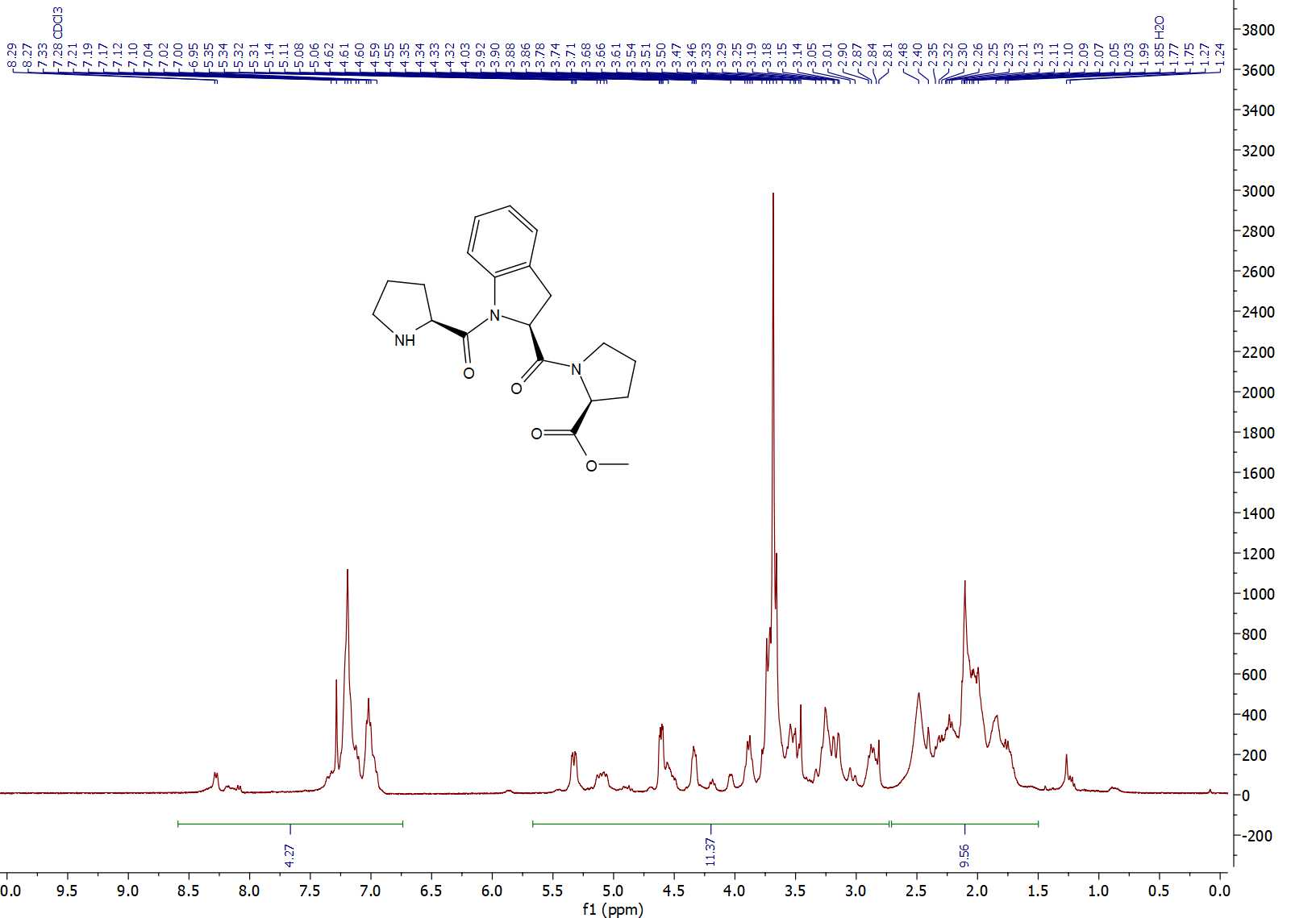
***

### DEPT ^13^C NMR of HN-l-Pro-(2*S*)-Ind-l-Pro-OMe (**24**) in CDCl_3_

***
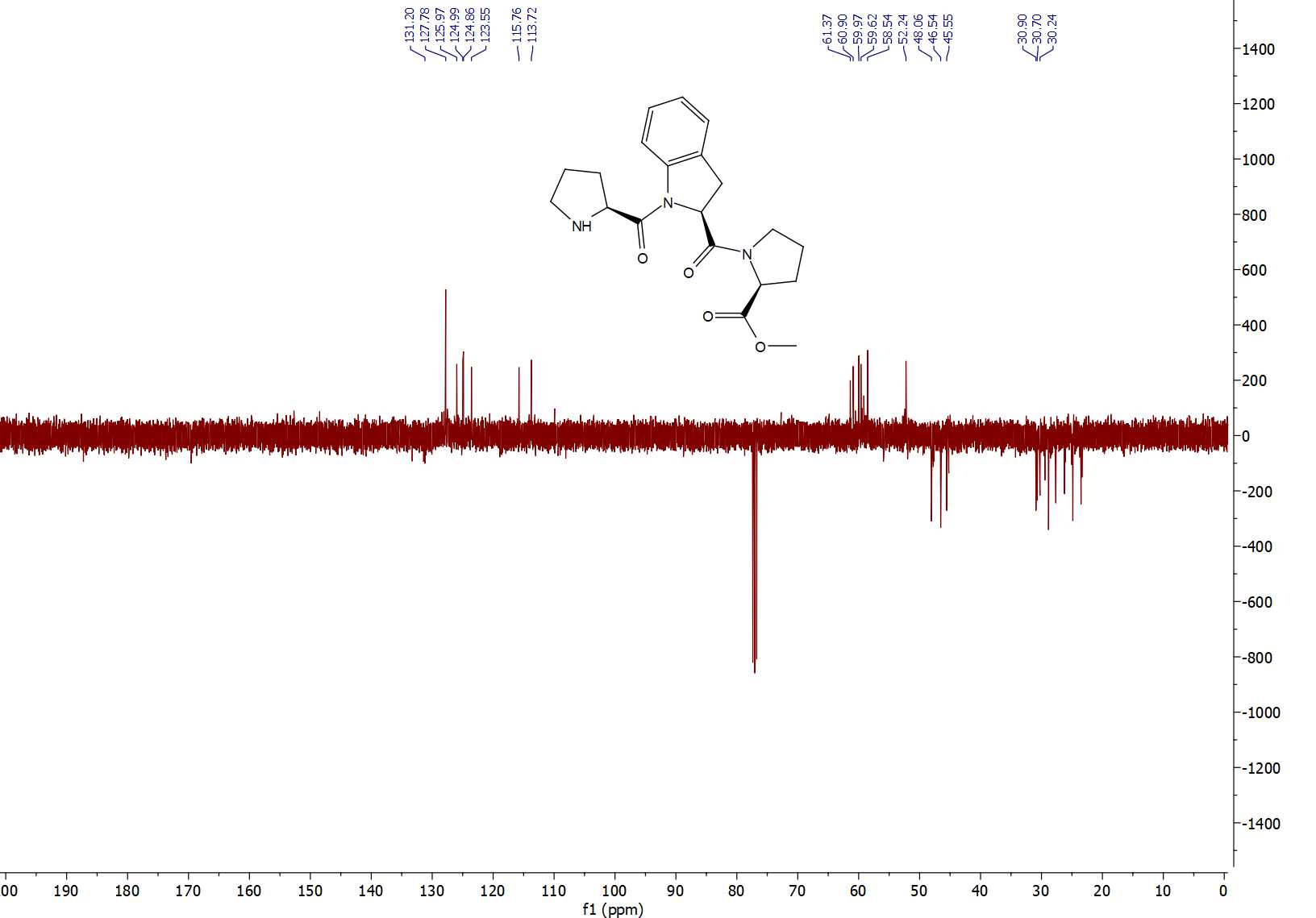
***

## HPLC-DAD and Mass spectra

### HPLC-MS of Ac-(2*S*)-Ind-l-Ala-OMe (**6**)


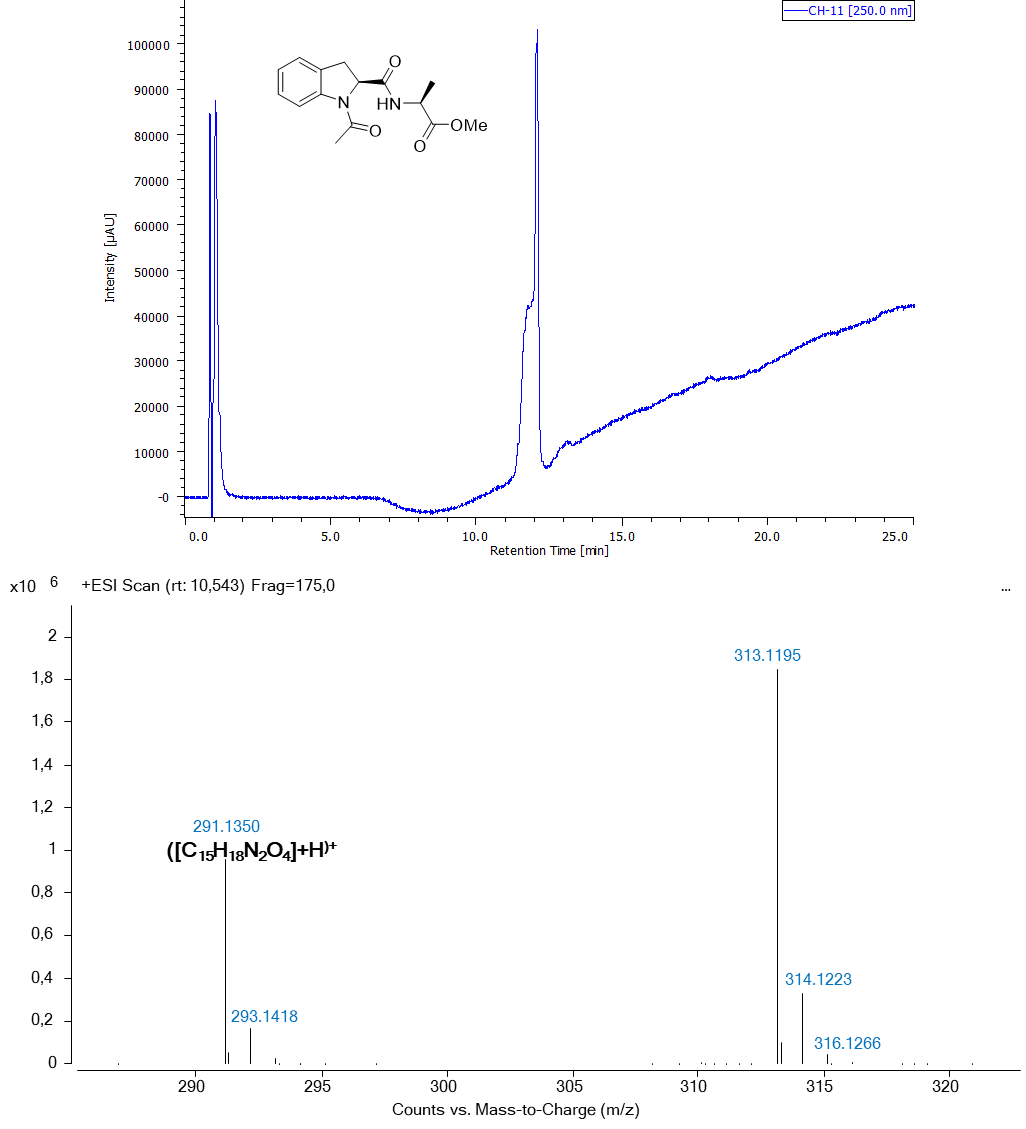


### HPLC-MS of Boc-(2*S*)-Ind-l-Ala-OMe (**7**)

**
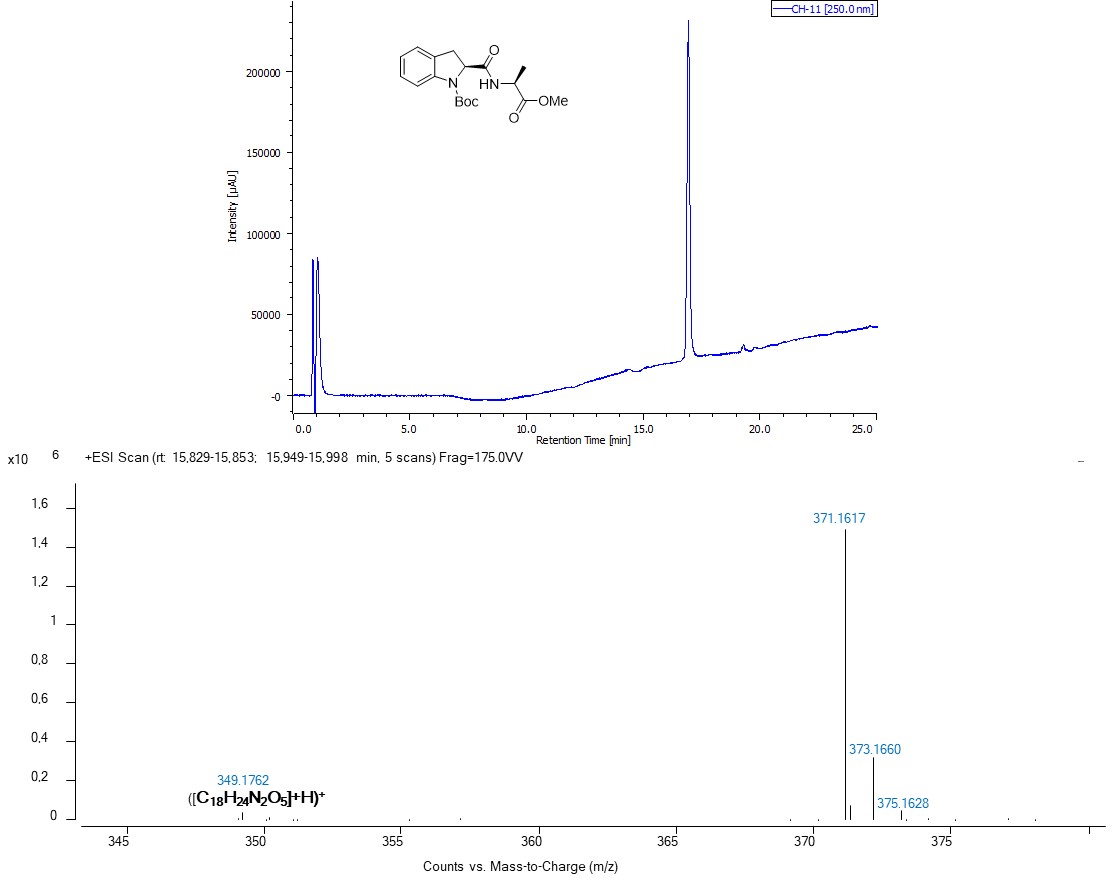
**

### HPLC-MS of Ac-(2*S*)-Ind-d-Ala-OMe (**8**)


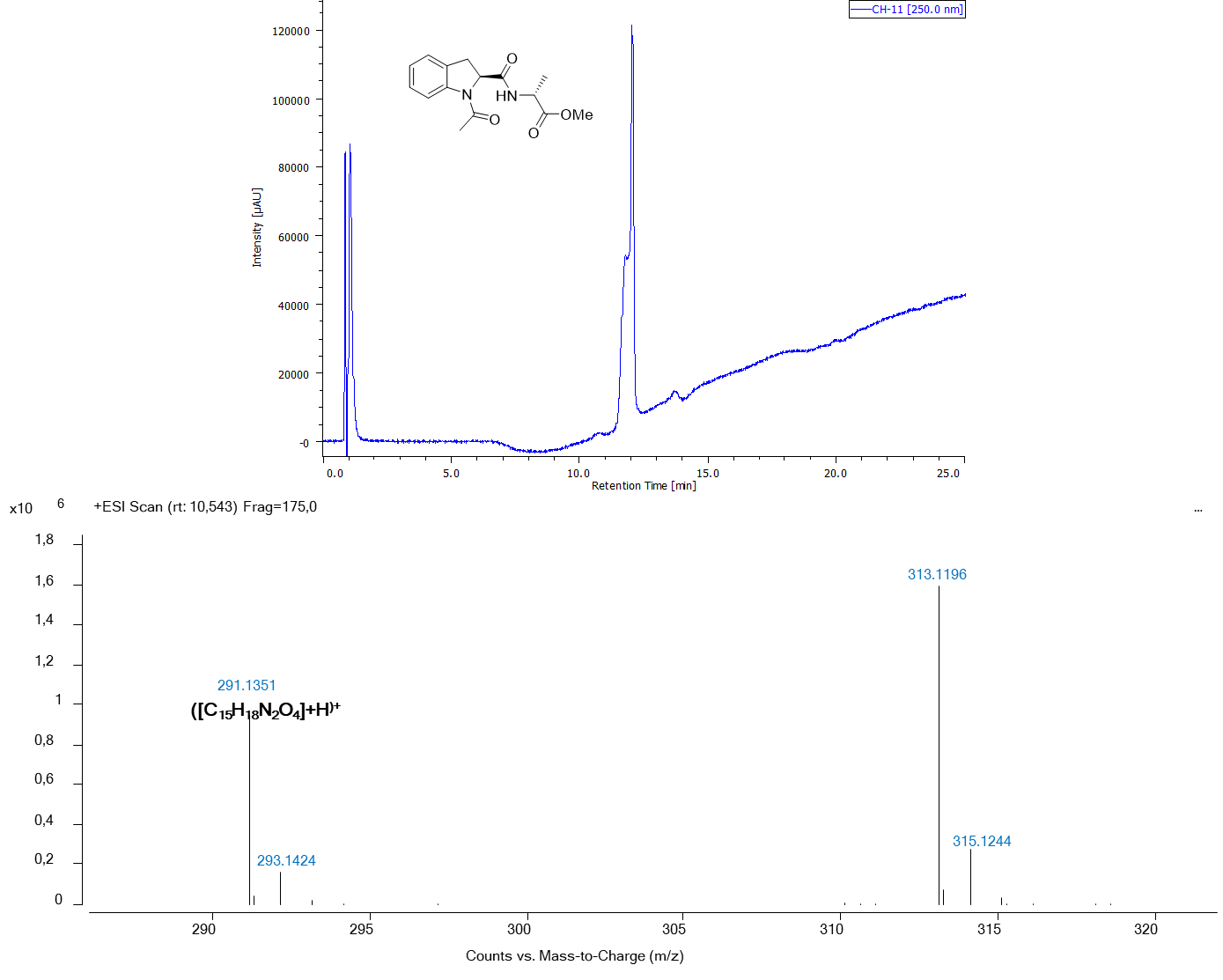


### HPLC-MS of Boc-(2*S*)-Ind-d-Ala-OMe (**9**)

**
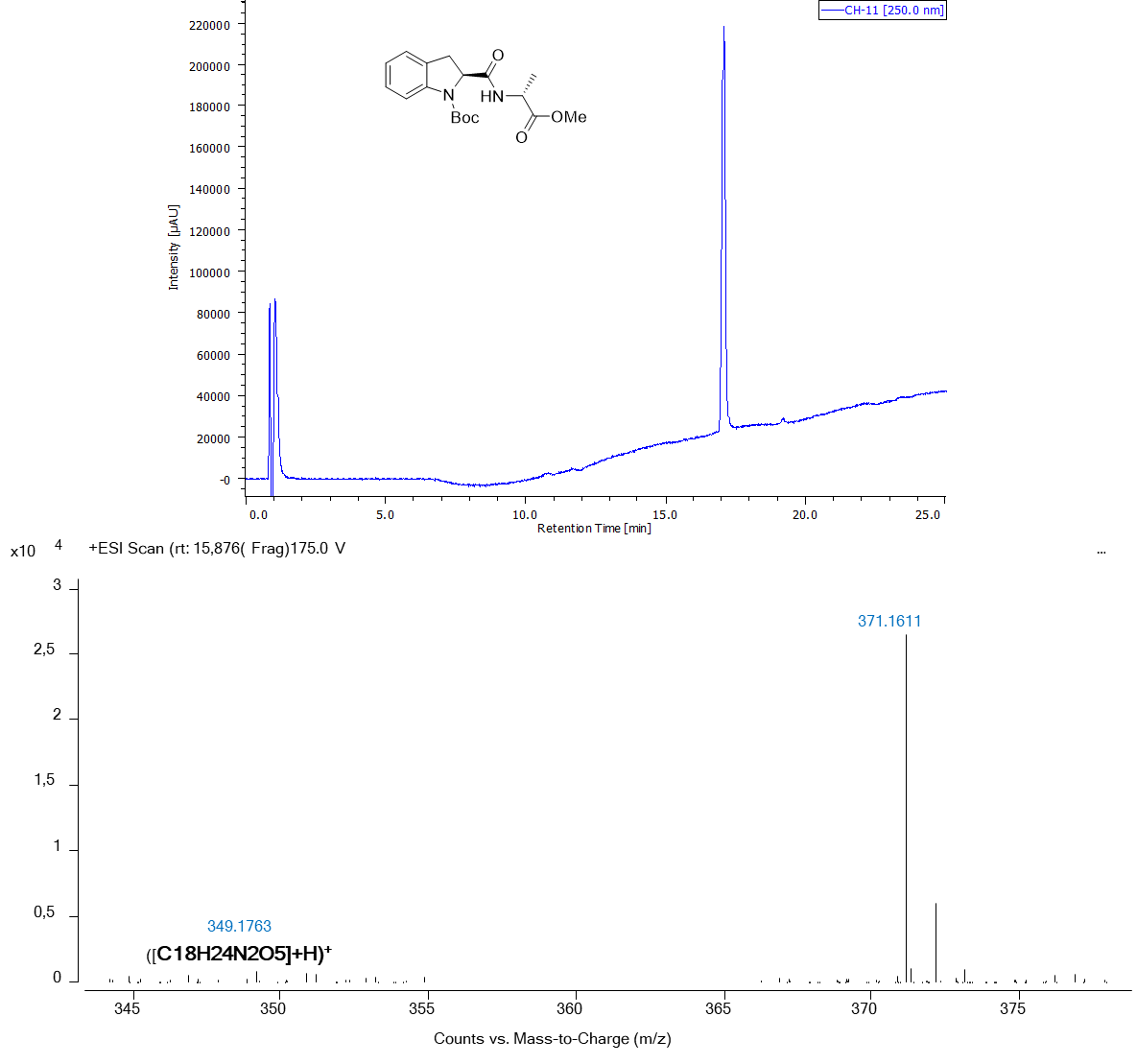
**

### HPLC-MS of Boc-Gly-(2*S*)-Ind-OMe (**13**)

**
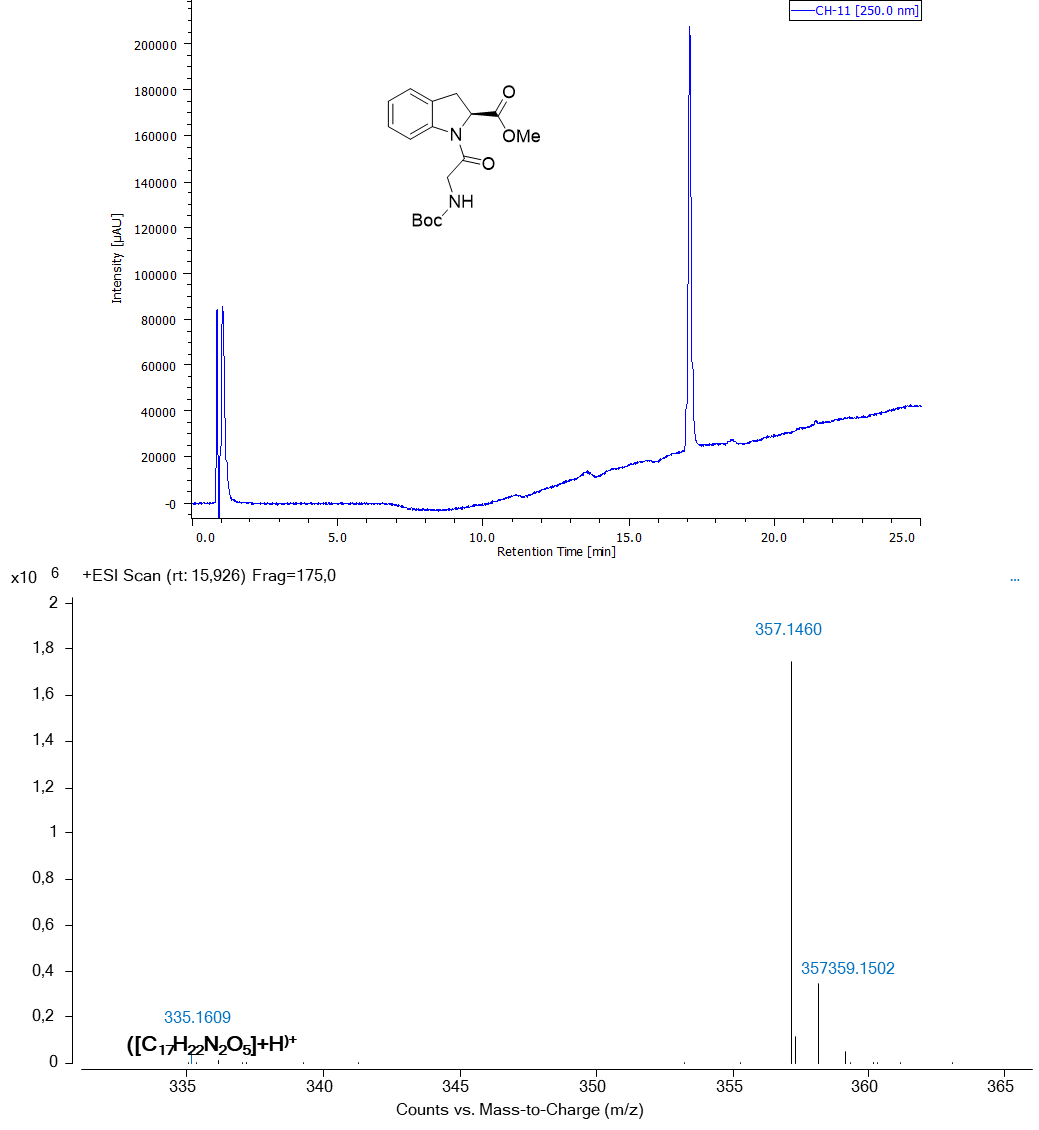
**

### HPLC-MS of Cbz-Gly-(2*S*)-Ind-OMe (**14**)

**
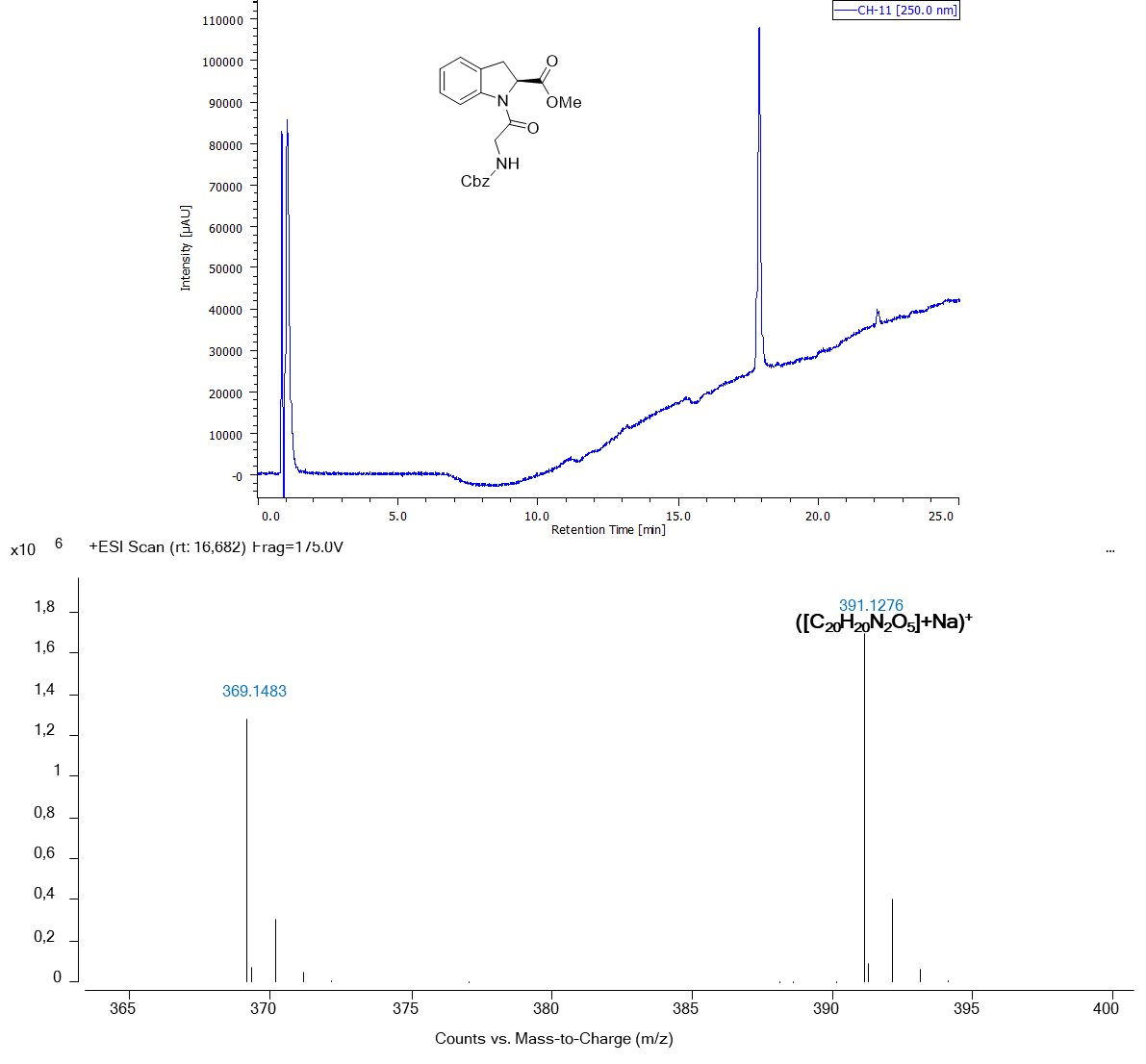
**
